# Functionalization of Tetraphosphido Ligands by Heterocumulenes

**DOI:** 10.1021/acs.inorgchem.4c00808

**Published:** 2024-05-31

**Authors:** Sebastian Hauer, Gábor Balázs, Fabian Gliese, Florian Meurer, Thomas M. Horsley Downie, Christoph Hennig, Jan J. Weigand, Robert Wolf

**Affiliations:** † Institute of Inorganic Chemistry, University of Regensburg, 93040 Regensburg, Germany; ‡ European Synchrotron Radiation Facility, Rossendorf Beamline (BM20-CRG), 38043 Grenoble, France; § Institute of Resource Ecology, Helmholtz-Zentrum Dresden-Rossendorf, 01314 Dresden, Germany; ∥ Faculty of Chemistry and Food Chemistry, Technische Universität Dresden, 01062 Dresden, Germany

## Abstract

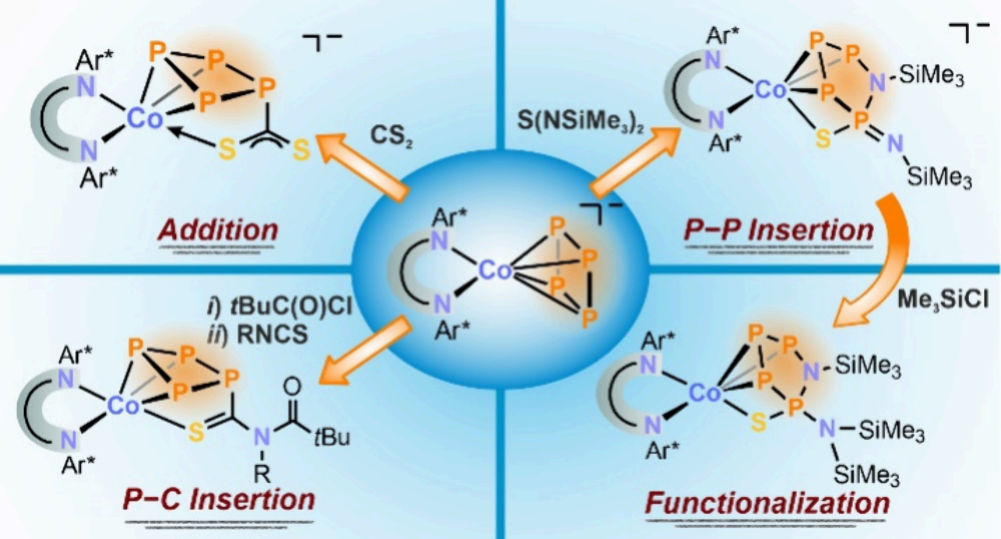

Although numerous polyphosphido complexes have been accessed through the transition-metal-mediated activation and functionalization of white phosphorus (P_4_), the selective functionalization of the resulting polyphosphorus ligands in these compounds remains underdeveloped. In this study, we explore the reactions between cyclotetraphosphido cobalt complexes and heterocumulenes, leading to functionalized P_4_ ligands. Specifically, the reaction of carbon disulfide (CS_2_) with [K(18c-6)][(Ar*BIAN)Co(η^4^-P_4_)] ([K(18c-6)]**1**, 18c-6 = [18]crown-6) affords the adduct [K(18c-6)][(Ar*BIAN)Co(η^3^:η^1^-P_4_CS_2_)] ([K(18c-6)]**3**), in which CS_2_ is attached to a single phosphorus atom (Ar* = 2,6-dibenzhydryl-4-isopropylphenyl, BIAN = 1,2-bis(arylimino)acenaphthene diimine). In contrast, the insertion of bis(trimethylsilyl)sulfur diimide S(NSiMe_3_)_2_ into a P–P bond of [K(18c-6)]**1** yields [K(18c-6)][(Ar*BIAN)Co(η^3^:η^1^-P_4_SN_2_(SiMe_3_)_2_)] (K(18c-6)]**4**). This salt further reacts with Me_3_SiCl to form [(Ar*BIAN)Co(η^3^:η^1^-P_4_SN_2_(SiMe_3_)_3_] (**5**), featuring a rare azatetraphosphole ligand. Moreover, treatment of the previously reported complex [(Ar*BIAN)Co(η^3^:η^1^-P_4_C(O)*t*Bu)] (**2**) with isothiocyanates results in P–C bond insertion, yielding [(Ar*BIAN)Co(η^3^:η^1^-P_4_C(S)N(R)C(O)*t*Bu)] (**6a**,**b**; R = Cy, Ph).

## Abstract 2

Divergent reaction patterns were observed for the functionalization of cyclotetraphosphido ligands in cobalt complexes. Reactions of neutral and anionic complexes with electrophilic heterocumulenes (CS_2_, S(NSiMe_3_)_2_, and RNCS) resulted in the addition of the heterocumulene to the cyclo-P_4_ framework as well as insertions into endocyclic P−P bonds and exocyclic P−C bonds. The structural and NMR spectroscopic characteristics of the products are analyzed, and attempts to further derivatize selected compounds are presented.

## Introduction

**Figure 1:**
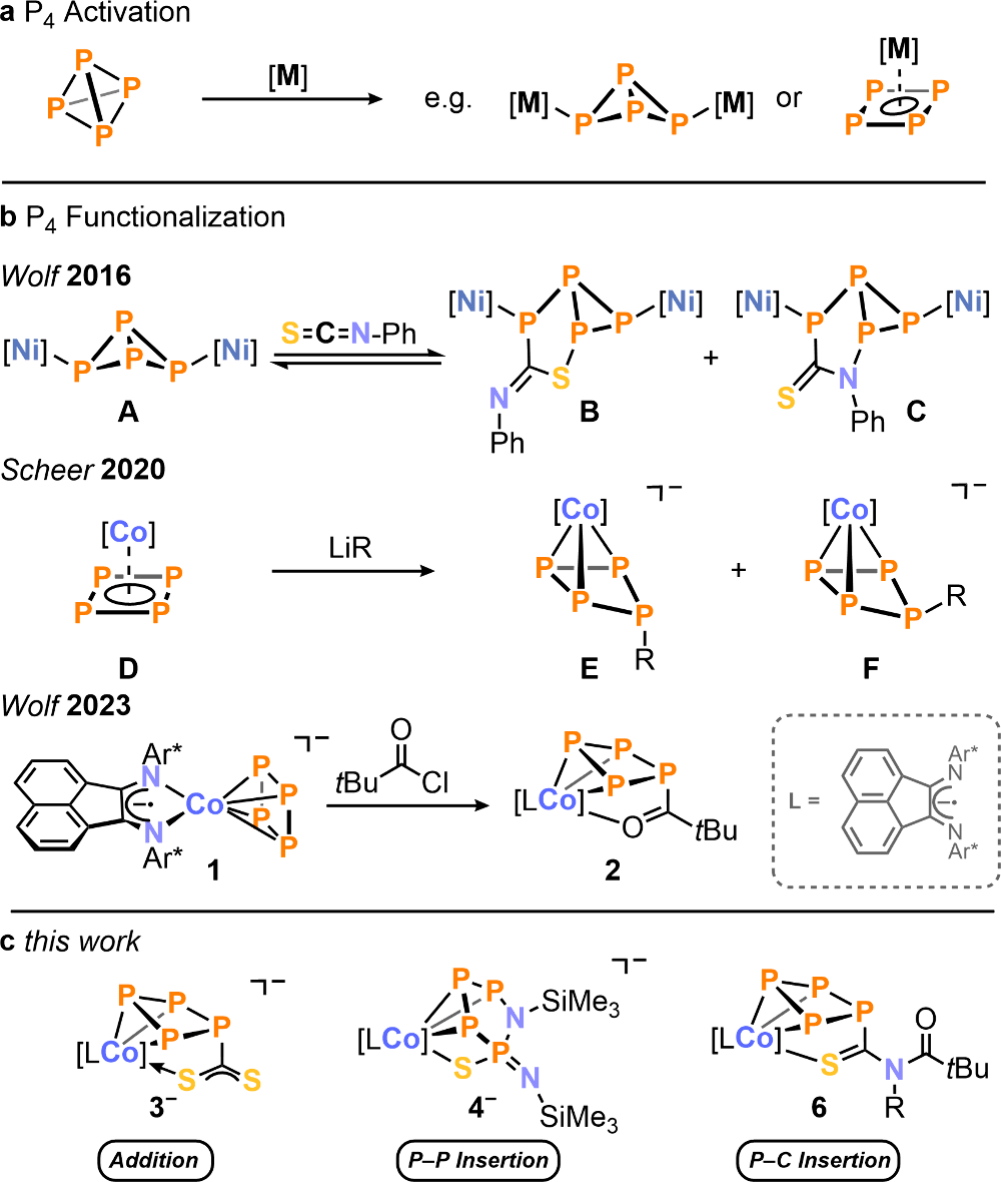
(a) Activation and (b) functionalization of white phosphorus; [Ni] = [CpNi(IMes)] (IMes = 1,3-bis(2,4,6-trimethylphenyl)imidazolin-2-ylidene); [Co] = Cp‴Co (Cp‴ = C_5_H_2_*t*Bu_3_), R = CH_2_SiMe_3_, *t*Bu; Ar* = 2,6-dibenzhydryl-4-isopropylphenyl; (c) [LCo] = (Ar*BIAN)Co, R = Cy, Ph.

The reaction of white phosphorus with transition metal complexes represents a powerful strategy in the synthesis of distinctive phosphorus-based compounds.^1^ Transition-metal-mediated P_4_ functionalization processes typically involve two principal steps, which have been subject to considerable investigation: Initially, a transition metal complex facilitates the cleavage of one or more P–P bonds of the P_4_ tetrahedron, yielding metal complexes that incorporate an activated polyphosphido ligand. Subsequently, these P_*n*_ units undergo functionalization through reactions with suitable nucleophiles or electrophiles. The first step, P_4_ activation, has been widely investigated and can result in a wide variety of polyphosphorus structures, with ligands containing from one to eight P atoms.^1^ In particular, P_4_ ligands such as [1.1.0]bicyclotetraphosphane-1,4-diide (commonly referred to as “butterfly-P_4_^2–^”) and cyclotetraphosphide (*cyclo*-P_4_^2–^) units, emerge as prevalent structural motifs (see Figure 1a).^2,3^ The subsequent functionalization of the coordinated P_*n*_ units typically constitutes a separate step.^1^ While the reactivity in this step can vary based on the electronic properties of the transition metal fragment, it is generally acknowledged that functionalization of P_4_ has not been as thoroughly explored as its activation.

Several routes for the functionalization of butterfly-P_4_ complexes have been reported, including reactions such as the addition and insertion of nucleophiles and electrophiles (including alkylation), fragmentation, and transition metal coordination.^2,4^ During our prior work, in which we reported the synthesis of the nickel butterfly-P_4_ complex **A**, it was found that phenyl isothiocyanate (PhNCS) inserts into a P–P bond of the butterfly moiety. This reaction facilitated the formation of unusual bicyclo[3.1.0]heterohexane isomers **B** and **C** (Figure 1b).^2d,5^

While several *cyclo*-P_4_ complexes have been reported, their reactivity has not been as extensively investigated as the butterfly-P_4_ counterparts.^1,3^ A study by Scheer and co-workers demonstrated that treatment of the *cyclo*-P_4_ complex [Cp‴Co(η^4^-P_4_)] (**D**, Cp‴ = C_5_H_2_*t*Bu_3_) with carbon-centered nucleophiles leads to isomeric compounds **E** and **F** (Figure 1b).^6^

In a recent study, we reported the anionic *cyclo*-P_4_ complex [(Ar*BIAN)Co(η^4^-P_4_)]^−^ (**1**^–^, Figure 1b) and its reaction with acyl chlorides, yielding the functionalized *cyclo*-P_4_ complex [(Ar*BIAN)Co(η^3^:η^1^-P_4_C(O)*t*Bu] (**2**).^7^ When compound **2** was treated with nitriles or isocyanides, there was a partial displacement of the P_4_C(O)R ligand from the cobalt center. Moreover, reaction with 2 equiv of KCN induced a [3 + 1] fragmentation process, releasing a monophosphorus species in the form of an acylcyanophosphide.

Our previous work has revealed that polyphosphido complexes exhibit promising reactivity toward electrophiles and nucleophiles, indicating that transition-metal-P_*n*_ complexes hold potential as precursors for the targeted synthesis of unique (poly-)phosphorus compounds (Figure 1).^5,8^ Building upon these insights, we herein report the functionalization of the anionic complex **1**^–^ and its acylated, neutral counterpart **2** with electrophilic heterocumulenes. In this study, we present the synthesis of anionic cobalt complexes **3**^–^ and **4**^–^, featuring CoP_4_CS_2_^–^ and Co(η^3^:η^1^-P_4_SN_2_(SiMe_3_)_2_)^−^ cores, respectively. We investigate their structural and electronic properties, as revealed by X-ray crystallography using synchrotron radiation and density functional theory (DFT). Additionally, the reactivity of complexes **3**^–^ and **4**^–^ toward electrophiles in salt metathesis is examined. We also demonstrate the feasibility of further functionalizing the polyphosphido ligand in CoP_4_C(O)*t*Bu with isothiocyanates, resulting in the formation of [Co(η^3^:η^1^-P_4_C(S)N(R)C(O)*t*Bu)] complexes (**6**, R = Cy, Ph).

## Results and Discussion

**Scheme 1:**
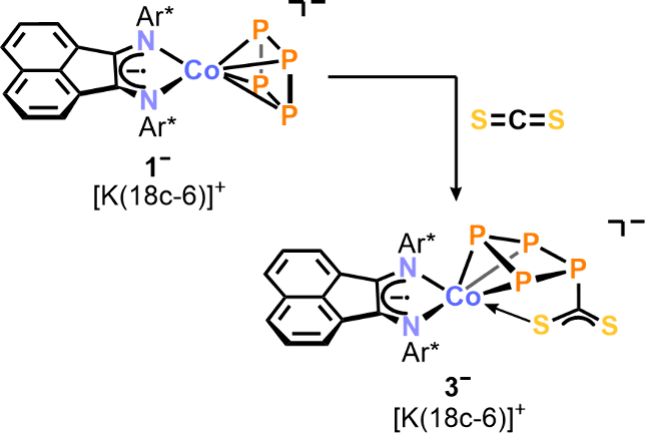
Addition of CS_2_ to the Tetraphosphido Ligand in [K(18c-6)]**1** Reagents and conditions: 1.2 equiv of CS_2_; THF, r.t., 1 d; yield: [K(18c-6)]**3**: 89%.

The addition of carbon disulfide to a purple solution of [K(18c-6)]**1** in THF resulted in a blue coloration within a few hours. ^31^P{^1^H} NMR spectroscopy confirmed the complete conversion of anionic **1**^–^ into a single new species, [K(18c-6)][(Ar*BIAN)Co(η^3^:η^1^-P_4_CS_2_)] ([K(18c-6)]**3**), exhibiting an AXY_2_ spin system (vide infra). This new complex crystallized in 89% yield as dark blue blocks from a THF/*n*-hexane mixture (Scheme 1).

**Figure 2:**
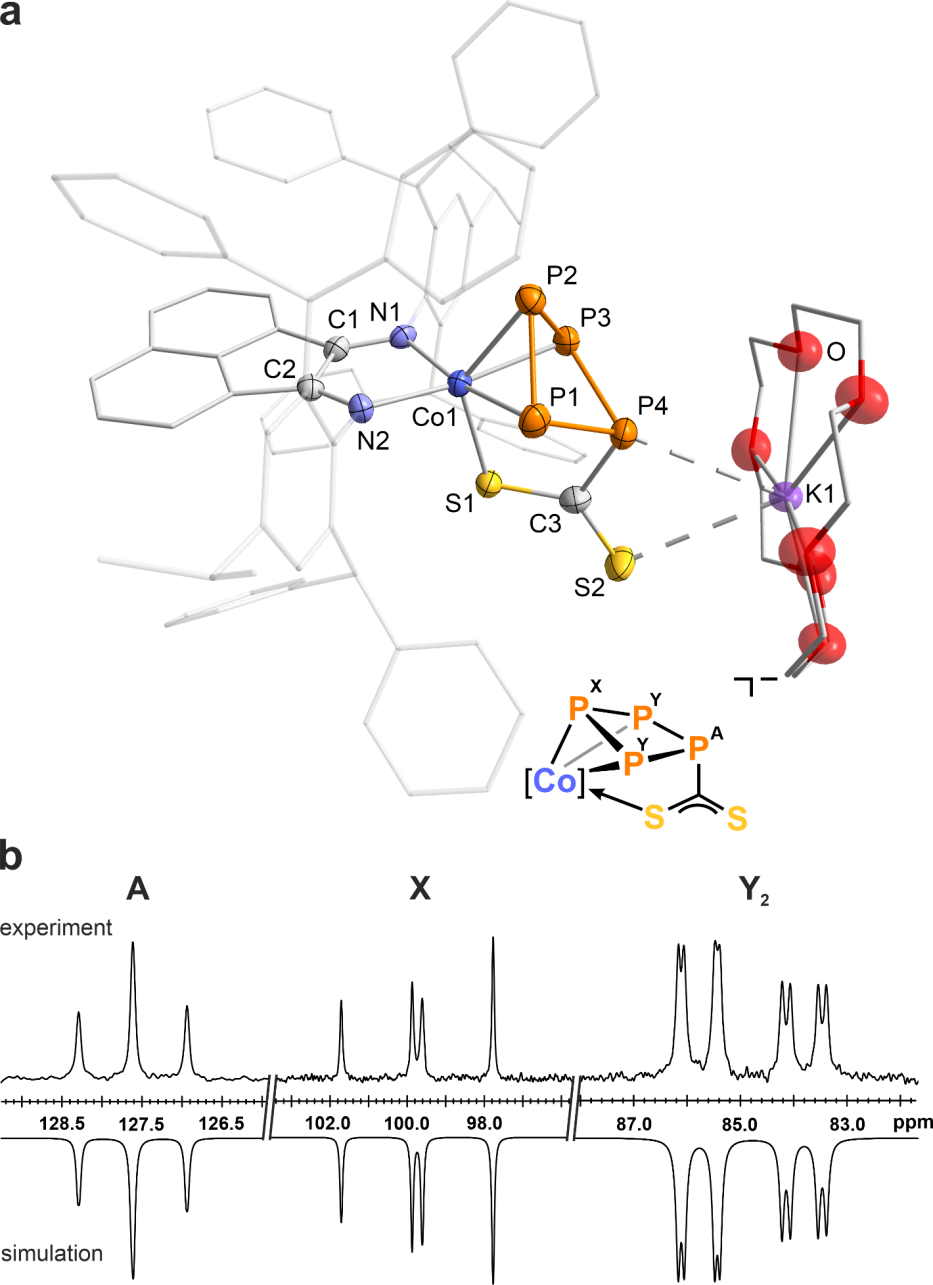
(a) Solid-state molecular structure of [K(18c-6)][(Ar*BIAN)Co(η^3^:η^1^-P_4_CS_2_)] ([K(18c-6)]**3**); thermal ellipsoids are shown at the 50% probability level; hydrogen atoms, solvent molecules, and disorder are omitted for clarity. Selected bond lengths [Å] and angles [deg]: P1–P2 2.169(2), P2–P3 2.1697(2), P3–P4 2.230(2), P1–P4 2.2286(2), Co1–P1 2.2936(1), Co1–P2 2.3031(2), Co1–P3 2.2815(2), Co1–S1 2.2725(1), Co1–N1 1.998(4), Co1–N2 1.976(4), P4–C3 1.850(6), C3–S1 1.698(5), C3–S2 1.664(5), P1–P2–P3 85.08(7), P2–P3–P4 88.93(7), P3–P4–P1 82.44(6), P4–P1–P2 88.81(8). (b) Experimental (upward) and simulated (downward) ^31^P{^1^H} NMR spectra of [K(18c-6)]**3** in THF-*d*_8_ with nuclei assigned to an AXY_2_ spin system: δ(P_A_) = 127.6 ppm, δ(P_X_) = 99.6 ppm, δ(P_Y_) = 84.9 ppm, ^1^*J*_XY_ = −320 Hz, ^1^*J*_AY_ = −110 Hz, ^2^*J*_AX_ = 5 Hz.

Single crystal X-ray diffraction (SCXRD) analysis elucidated the structure of the complex, revealing a puckered *cyclo*-P_4_ ligand in a η^3^-coordinating mode (Figure 2a). A CS_2_ moiety is bound via the carbon atom to the noncoordinating phosphorus atom P4. Additionally, one sulfur atom from the CS_2_ moiety exhibits η^1^-coordination to the cobalt center. The similar C–S bond distances, 1.698(5) Å and 1.664(5) Å, are intermediate between those of typical C–S single and double bonds (∑*r*_CS_ 1.78 Å vs 1.61 Å).^9^ The Co–S1 bond length (2.2724(1) Å) is notably longer than the Co–S distance in the structurally related complex [(triphos)Co(η^2^-CS_2_)] (2.206(4) Å; triphos = MeC(CH_2_PPh_2_)_3_) and exceeds the length of a typical Co–S single bond (∑*r*_CoS_ 2.14 Å).^10^ These observations support the description of the P_4_CS_2_ ligand as featuring a delocalized exocyclic CS_2_-moiety acting as a pendant donor ligand, as depicted in Scheme 1. The delocalization of the η^3^-coordinated P1–P2–P3 moiety is apparent by shorter bond lengths among the coordinating phosphorus atoms (P1–P2 2.169(2) Å and P2–P3 2.169(7) Å) compared to those involving the noncoordinating ones (P1–P4 2.228(6) Å and P3–P4 2.222(7) Å). This structural motif is similar to the behavior observed in the related complex [(Ar*BIAN)Co(η^3^:η^1^-P_4_C(O)*t*Bu)] (**2**, vide infra) and in the series of complexes [Cp‴Co(η^3^-P_4_R_2_)] (R = Ph, Cy, *t*Bu).^7,11^

The ^31^P{^1^H} NMR spectrum of [K(18c-6)]**3** features an AXY_2_ spin system, corroborating the existence of a *C*_*s*_ symmetric tetraphosphido ligand (Figure 2b). The simulated P–P coupling constants are in agreement with those of [Cp‴Co(η^3^-P_4_R_2_)] complexes, reported to exhibit AMM′X spin systems, and the previously reported complex **2**, which gives rise to an AM_2_X spin system.^7,11^ The resonance for the coordinating phosphorus atom P_*x*_ at δ = 99.6 ppm is shifted significantly upfield in comparison to **2** (δ = 323.3 ppm) but appears downfield shifted relative to the equivalent phosphorus atom of [Cp‴Co(η^3^-P_4_Ph_2_)] (δ = −80.7 ppm).

**Scheme 2:**
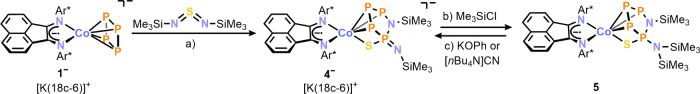
Reaction of [K(18c-6)]**1** with Sulfur Diimide and Subsequent Functionalization with Trimethylsilyl Chloride Reagents/byproducts and conditions: (a) 1.5 equiv of S(NSiMe_3_)_2_; THF, 35 °C, 6 d; (b) Me_3_SiCl/ –[K(18c-6)]Cl; toluene, r.t., 3 h; (c) 1.0 equiv of [*n*Bu_4_N]CN; C_6_D_6_, r.t., 3 h or 1.0 equiv of KOPh/1.0 equiv of 18c-6; C_6_D_6_, r.t., 3 d; yields [K(18c-6)]**4**: 63%, **5**: 63%.

Inspired by the successful functionalization of the *cyclo*-P_4_ ligand in **1**^–^ with CS_2_ (vide supra), we extended our investigation to include reactions with other heterocumulenes. While attempts to functionalize the P_*n*_ moieties with isocyanates and isothiocyanates led to complex mixtures of products that impeded characterization, the use of sulfur diimide S(NSiMe_3_)_2_ resulted in the selective formation of [K(18c-6)][(Ar*BIAN)Co(η^3^:η^1^-P_4_SN_2_(SiMe_3_)_2_)] ([K(18c-6)]**4**, Scheme 2a). ^31^P{^1^H} NMR spectroscopic monitoring revealed a quantitative reaction and complete conversion within 6 days at 35 °C, using a slight excess of the diimide (1.5 equiv). Surprisingly, the diimide variant with alkyl substituents, S(N*t*Bu)_2_, did not undergo any reaction under similar conditions, or at further elevated temperature.

**Figure 3:**
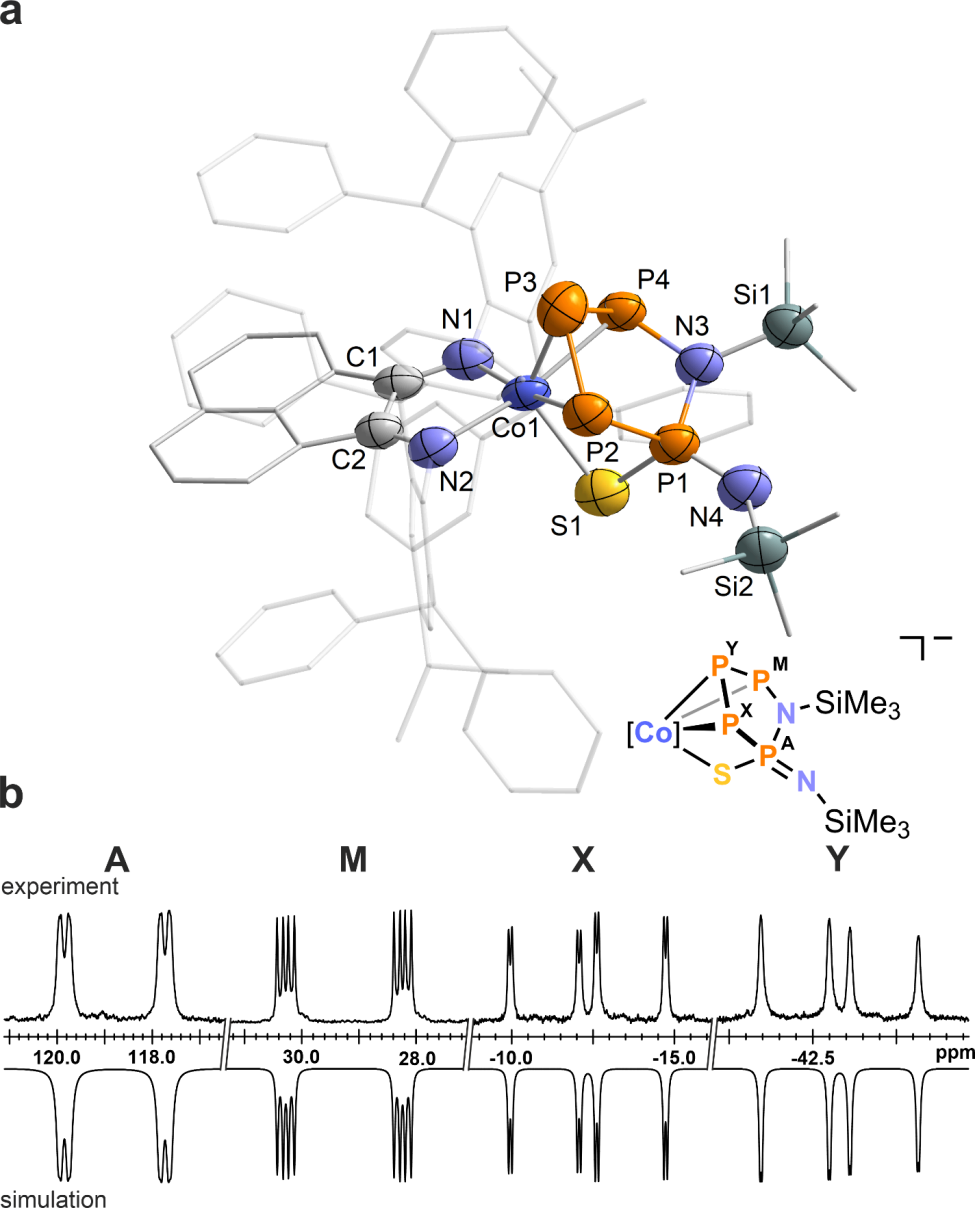
(a) Solid-state molecular structure of [K(18c-6)][(Ar*BIAN)Co(η^3^:η^1^-P_4_SN_2_(SiMe_3_)_2_)] ([K(18c-6)]**4**); thermal ellipsoids are shown at the 50% probability level; hydrogen atoms, solvent molecules, [K(18c-6)]^+^, and disorder are omitted for clarity. Selected bond lengths [Å] and angles [deg]: P1–P2 2.205(2), P2–P3 2.047(2), P3–P4 2.200(8), P1–N3 1.681(4), P4–N3 1.749(5), P1–S1 2.049(2), P1–N4 1.567(5), Co1–P2 2.336(2), Co1–P3 2.235(2), Co1–P4 2.327(2), Co1–S1 2.391(2), P1–P2–P3 103.00(7), P2–P3–P4 95.57(8), P3–P4–N3 104.75(2), P4–N3–P1 109.5(3), Co1–S1–P1 81.90(8), Si1–N3–P1 124.6(3), Si2–N4–P1 134.0(3). (b) Experimental (upward) and simulated (downward) ^31^P{^1^H} NMR spectra of **4**^–^ with nuclei assigned to an AMXY spin system: δ(P_A_) = 118.8 ppm, δ(P_M_) = 29.2 ppm, δ(P_X_) = −12.4 ppm, δ(P_Y_) = −43.2 ppm, ^1^*J*_XY_ = −431 Hz, ^1^*J*_AX_ = −343 Hz, ^1^*J*_MY_ = −331 Hz, *J*_MX_ = 17 Hz, *J*_AY_ = 10 Hz, *J*_AM_ = −32 Hz.

A SCXRD analysis, using synchrotron radiation at the Rossendorf Beamline BM20 (ESRF), conducted on crystals obtained from a toluene/*n*-hexane mixture, revealed the structure of anion **4**^–^, featuring an η^3^-coordinating azatetraphosphole ring (Figure 3a).^12^ The structure bears an exocyclic NSiMe_3_ group alongside a sulfur atom, both bound to the same phosphorus atom, indicating the insertion of a Me_3_SiN moiety into a P–P bond. The azaphosphole ring adopts an envelope conformation with the nitrogen atom N3 positioned at the apex, at a distance of 0.700(6) Å above the plane formed by P1, P2, P3, and P4. This conformation resembles cyclic P_4_N frameworks observed in oligophosphines such as *cyclo*-[NP(PPh_2_)_2_]_2_, *cyclo*-[(PMe)(PPh_2_)N]_2_, and *cyclo*-[(PPh)_4_NR] (R = Me, Cy),^13d,13h^ as well as related compounds.^13^ To our knowledge, [K(18c-6)]**4** is the first example of a transition metal complex bearing a *cyclo*-P_4_N ligand framework. The P1–P2 and the P3–P4 bond lengths of 2.205(2) Å and 2.200(8) Å, respectively, agree with typical P–P single bonds (∑*r*_PP_ 2.22 Å), whereas the P2–P3 bond length at 2.047(2) Å suggests partial P=P double bond character.^9^ This interpretation is supported by calculated bond orders of 0.89, 1.05, and 1.13, despite the optimized P2–P3 distance in the theoretical models (2.155 Å) being slightly longer than the experimental value (vide infra).

To corroborate the molecular structure derived from SCXRD data, we carried out geometry optimization for anion **4**^–^ using the TPSS-D4/def2-TZVP CPCM level of theory. Subsequent intrinsic bond orbital analysis (IBO, see SI for details) identified single bonds within the cyclic P_4_N moiety and a polarized P=N (P1–N4) double bond (see IBO 155 in Figure S43 in SI).^14^ Additionally, a lone pair was observed on N4, residing in a p-type orbital with slight delocalization over the P_2_N unit, which contributes to the stabilization of the planar geometry at N4. The Mayer bond order (MBO) analysis further supports the double bond character of the P1–N4 bond, with a calculated MBO of 1.57. These theoretical insights align well with the P1–N4 bond length of 1.567(5) Å, which falls in the expected range for a P=N double bond (∑*r*_PN_ 1.62 Å).^9^ Conversely, the MBO value of the endocyclic P1–N3 and P4–N3 bonds are 1.05 and 0.95, respectively, indicative of single bonds.

The ^31^P{^1^H} NMR spectrum of [K(18c-6)]**4** in CD_3_CN exhibits an AMXY spin system, distinguished by large ^1^*J*_PP_ coupling constants ranging from −331 Hz to −431 Hz, with chemical shifts recorded at δ = 118.8 (P_A_), 29.2 (P_E_), −12.4 (P_M_), and −43.2 (P_X_) ppm (Figure 3b and Figure S7, SI). These findings are characteristic for an asymmetric *catena*-P_4_ unit, consistent with previous observations for similar systems.^7,8b,15^ The assignment of the resonances is based on the assumption that the tetrasubstitued P_1_ atom gives rise to the most deshielded ^31^P NMR signals. The largest ^1^*J*_PP_ coupling constant was observed between phosphorus atoms P2 and P3, further supporting partial P=P double bond character. In the ^29^Si{^1^H} NMR spectrum, two distinct doublets emerge: one at δ = −17.9 ppm corresponding to the imino group, and one at δ = 3.6 ppm, assigned to the amino group. These groups feature ^2^*J*_SiP_ coupling constants of 16.6 and 6.1 Hz, respectively. For comparison, the resonance of S(NSiMe_3_)_2_ appears at δ = 1.6 ppm in C_6_D_5_CD_3_.^16^ The more pronounced ^2^*J*_SiP_ coupling associated with the exocyclic NSiMe_3_ group is likely a consequence of its involvement in the P1=N4 multiple bond.

**Figure 4:**
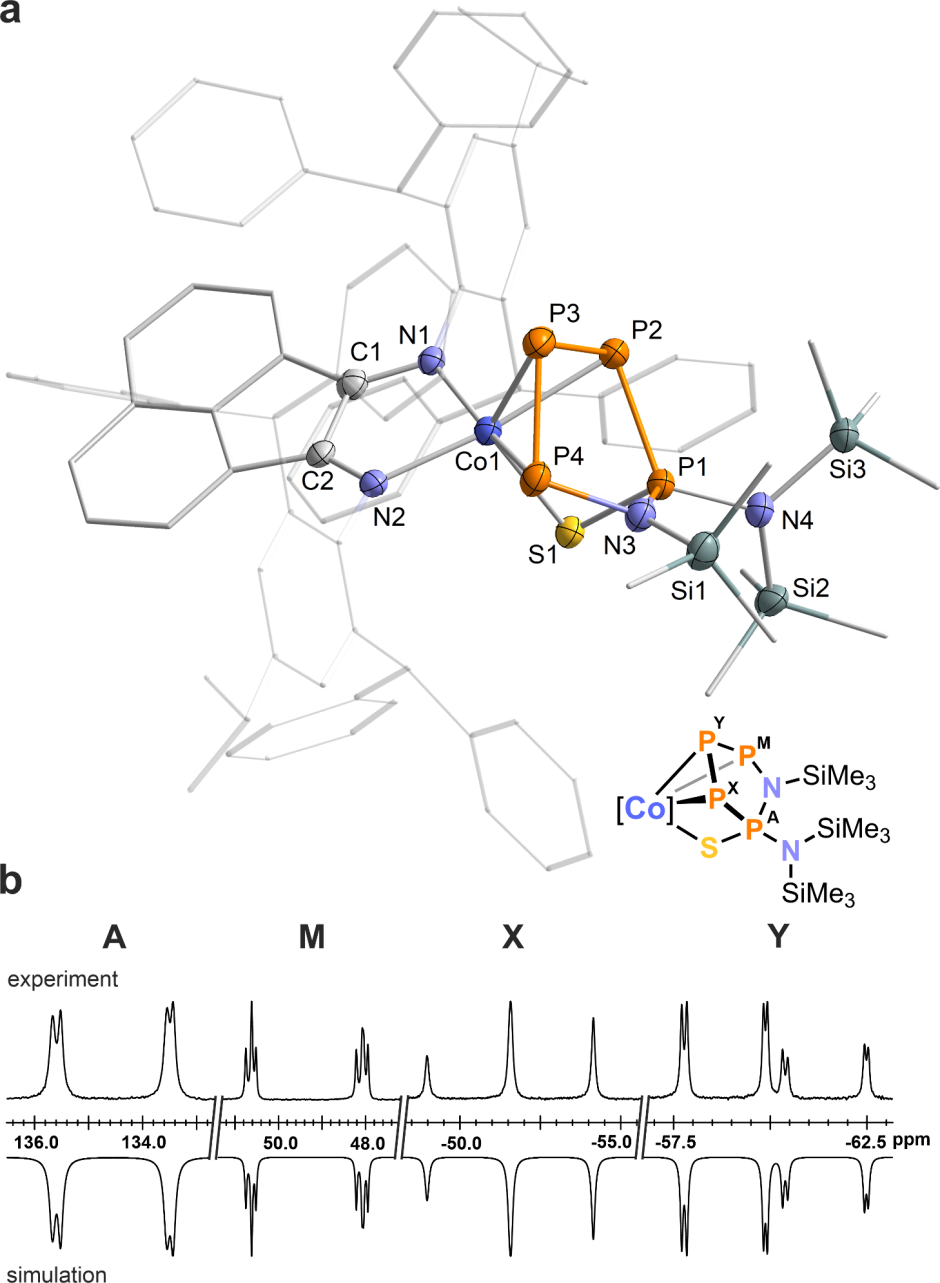
(a) Solid-state molecular structure of [(Ar*BIAN)Co(η^3^:η^1^-P_4_SN_2_(SiMe_3_)_3_)] (**5**); thermal ellipsoids are shown at the 50% probability level; hydrogen atoms, solvent molecules and disorder are omitted for clarity. Selected bond lengths [Å] and angles [deg]: P1–P2 2.1966(2), P2–P3 2.1451(2), P3–P4 2.1789(2), Co1–P2 2.3297(1), Co1–P3 2.3210(1), Co1–P4 2.2935(1), Co1–S1 2.3416(1), Co1–N1 1.970(4), Co1–N2 1.986(3), P1–N3 1.670(4), P4–N3 1.782(4), P1–N4 1.666(4), P1–S1 2.0261(2), P1–P2–P3 100.50(6), P2–P3–P4 94.04(6), P2–P1–N4 120.79(1), P1–N3–P4 106.3(2), P1–N4–Si3 120.1(2), Si3–N4–Si2 120.8(2). (b) Experimental (upward) and simulated (downward) ^31^P{^1^H} NMR spectra of **5** with nuclei assigned to an AMXY spin system: δ(P_A_) = 134.5 ppm, δ(P_M_) = 49.3 ppm, δ(P_X_) = −51.8 ppm, δ(P_Y_) = −59.9 ppm, ^1^*J*_XY_ = −423 Hz, ^1^*J*_MY_ = −425 Hz, ^1^*J*_AX_ = −350 Hz, *J*_MX_ = 31 Hz, *J*_AY_ = 11 Hz, *J*_AM_ = −21 Hz.

Given the ionic nature of **4**^–^ and the anticipated nucleophilicity of the phosphaimino nitrogen N4 (Mulliken charge −0.48), we hypothesized that it would readily undergo salt metathesis reactions with electrophiles. Our assumption was confirmed when the addition of Me_3_SiCl to a solution of [K(18c-6)]**4** in toluene resulted in an immediate color change from blue to purple due to the formation of [(Ar*BIAN)Co(η^3^:η^1^-P_4_SN_2_(SiMe_3_)_3_)] (**5**, Scheme 2b), which was crystallized as purple needles from *n*-hexane at −35 °C in 63% isolated yield. Synchrotron SCXRD analysis of **5** revealed the silylation of the imino moiety, resulting in a bis(trimethylsilyl)amino group (Figure 4a).^12^ The structural characteristics of **5** closely resemble those of its precursor **4**^–^ (vide supra), including the presence of a central η^3^-coordinating azatetraphosphole ring. However, the P1–N4 bond length (1.666(4) Å) is elongated due to its increased single bond character. The silylated nitrogen atom N4 in **5** adopts an almost trigonal planar geometry (∑∠ 358°) positioned 0.144(4) Å above the plane defined by Si2, Si3, and the chiral phosphorus atom P1. The presence of the trimethylsilyl groups attached to N4 is reflected in the ^1^H NMR spectrum by two distinct signals, which persist even when the sample is subjected to variable temperature (VT) NMR experiments at up to 100 °C (Figure S11, SI).

This phenomenon is attributed to restricted rotation around the P1–N4 bond, which frustrates chemical equivalence of the trimethylsilyl groups on the NMR time scale. In addition, the three inequivalent silicon atoms are discernible in the ^29^Si{^1^H} NMR spectrum, giving rise to two doublets at δ = 7.4 (Si3, ^2^*J*_SiP_ = 11 Hz) and δ = 11.6 ppm (Si2, ^2^*J*_SiP_ = 6 Hz), as well as a singlet at δ = 9.3 ppm for Si1. The assignment of the signals has been facilitated through both homo- and heteronuclear 2D NMR spectroscopy, further supported by the observed ^2^*J*_SiP_ coupling constants (vide supra). The P_4_ unit in **5** gives rise to an AMXY spin system in the ^31^P{^1^H} NMR spectrum (Figure 4b), featuring chemical shifts and coupling constants akin to those of the precursor **4**^–^ and related asymmetric P_4_-chains.^7,8b,15^ In contrast, the symmetrical, uncoordinated, and cyclic azaphosphane [(PPh)_4_NMe] exhibits two multiplet resonances at δ = 126.0 ppm and δ = 13.2 ppm, which are distinct from the resonances of compound **5**.^13h^ In particular, the resonances of the middle phosphorus atoms in the chain of **5** are observed at higher field at δ = −51.8 ppm and δ = −59.9 ppm.

The addition of the SiMe_3_ group is a reversible process, as treatment of **5** with either cyanide or alkoxide salts regenerate anion **4**^–^. These feature either [*n*Bu_4_N]^+^ or [K(18c-6)]^+^ cations, depending on the salt used (Scheme 2c), resembling classic acid–base reactivity (Figure S27).

**Scheme 3:**
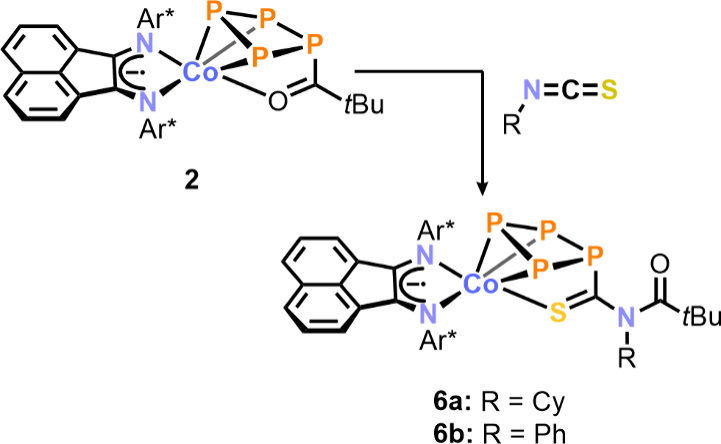
Insertion of Isothiocyanates into the P–C Bond of **2** Reagents and conditions: 1.4 equiv of CyNCS; toluene, r.t., 3 d (**6a**); 1.1 equiv of PhNCS; toluene, r.t., 3 h (**6b**); yields **6a**: 80% **6b**: 63%.

Shifting our focus from anionic *cyclo*-P_4_ complex **1**^–^, we investigated the acylated and neutral [(Ar*BIAN)Co(η^3^:η^1^-P_4_C(O)*t*Bu)] (**2**), anticipating it might exhibit similar reactivity toward electrophilic heterocumulenes. However, likely due to the reduced nucleophilicity of the acylated phosphorus atoms in **2**, no significant reactivity was observed with either CS_2_ or S(NR)_2_. Nonetheless, the addition of sulfur-containing isothiocyanates, specifically CyNCS or PhNCS, to a solution of **2** resulted in a notable color change from magenta to purple (Scheme 3). The reaction with PhNCS (1.1 equiv) led to the complete conversion of **2** within 3 h, according to ^31^P{^1^H} NMR spectroscopic monitoring. In contrast, the reaction with CyNCS (1.4 equiv) proceeded at a markedly slower pace and achieved full conversion after 3 days. We propose a reaction mechanism for the isothiocyanate insertion that begins with the attack of the acylated phosphorus atom on the carbon atom of the heterocumulene. This is followed by attack of the nitrogen on the carbonyl carbon atom and finally the coordination of the sulfur atom to the cobalt center (see the SI, Scheme S2). The resulting complexes [(Ar*BIAN)Co(η^3^:η^1^-P_4_C(S)N(R)C(O)*t*Bu)] (R = Cy (**6a**); R = Ph (**6b**)) were isolated in 80% and 64% yield, respectively.

**Figure 5:**
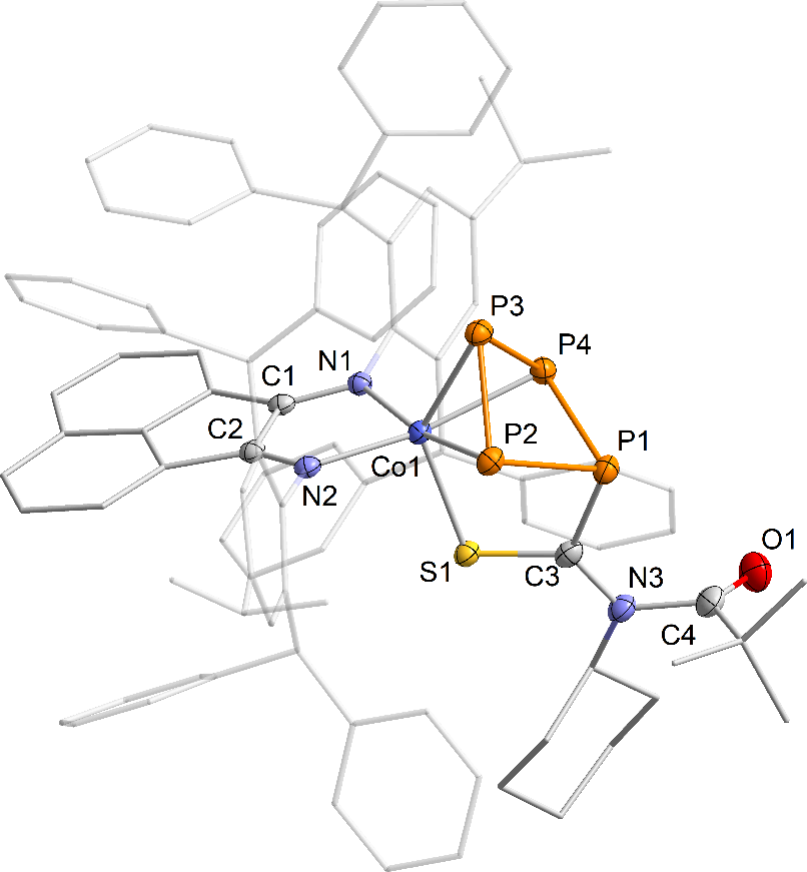
Solid-state molecular structure of [(Ar*BIAN)Co(η^3^:η^1^-P_4_C(S)N(Cy)C(O)*t*Bu)] (**6a**); thermal ellipsoids are shown at the 50% probability level; hydrogen atoms and solvent molecules are omitted for clarity. Selected bond lengths [Å] and angles [deg]: P1–P2 2.2437(8), P2–P3 2.1697(7), P3–P4 2.1669(8), P1–P4 2.2360(7), Co1–P2 2.2881(5), Co1–P3 2.2915(6), Co1–P4 2.2838(8), Co1–S1 2.2583(6), Co1–N1 1.9701(2), Co1–N2 1.9693(2), S1–C3 1.696(2), C3–N3 1.343(3), C4–N3 1.471(3), P1–C3 1.856(2), C4–O1 1.196(3), P1–P2–P3 88.80(3), P2–P3–P4 85.38(3), P3–P4–P1 89.07(3), P4–P1–P2 82.05(3).

Single-crystal XRD analysis performed on large block-shaped crystals, grown from toluene, confirmed the insertion of the isothiocyanate into the P–C bond of **2**, forming **6a** (Figure 5). While there are documented instances of isothiocyanates undergoing insertion into P–P, P–Si, and P–H bonds, to our knowledge this marks the first example of such a reaction involving a P–C bond.^5,17^ In **6a**, the thioacyl group coordinates to the cobalt via the sulfur atom, rather than through the oxygen atom of the remote acyl group.^7^ This coordination shift is reflected in the ATR-IR spectrum, where the C=O stretching vibration in **6a** was distinctly observed at ν̃_CO_ = 1727 cm^–1^, a band typical for acyl groups.^18^ This contrasts the C=O stretch in **2**, which was predicted to occur at ν̃_CO_ = 1462 cm^–1^, thereby overlapping with the BIAN C–N vibrations in the fingerprint region.^7^ The puckered *cyclo*-P_4_ moiety observed in complex **6a** closely resembles that in complexes **2**, **3**^–^ and [Cp‴Co(η^3^-P_4_R_2_)] (Cp‴ = C_5_H_2_*t*Bu_3_; R = Ph, Cy, *t*Bu),^7,11^ featuring elongated P1–P2 (2.2437(8) Å) and P1–P4 (2.2360(7) Å) bond lengths alongside shorter P2–P3 (2.1697(7) Å) and P3–P4 (2.1669(8) Å) bond lengths, indicative of some degree of multiple bond character. Furthermore, the bond lengths of S1–C3 (1.696(2) Å) and C3–N3 (1.343(3) Å) are elongated relative to those in free aryl isothiocyanate (S–C, 1.566 Å; C–N 1.152 Å), indicating increased single bond character in **6a**.^19^ The coordination sphere of the cobalt center is completed by an Ar*BIAN^•–^ radical anion.^20^ The phenyl-substituted derivative is essentially isostructural with **6b** (Figure S41, SI).

**Figure 6:**
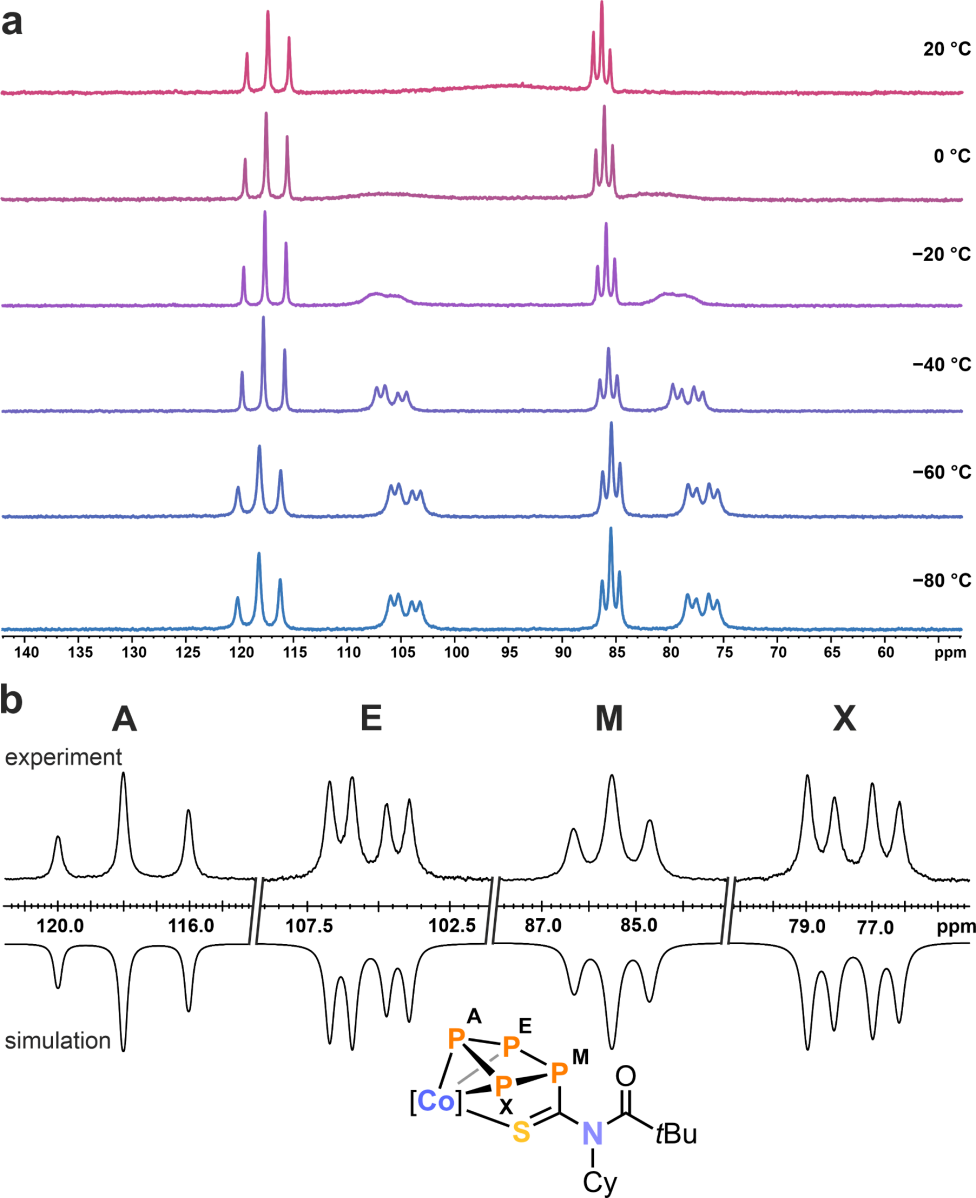
(a) Variable temperature ^31^P{^1^H} NMR spectra of **6a** in toluene-*d*_8_. (b) Experimental (upward) and simulated (downward) ^31^P{^1^H} NMR spectra of **6a** at −60 °C in toluene-*d*_8_ with nuclei assigned to an AEMX spin system: δ(P_A_) = 117.9 ppm, δ(P_E_) = 105.4 ppm, δ(P_M_) = 85.5 ppm, δ(P_X_) = 77.6 ppm, ^1^*J*_AE_ = −324 Hz, ^1^*J*_AX_ = −321, ^1^*J*_EM_ = −129 Hz, ^1^*J*_MX_ = −133 Hz, ^2^*J*_AM_ = 18 Hz, ^2^*J*_EX_ = 20 Hz.

The ^31^P{^1^H} NMR spectrum of **6a** in C_6_D_6_ features two triplets at δ = 86.3 (P_M_) ppm and δ = 117.3 (P_A_) ppm, as well as a significantly broadened signal (Δν_1/2_ = 2500 Hz) at δ = 93.0 (P_E/X_) ppm. This broadening suggests a dynamic process occurring in solution. Given the solid-state molecular structure of **6a**, two distinct signals are expected for the phosphorus atoms P2 and P4 if the rotation is restricted around the C4–N3 or the C3–N3 axis, with the latter axis exhibiting partial multiple bond character (1.343(3) Å vs ∑*r*_CN_ 1.46 Å for a single bond; labeling according to Figure 5). VT ^31^P{^1^H} NMR spectroscopy elucidated this phenomenon further, revealing that the broad resonance at ambient temperature separates into two distinct signals at 0 °C. These resolve below −40 °C into distinct multiplets, indicative of an AEMX spin system (Figure 6). In contrast, the ^31^P{^1^H} NMR spectrum of **6b**, which possesses nearly identical C3–N3 and C4–N3 bond lengths, displays well-resolved signals conforming to an AB_2_X spin system with similar chemical shifts akin to those of **6a** (see Figure S23, SI). This distinct behavior in solution is probably due to hindered rotation resulting from the steric demand of the substituent.

## Conclusion

The reaction of anionic *cyclo*-P_4_ complex **1**^–^ with CS_2_ leads to the electrophilic addition of the heterocumulene to the *cyclo*-P_4_ ligand, resulting in the formation of **3**^–^, which features a puckered η^3^:η^1^-P_4_CS_2_ ligand. Initial reactivity studies of **3**^–^ toward electrophiles indicate a propensity for salt metathesis reactions, suggesting new pathways for subsequent functionalization. Upon employing the sulfur diimide S(NSiMe_3_)_2_ as the reactant, P–P bond insertion was facilitated for *cyclo*-P_4_ complex **1**^–^, yielding complex **4**^–^, with a novel CoP_4_N^–^ core. Compound **3**^–^ represents the first azatetraphosphole complex and undergoes further functionalization to yield **5** upon reaction with Me_3_SiCl. These compounds, **4**^–^ and **5**, have been characterized with various analytical techniques, including single crystal X-ray structural analysis at synchrotron facilities and computational chemistry studies. The neutral complex **2** exhibits discrepant reactivity, undergoing insertion of isothiocyanates into the P–C bond of the acylated tetraphosphido ligand, yielding the highly derivatized complexes **6a** and **6b**. This new reaction type expands the repertoire of P–C bond insertion reactions available for the strategic functionalization of tetraphosphido ligands.

Overall, our findings highlight the versatility and potential of low-valent polyphosphido complexes for effecting targeted and diverse transformations of P_4_. With increased availability of routes to various *cyclo*-P_4_ complexes, particularly highlighted by recent advancements, this paves the way to unique phosphorus compounds. Ongoing research in this area is instrumental in deepening our understanding of reactivity patterns and mechanisms, laying the essential groundwork for the development of systems capable of facilitating the efficient transition-metal-mediated functionalization of P_4_.

## Experimental Section

### General Considerations

All experiments were performed under an atmosphere of dry argon, by using standard Schlenk and glovebox techniques. Solvents were purified, dried, and degassed with an MBraun SPS800 solvent purification system. All dry solvents except *n*-hexane were stored under argon over activated 3 Å molecular sieves in gastight ampules. *n*-Hexane was stored over a potassium mirror. NMR spectra were recorded on Bruker Avance 400 spectrometers. ^1^H and ^13^C{^1^H} spectra were referenced internally to residual solvent resonances. ^31^P{^1^H} spectra were referenced externally to 85% H_3_PO_4_ (aq). The assignment of the ^1^H and ^13^C NMR signals was confirmed by two-dimensional (COSY, HSQC, HMBC, NOESY and ROESY) experiments. A more detailed assignment of the signals can be found in the SI. For compounds which give rise to a higher order spin system in the ^31^P{^1^H} NMR spectrum, the resolution enhanced ^31^P{^1^H} NMR spectrum was transferred to the software gNMR, version 5.0.6, by Cherwell Scientific.^21^ The full line shape iteration procedure of gNMR was applied to obtain the best match of the fitted to the experimental spectrum. ^1^*J*(^31^P^31^P) coupling constants were set to negative values and all other signs of the coupling constants were obtained accordingly.^22^

UV/vis spectra were recorded on an Ocean Optics Flame Spectrometer with a DH-2000-BAL light source. Elemental analyses were performed by the Central Analytics Department of the University of Regensburg using a Vario micro cube. Mass spectra were recorded on a Finnigan MAT 95 spectrometer. IR spectra were recorded with a Bruker ALPHA spectrometer equipped with a diamond ATR unit.

S(NSiMe_3_)_2_ and CS_2_ (*c* = 5.0 M in THF) were purchased from Sigma-Aldrich; PhNCS, CyNCS from Alfa Aesar; and all were used as received. Trimethylsilyl chloride was purchased from Sigma-Aldrich and a stock solution (*c* = 1.58 M in toluene) was prepared. The starting materials [K(18c-6)][(Ar*BIAN)Co(η^4^-P_4_)] ([K(18c-6)]**1**) and [(Ar*BIAN)Co(η^3^:η^1^-P_4_C(O)*t*Bu)] (**2**) were prepared according to previously reported procedures.^7^

#### [K(18c-6)][(Ar*BIAN)Co(η^3^:η^1^-P_4_CS_2_)] ([K(18c-6)]**3**)

A stock solution of CS_2_ (30.6 μL, *c* = 5.0 M in THF, 0.153 mmol, 1.2 equiv) was added to a deep purple solution of [K(18c-6)]**1** (200 mg, 0.128 mmol, 1.0 equiv) in THF (4 mL) at room temperature. The reaction mixture was stirred overnight, resulting in a blue solution which was filtered. The filtrate was layered with *n*-hexane (12 mL). After 3 days, blue shimmering crystals had formed, which were isolated by decantation of the mother liquor, washed with *n*-hexane (2 × 1 mL), and dried in vacuo. The solid contained 0.4 equiv of *n*-hexane and 0.7 equiv of THF after drying as indicated by ^1^H/^13^C{^1^H} NMR spectra and elemental analysis. Yield: 186 mg (0.115 mmol, 89%). UV/vis: (THF, λ_max_/nm, ε_max_/L·mol^–1^·cm^–1^): 330 (22 000), 375sh (14 000), 570 (15 000), 725 (10500). ^1^H NMR (400.13 MHz, 300 K, THF-*d*_8_): δ/ppm = 1.11–1.16 (m, 12H), 2.78 (sept., ^3^*J*_HH_ = 6.9 Hz, 2H), 3.45 (br s, 24H), 5.06 (s, 2H), 5.50 (d, ^3^*J*_HH_ = 7.1 Hz, 2H), 6.22–6.26 (m, 2H), 6.41–6.46 (m, 8H), 6.51–6.59 (m, 4H), 6.65–6.70 (m, 4H), 6.79–6.81 (m, 4H), 6.85–6.86 (m, 2H), 6.90–7.10 (m, 14H), 7.24 (d, ^3^*J*_HH_ = 8.2 Hz, 2H), 7.31–7.33 (m, 4H), 7.57–7.59 (m, 4H), 7.96 (s, 2H). ^13^C{^1^H} NMR (100.66 MHz, 300 K, THF-*d*_8_): δ/ppm = 23.9 (s), 24.3 (s), 34.2 (s), 51.1 (s), 52.5 (s), 71.1 (s), 120.7 (*s*), 122.9 (s), 125.3 (s), 125.6 (s), 125.7 (s), 125.9 (s), 127.4 (s), 127.5 (s), 127.6 (s), 127.7 (s), 128.0 (s), 128.2 (s), 130.6 (s), 130.8 (s), 130.9 (s), 131.2 (s), 131.5 (s), 134.0 (s), 134.1 (s), 134.4 (s), 138.8 (s), 143.6 (s), 143.9 (s), 145.0 (s), 146.6 (s), 147.6 (s), 150.9 (s), 159.8 (s); *C*=S: not detected. ^31^P{^1^H} NMR (162.04 MHz, 300 K, THF-*d*_8_): δ/ppm = 83.3–86.1 (m, 2P, P_Y_), 97.8–101.7 (m, 1P, P_X_), 127.6 (t, 1P, P_A_), for parameters obtained by simulation, see Figure S3 and Table S1, SI. Anal. Calcd (%) for (C_95_H_92_CoKN_2_O_6_P_4_S_2_)·(*n*-hexane)_0.4_(THF)_0.7_ (*M*_w_ = 1643.84 g·mol^–1^) C 69.62, H 6.02, N 1.62, S 3.71; found C 69.25, H 6.07, N 1.48, S 4.11.

#### [K(18c-6)][(Ar*BIAN)Co(η^3^:η^1^-P_4_SN_2_(SiMe_3_)_2_)] [K(18c-6)]**4**)

*N*,*N*-Bis(trimethylsilyl)sulfurdiimide (19.8 mg, 22.6 μL, 0.096 mmol, 1.5 equiv) was added to a deep purple solution of [K(18c-6)]**1** (100 mg, 0.064 mmol, 1.0 equiv) in THF (2 mL). The reaction mixture was stirred at 35 °C for 6 days, resulting in a blue solution which was filtered. *n*-Hexane (40 mL) was added while stirring, precipitating a purple solid, which was isolated by filtration, washed with *n*-hexane (3 × 2 mL), and dried in vacuo. Yield: 71 mg (0.040 mmol, 63%). UV/vis: (THF, λ_max_/nm, ε_max_/L·mol^–1^·cm^–1^): 320sh (17 000), 550 (10 000), 710 (9000). ^1^H NMR (400.30 MHz, 300 K, MeCN-*d*_3_): δ/ppm = −0.14 (s, 9H), 0.09 (s, 9H), 1.11–1.17 (m, 12H), 2.76–2.88 (m, 2H), 3.55 (s, 24H), 4.66 (s, 1H), 4.98 (d, ^3^*J*_HH_ = 7.1 Hz, 1H), 5.19 (s, 1H), 5.71 (d, ^3^*J*_HH_ = 7.1 Hz, 1H), 6.06–6.19 (m, 7H), 6.32–6.36 (m, 2H), 6.47–6.59 (m, 7H), 6.73–6.77 (m, 2H), 6.81–6.98 (m, 11H), 7.03–7.32 (m, 16H), 7.43–7.48 (m, 3H), 7.95 (s, 1H), 8.49 (s, 1H). ^13^C{^1^H} NMR (100.61 MHz, 300 K, MeCN-*d*_3_): δ/ppm = 1.5 (d, ^3^*J*_CP_ = 3.9 Hz), 4.7 (d, ^3^*J*_CP_ = 3.1 Hz), 24.2 (s), 24.4 (s), 24.4 (s), 24.6 (s), 34.3 (s), 34.4 (s), 50.9 (s), 51.3 (s), 51.6 (s), 52.0 (s), 71.0 (s), 120.5 (s), 120.7 (s), 123.0 (s), 123.3 (s), 125.5 (s), 126.0 (s), 126.1 (s), 126.1 (s), 126.2 (s), 126.2 (s), 126.6 (s), 127.5 (s), 127.7 (s), 127.9 (s), 128.1 (s), 128.1 (s), 128.1 (s), 128.2 (s), 128.3 (s), 128.4 (s), 128.7 (s), 128.7 (s), 128.8 (s), 129.3 (s), 130.7 (s), 131.2 (s), 131.2 (s,), 131.3 (s), 131.4 (s), 131.4 (s), 131.6 (s), 131.6 (s), 131.7 (s), 132.9 (s), 134.2 (s), 134.6 (s), 134.7 (s), 134.9 (s), 137.9 (s), 139.3 (s), 143.1 (s), 144.1 (s), 144.4 (s), 144.5 (s), 144.6 (s), 146.7 (s), 147.9 (s), 148.5 (s), 150.0 (s), 153.8 (s), 155.4 (s), 159.9 (s), 161.6 (s). ^31^P{^1^H} NMR (161.98 MHz, 300 K, MeCN-*d*_3_): δ/ppm = −43.2 (dd, 1P, P_Y_), – 12.4 (ddd, 1P, P_X_), 29.2 (ddd, 1P, P_M_), 118.8 (dd, 1P, P_A_), for parameters obtained by simulation, see Figure S7 and Table S2, SI. ^29^Si{^1^H} NMR (79.49 MHz, 300 K, MeCN-*d*_3_): δ/ppm = −17.9 (d, ^2^*J*_SiP_ = 16.6 Hz), 3.6 (d, ^2^*J*_SiP_ = 6.1 Hz). Anal. Calcd (%) for (C_100_H_110_CoKN_4_O_6_P_4_SSi_2_) (Mw = 1774.15 g·mol^–1^) C 67.70, H 6.25, N 3.16, S 1.81; found C 67.29, H 6.29, N 3.04, S 1.72. TOF-MS (ESI, MeCN): *m*/*z*(%) calcd. for C_88_H_86_CoN_4_P_4_SSi_2_^–^ [M^–^]: 1470.4424; found 1470.4298.

#### [(Ar*BIAN)Co(η^3^:η^1^-P_4_SN_2_(SiMe_3_)_3_)] (**5**)

A stock solution of Me_3_SiCl (53.5 μL, 1.58 M in toluene, 0.085 mmol, 1.0 equiv) was added to a blue solution of [K(18c-6)]**4** (150 mg, 0.085 mmol, 1.0 equiv) in toluene (3.5 mL). The reaction mixture was stirred for 3 h, over which the color changed to purple. The suspension was filtered through a pad of silica (0.5 × 1 cm) and washed with toluene (3 × 1 mL). The solvent was removed and the purple residue extracted with *n*-hexane (8 mL). The filtrate was concentrated to half of the original volume. Storage for 1 day at room temperature and 1 day at −35 °C gave numerous shimmering purple crystals, which were isolated by decantation of the mother liquor and dried in vacuo. The crystalline solid contained 0.1 equiv of *n*-hexane and 0.1 equiv of toluene after drying as indicated by ^1^H/^13^C{^1^H} NMR spectra and elemental analysis. Yield: 82 mg (0.053 mmol, 63%). UV/vis: (THF, λ_max_/nm, ε_max_/L·mol^–1^·cm^–1^): 330sh (17 000), 550 (11 000), 700 (14 000). ^1^H NMR (400.13 MHz, 300 K, C_6_D_6_): δ/ppm = 0.05 (s, 9H), 0.20 (s, 9H), 0.38 (s, 9H), 1.09–1.14 (m, 12H), 2.58–2.71 (m, 2H), 5.40 (s, 1H), 5.43 (s, 1H), 5.55 (d, ^3^*J*_HH_ = 7.1 Hz, 1H), 6.02 (d, ^3^*J*_HH_ = 7.1 Hz, 1H), 6.17–6.21 (m, 1H), 6.29–6.33 (m, 1H), 6.49–6.56 (m, 4H), 6.62–6.76 (m, 10H), 6.92–6.94 (m, 2H), 7.03–7.21 (m, 10H, d (^3^*J*_HH_ = 8.1 Hz) overlapping with d (^3^*J*_HH_ = 8.2 Hz)), 7.27–7.34 (m, 8H), 7.37–7.37 (m, 1H), 7.41–7.44 (m, 7H), 7.46 (s, 1H), 7.51–7.53 (m, 2H), 7.72–7.74 (m, 2H), 7.97 (s). ^13^C{^1^H} NMR (100.61 MHz, 300 K, C_6_D_6_): δ/ppm = 2.8 (d, ^3^*J*_PC_ = 8.1 Hz), 5.2 (dd, ^3^*J*_PC_ = 5.9 Hz, 3.5 H), 5.7 (d, ^3^*J*_PC_ = 1.8 Hz), 24.0 (s), 24.0 (s), 24.1 (s), 24.1 (s), 33.8 (s), 33.9 (s), 51.1 (s), 51.7 (s), 52.0 (s), 52.4 (s), 121.8 (s), 122.0 (s), 124.4 (s), 124.4 (s), 125.7 (s), 125.9 (*s*), 126.0 (*s*), 126.1 (*s*), 126.2 (*s*), 127.0 (*s*), 127.7 (s), 127.7 (s), 127.8 (s), 127.9 (s), 128.0 (s), 128.1 (s), 128.2 (s), 128.3 (s), 128.5 (s), 128.6 (s), 130.2 (s), 130.5 (s), 130.7 (s), 130.8 (s), 130.8 (s), 131.0 (s), 131.0 (s), 131.3 (s), 131.4 (s), 132.3 (s), 132.4 (s), 132.9 (s), 135.2 (s), 136.5 (s), 137.9 (s), 138.8 (s), 142.8 (s), 143.1 (s), 143.4 (s), 143.8 (s), 145.3 (s), 145.5 (s), 146.1 (s), 146.5 (s), 147.8 (s), 148.1 (s), 150.9 (s), 152.2 (s), 163.1 (s), 164.2 (s). ^31^P{^1^H} NMR (162.04 MHz, 300 K, C_6_D_6_): (AMXY) spin system δ/ppm = −60.1 (dd, 1P, P_Y_), –54.2 - –49.0 (m, 1P, P_X_), 47.9–50.8 (m, 1P, P_M_), 133.4–135.7 (m, 1P, P_A_), for parameters obtained by simulation, see Figure S13 and Table S3. ^29^Si{^1^H} NMR (79.49 MHz, 300 K, C_6_D_6_): δ/ppm = 7.4 (d, ^2^*J*_SiP_ = 10.9 Hz), 9.3 (s), 11.6 (d, ^2^*J*_SiP_ = 6.1 Hz). Anal. Calcd (%) for (C_91_H_95_CoN_4_P_4_SSi_3_)·(toluene)_0.1_·(*n*-hexane)_0.1_ (*M*_w_ = 1543.93 g·mol^–1^) C 70.98, H 6.27, N 3.59, S 2.05; found C 71.33, H 5.88, N 3.51, S 2.07.

#### [(Ar*BIAN)Co(η^3^:η^1^-P_4_C(S)N(Cy)C(O)*t*Bu)] (**6a**)

Neat cyclohexyl isothiocyanate (7.3 mg, 7.4 μL, 0.052 mmol, 1.4 equiv) was added to a magenta-colored solution of **2** (50 mg, 0.037 mmol, 1.0 equiv) in toluene (1.5 mL). The reaction mixture was stirred for 3 days, giving a purple solution. The solvent was removed in vacuo. Subsequently, the resulting purple residue was washed with *n*-hexane (3 × 0.5 mL) and dried in vacuo yielding a deep purple powder. Complex **6a** is stable in solution even when heated to 50 °C for up to 3 weeks. Yield: 44 mg (0.030 mmol, 80%). UV/vis: (toluene, λ_max_/nm, ε_max_/L·mol^–1^·cm^–1^): 330 (12 000), 430 (2500), 550 (5000), 720 (8000). ^1^H NMR (400.13 MHz, 300 K, C_6_D_6_): δ/ppm = 0.60–0.65 (m, 5H), 0.92 (s, 9H), 1.02–1.05 (m, 12H), 1.16–1.22 (m, 3H), 1.46–1.49 (m, 2H), 2.58 (sept., ^3^*J*_HH_ = 6.9 Hz, 2H), 3.43–3.48 (m, 1H), 5.50 (s, 2H), 5.89 (d, ^3^*J*_HH_ = 7.1 Hz, 2H), 6.22–6.26 (m, 2H), 6.64–6.65 (m, 6H), 6.71–6.75 (m, 2H), 6.82–6.98 (m, 10H), 7.06–7.15 (m, 14H), 7.19 (d, ^3^*J*_HH_ = 8.2 Hz, 2H), 7.29–7.29 (m, 2H), 7.36–7.37 (m, 2H), 7.35–7.36 (m, 2H), 7.62–7.67 (m, 8H), 7.92 (s, 2H). ^13^C{^1^H} NMR (100.66 MHz, 300 K, C_6_D_6_): δ/ppm = 24.4 (s), 24.5 (s), 26.1 (s), 26.6 (s), 29.0 (s), 31.2 (s), 34.4 (s), 44.1 (s), 51.7 (s), 53.3 (s), 67.1 (s), 122.6 (s), 125.4 (s), 126.5 (s), 126.6 (s), 126.9 (s), 127.3 (s), 128.4 (s), 128.5 (s), 128.5 (s), 128.7 (s), 129.0 (s), 129.2 (s), 130.6 (s), 130.9 (s), 131.1 (s), 131.4 (s), 132.6 (s), 135.2 (s), 136.8 (s), 139.0 (s), 143.5 (s), 145.3 (s), 146.0 (s), 146.3 (s), 148.5 (s), 149.7 (s), 164.3 (s), 183.1 (s); *C*=S: not detected. ^31^P{^1^H} NMR (162.04 MHz, 300 K, C_6_D_6_): δ/ppm = 86.3 (t, 1P, P_M_), 93.0 (br s, Δν_1/2_ = 2500 Hz, 2P, P_E/X_), 117.3 (t, 1P, P_A_); (161.98 MHz, toluene-*d*_8_, 213 K): δ/ppm = 77.6 (dd, 1P, P_X_), 85.5 (t, 1P, P_M_), 105.3 (dd, 1P, P_E_), 118.0 (t, 1P, P_A_), for parameters obtained by simulation, see Figure S19 and Table S4, SI. IR (solid state): ν̃/ cm^–1^ = 3058w, 3023w, 2953w, 2928w, 1944w, 1805w, 1727m (C=O), 1600w, 1533m, 1492s, 1446m, 1417m, 1369s, 1322m, 1297m, 1255w, 1193m, 1153w, 1101w, 1076w, 1035m, 1007m, 920w, 895w, 842w, 820m, 761m, 736s, 736m, 696s, 654m, 634m, 606s. Anal. Calcd (%) for (C_94_H_88_CoN_3_OP_4_S) (*M*_w_ = 1490.65 g·mol^–1^) C 75.74, H 5.95, N 2.82, S 2.15; found: C 75.60, H 5.93, N 2.58, S 1.75.

#### [(Ar*BIAN)Co(η^3^:η^1^-P_4_C(S)N(Ph)C(O)tBu)] (**6b**)

Neat PhNCS (11.0 mg, 9.7 μL, 0.081 mmol, 1.1 equiv) was added to a magenta-colored solution of **2** (100 mg, 0.074 mmol, 1.0 equiv) in toluene (2 mL). The reaction mixture was stirred for 3 h, over which the color changed to purple. The solvent was removed in vacuo, and the purple residue extracted with *n*-hexane (30 mL). The mixture was filtered, and the filtrate concentrated until incipient crystallization. Purple crystals formed upon storage for 2 days at −35 °C. The crude product (84 mg) was isolated by decantation of the supernatant. Recrystallization from Et_2_O (2 mL) at −35 °C gave shimmering deep purple crystals, which were isolated by decantation of the mother liquor and dried in vacuo. The compound decomposes to new species (identified by ABMX and AEMX spin systems in the ^31^P{^1^H} NMR spectrum) in solution at ambient temperature over the course of hours. Yield: 69 mg (0.046 mmol, 63%). UV/vis: (toluene, λ_max_/nm, ε_max_/L·mol^–1^·cm^–1^): 325 (22500), 530 (9000), 710 (13 000). ^1^H NMR (400.13 MHz, 300 K, C_6_D_6_,): δ/ppm = 0.76 (s, 9H), 1.00–1.03 (m, 12H), 2.55 (sept., ^3^*J*_HH_ = 6.9 Hz, 2H), 5.45 (s, 2H), 5.80–5.82 (m, 2H), 5.85 (d, ^3^*J*_HH_ = 7.1 Hz), 6.21–6.25 (m, 2H), 6.57–6.82 (m, 17H), 7.01–7.16 (m, 16H), 7.25–7.29 (m, 6H), 7.31–7.34 (m, 6H), 7.57 (s, 2H), 7.74–7.76 (br m, 4H). ^13^C{^1^H} NMR (100.61 MHz, 273 K, toluene-*d*_8_): δ/ppm = 24.3 (s), 24.5 (s), 28.5 (s), 34.3 (s), 43.3 (s), 51.4 (s), 52.9 (s), 122.5 (s), 125.5 (s), 126.5 (s), 126.5 (s), 126.6 (s), 127.2 (s), 127.9 (s), 128.3 (s), 128.3 (s), 128.4 (s), 128.5 (s), 128.6 (s), 128.8 (s), 128.7 (s), 128.9 (s), 130.1 (s), 130.2 (s), 130.7 (s), 130.9 (s), 131.1 (s), 132.0 (s), 135.2 (s), 136.6 (s), 138.7 (s) 141.6 (s), 142.7 (s), 145.0 (s), 145.8 (s), 146.2 (s), 148.5 (s), 149.0 (s), 164.4 (s), 182.2 (s); *C*=S: not detected. ^31^P{^1^H} NMR (162.04 MHz, 300 K, C_6_D_6_): (AB_2_X) spin system δ/ppm = 95.5–98.8 (m, 1P, P_*x*_), 103.8–109.5 (m, 3P, P_A_/P_B_), for parameters obtained by simulation, see Figure S23 and Table S5. IR (solid state): ν̃/ cm^–1^ = 3056w, 3023w, 2956w, 2924w, 2160w, 2031w, 1735w (C=O), 1685w, 1598w, 1530w, 1492m, 1450m, 1471m, 1361w, 1296w, 1253w, 1192w, 1163w, 1075w, 1030w, 949w, 917w, 894w, 820m, 737m, 695s, 655m, 605m. Anal. Calcd (%) for (C_94_H_82_CoN_3_OP_4_S) (*M*_w_ = 1484.60 g·mol^–1^) C 76.05, H 5.57, N 2.83, S 2.16; found: C 76.34, H 5.69, N 2.82, S 1.97.

## Supporting Information Available

NMR, UV/vis, and IR spectra for all compounds, additional experiments, proposed reaction mechanism, computational details, and crystallographic refinement (PDF)
