# Supplementary material for: Functionalization of Tetraphosphido Ligands by Heterocumulenes
Source: Inorg Chem. 2024 May 31;63(43):20141–52. doi: 10.1021/acs.inorgchem.4c00808 (PMC11523228; doi:10.1021/acs.inorgchem.4c00808)
Supplement: Supplementary file 1 — ic4c00808_si_001.pdf [file ic4c00808_si_001.pdf]

Supporting Information

Functionalization of Tetraphosphido Ligands by Heterocumulenes

*Sebastian Hauer,[a] Gábor Balázs,[a] Fabian Gliese,[a] Florian Meurer,[a,b] Thomas M. Horsley Downie,[a] Christoph Hennig,[b,c] Jan. J. Weigand,[d] and Robert Wolf[a]**

[a]University of Regensburg, Institute of Inorganic Chemistry, 93040 Regensburg, Germany

[b]European Synchrotron Radiation Facility, Rossendorf Beamline (BM20-CRG), 38043 Grenoble, France

[c]Helmholtz-Zentrum Dresden-Rossendorf, Institute of Resource Ecology, 01314 Dresden, Germany

[d]Faculty of Chemistry and Food Chemistry, Technische Universität Dresden, 01062 Dresden, Germany

*Corresponding author e-mail: robert.wolf@ur.de

Table of Contents

NMR spectra 3

Additional experiments 20

UV-Vis spectra 21

IR spectra 24

X-Ray crystallography 25

Proposed reaction mechanism for insertion of isothiocyanates 31

Quantum chemical calculations 32

References 47

NMR spectra

[K(18c-6)][(Ar*BIAN)Co(η^3:η^1-P_4CS_2)] ([K(18c-6)]3)

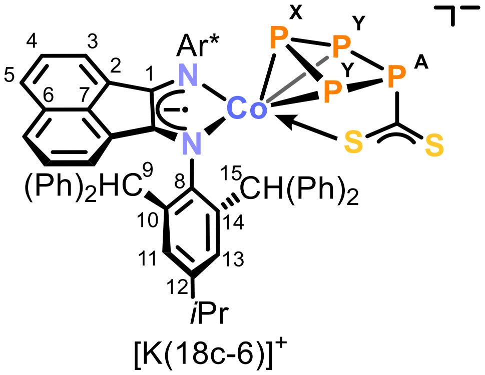

^{1}H NMR (400.13 MHz, 300 K, THF-d_8): δ/ppm = 1.11-1.16 (m, 12H, −CH(C*H*$_3$)$_2$ of *i*Pr), 2.78 (sept, $^3J_{HH}$ = 6.9 Hz, 2H, −C*H*(CH$_3$)$_2$ of *i*Pr), 3.45 (br s, 24H, 18c-6), 5.06 (s, 2H, −C^9*H*(Ph)$_2$), 5.50 (d, $^3J_{HH}$ = 7.1 Hz, 2H, C^3−*H* of BIAN), 6.22-6.26 (m, 2H, C^4−*H* of BIAN), 6.41-6.46 (m, 8H, C−*H*$_{Ar}$ of Ph), 6.51-6.59 (m, 4H, C−*H*$_{Ar}$ of Ph), 6.65-6.70 (m, 4H, C−*H*$_{Ar}$ of Ph), 6.79-6.81 (m, 4H, C−*H*$_{Ar}$ of Ph), 6.85-6.86 (m, 2H, C^{11}−*H*), 6.90-7.10 (m, 14H, C−*H*$_{Ar}$ of Ph overlapping with C^{13}−*H*), 7.23 (d, $^3J_{HH}$ = 8.2 Hz, 2H, C^5−*H* of BIAN), 7.31-7.33 (m, 4H, C−*H*$_{Ar}$ of Ph), 7.57-7.59 (m, 4H, C−*H*$_{Ar}$ of Ph), 7.96 (s, 2H, −C^{15}*H*(Ph)$_2$). **^{13}C{^{1}H} NMR** (100.66 MHz, 300 K, THF-d_8): δ/ppm = 23.9 (s, −CH(*C*H$_3$)$_2$ of *i*Pr), 24.3 (s, −CH(*C*H$_3$)$_2$ of *i*Pr), 34.2 (s, −*C*H(CH$_3$)$_2$ of *i*Pr), 51.1 (s, −*C*^{9}H(Ph)$_2$), 52.5 (s, −*C*^{15}H(Ph)$_2$), 71.1 (s, 18c-6), 120.7 (s, *C*^{3}−H of BIAN), 122.9 (s, *C*^{5}−H of BIAN), 125.3 (s, *C*$_{Ar}$−H of Ph), 125.6 (s, *C*$_{Ar}$−H of Ph), 125.7 (s, *C*$_{Ar}$−H of Ph), 125.9 (s, *C*$_{Ar}$−H of Ph), 127.4 (s, *C*^{11}−H), 127.5 (s, *C*^{13}−H), 127.6 (s, *C*$_{Ar}$−H of Ph), 127.7 (s, *C*$_{Ar}$−H of Ph), 127.7 (s, *C*$_{Ar}$−H of Ph), 128.0 (s, *C*$_{Ar}$−H of Ph), 128.2 (s, *C*^{4}−H of BIAN), 130.6 (s, *C*$_{Ar}$−H of Ph), 130.8 (s, *C*$_{Ar}$−H of Ph), 130.9 (s, *C*^{6} of BIAN), 131.2 (s, *C*$_{Ar}$−H of Ph), 131.5 (s, *C*$_{Ar}$−H of Ph), 134.0 (s, *C*^{2} of BIAN), 134.1 (s, *C*^{10}), 134.4 (s, *C*^{7} of BIAN), 138.8 (s, *C*^{14}), 143.6 (s, *C*$_{Ar}$ of Ph), 143.9 (s, *C*^{12}), 145.0 (s, *C*$_{Ar}$ of Ph), 146.6 (s, *C*$_{Ar}$ of Ph), 147.6 (s, *C*$_{Ar}$ of Ph), 150.9 (s, *C*^{8}−N), 159.8 (s, *C*^{1}=N of BIAN); *C*=S: not detected. **^{31}P{^{1}H} NMR** (162.04 MHz, 300 K, THF-d_8): (AXY$_2$) spin system δ/ppm = 83.3-86.1 (m, 2P, P$_Y$), 97.8-101.7 (m, 1P, P$_X$), 127.6 (t, 1P, P$_A$), for parameters obtained by simulation, see Figure S4 and Table S1.

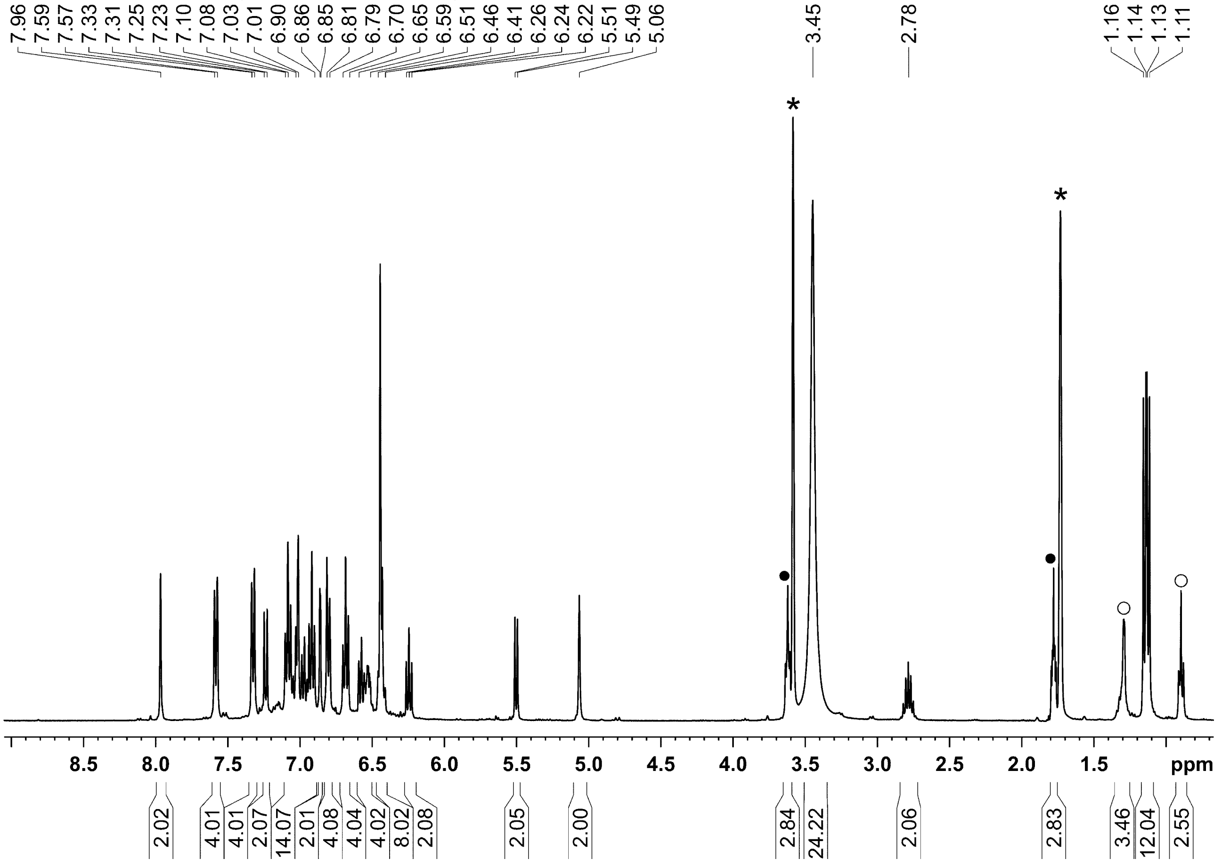

Figure S1 ^{1}H NMR spectrum (400.30 MHz, 300 K, THF-d_8) of [K(18c-6)][(Ar*BIAN)Co(η^3:η^1-P_4CS_2)] ([K(18c-6)]**3**); ○ *n*-hexane, • THF, * THF-d_8.

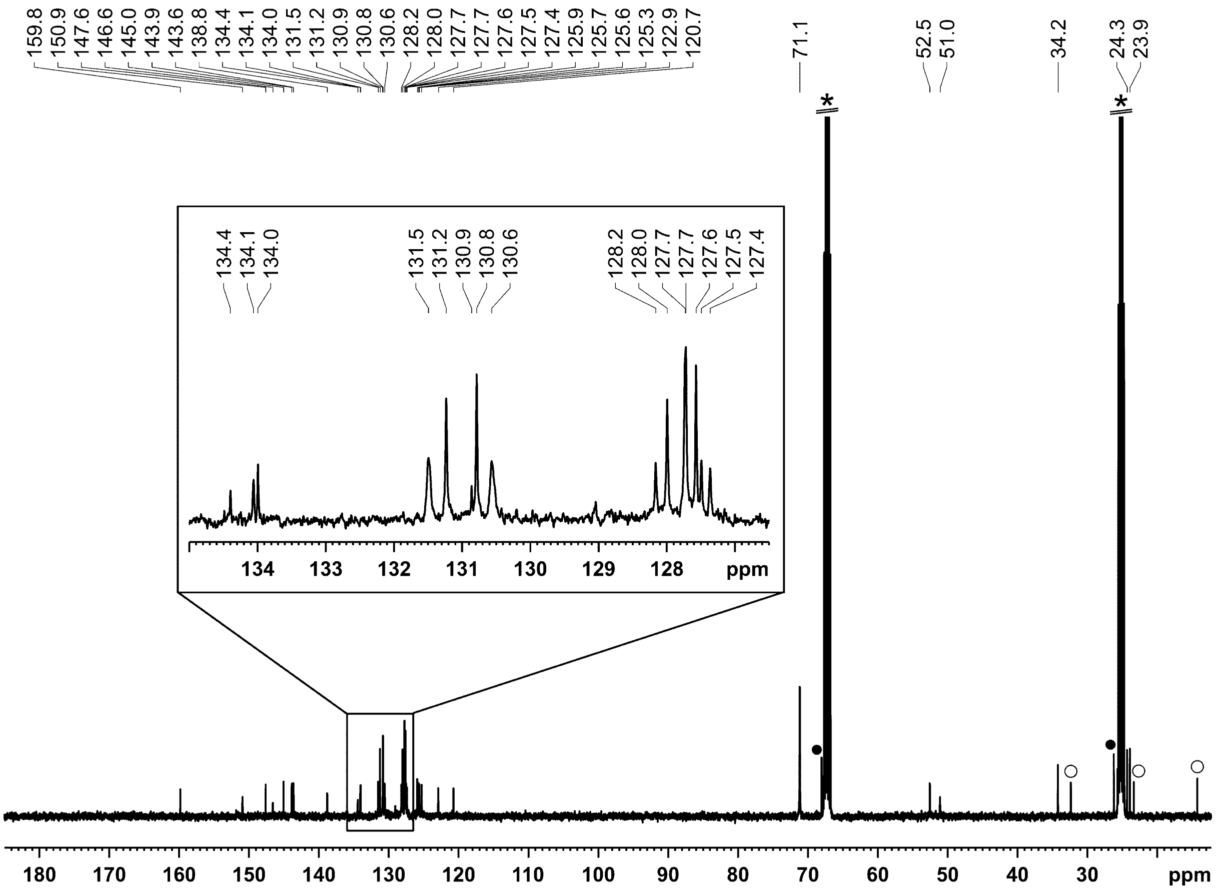

Figure S2. $^{13}C\{^{1}H\}$ NMR spectrum (100.66 MHz, 300 K, THF-d_8) of [K(18c-6)][(Ar*BIAN)Co(η^3:η^1-P_4CS_2)] ([K(18c-6)]**3**); ○ *n*-hexane, • THF, * THF-d_8.

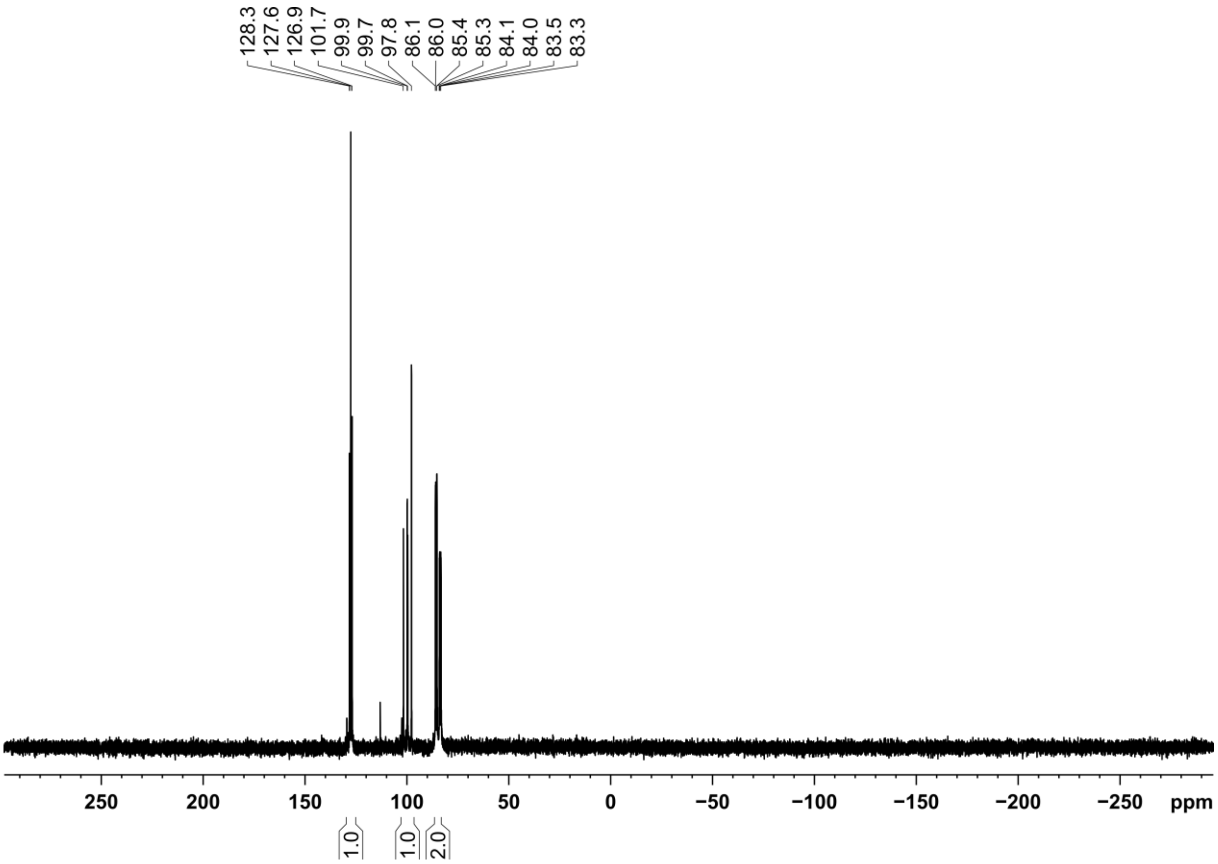

Figure S3. $^{31}P\{^{1}H\}$ NMR spectrum (162.04 MHz, 300 K, THF-d_8) of [K(18c-6)][(Ar*BIAN)Co(η^3:η^1-P_4CS_2)] ([K(18c-6)]**3**).

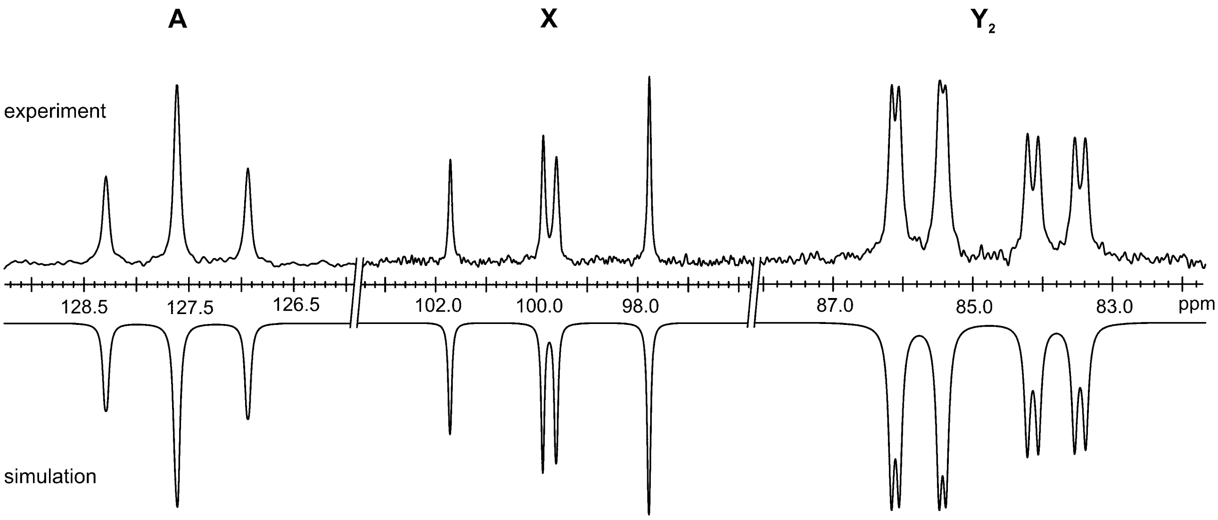

Figure S4. Section of the $^{31}P\{^{1}H\}$ NMR spectrum (162.04 MHz, 300 K, THF-d_8) of $[K(18c\text{-}6)][(Ar^{*}BIAN)Co(\eta^3{:}\eta^1\text{-}P_4CS_2)]$ ($[K(18c\text{-}6)]\mathbf{3}$); experimental (upwards) and simulation (downwards).

Table S1. Chemical shifts and coupling constants from the iterative fit of the AXY_2 spin system and schematic representation of the CoP_4CS_2 core of $[K(18c\text{-}6)][(Ar^{*}BIAN)Co(\eta^3{:}\eta^1\text{-}P_4CS_2)]$ ($[K(18c\text{-}6)]\mathbf{3}$).

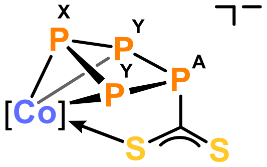	δ(A) = 127.6 ppm	$^1J_{XY}$ = –320.5 Hz
	δ(X) = 99.6 ppm	$^1J_{AY}$ = –110.1
	δ(Y) = 84.9 ppm	$^2J_{AX}$ = 5.4 Hz

$[K(18c\text{-}6)][(Ar^{*}BIAN)Co(\eta^3{:}\eta^1\text{-}P_4SN_2(SiMe_3)_2)]$ ($[K(18c\text{-}6)]\mathbf{4}$)

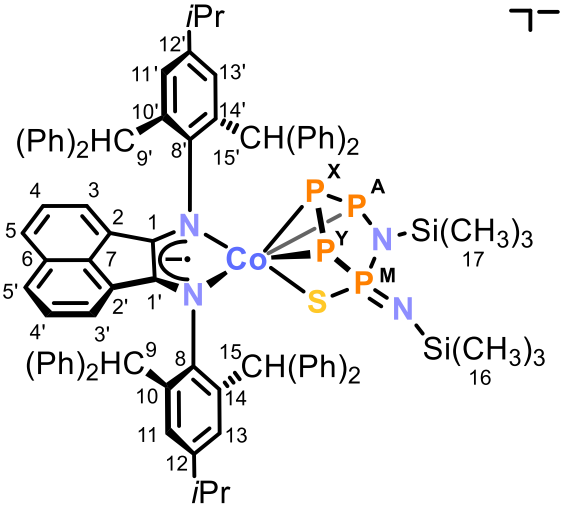

^{1}H NMR (400.30 MHz, 300 K, MeCN-d_3): δ/ppm = −0.14 (s, 9H, $-Si(C^{16}H_3)_3$), 0.06 (s, 9H, $-Si(C^{17}H_3)_3$), 1.11-1.17 (m, 12H, $-CH(CH_3)_2$ of *i*Pr), 2.76-2.88 (m, 2H, $-CH(CH_3)_2$ of *i*Pr), 3.55 (s, 24H, 18c-6), 4.66 (s, 1H, $-C^{9/9'}H(Ph)_2$), 4.98 (d, $^3J_{HH}$ = 7.1 Hz, 1H, $C^{3/3'}-H$ of BIAN), 5.19 (s, 1H, $-C^{9/9'}H(Ph)_2$), 5.71 (d, $^3J_{HH}$ = 7.1 Hz, 1H, $C^{3/3'}-H$ of BIAN), 6.06-6.19 (m, 7H, $C^{4/4'}-H$ of BIAN overlapping with $C-H_{Ar}$ of Ph), 6.32-6.36 (m, 2H, $C-H_{Ar}$ of Ph), 6.47-6.59 (m, 7H, $C^{4/4'}-H$ of BIAN overlapping with $C-H_{Ar}$ of Ph), 6.73-6.77 (m, 2H, $C-H_{Ar}$ of Ph), 6.81-6.98 (m, 11H, $C^{11/11'}-H$ overlapping with $C^{13/13'}-H$ overlapping with $C-H_{Ar}$ of Ph), 7.03-7.32 (m, 16H, $C^{5/5'}-H$ of BIAN overlapping with $C-H_{Ar}$ of Ph), 7.43-7.48 (m, 3H, $C^{5/5'}-H$ of BIAN overlapping with $C-H_{Ar}$ of Ph), 7.95 (s, 1H, $-C^{15/15'}H(Ph)_2$), 8.49 (s, 1H, $-C^{15/15'}H(Ph)_2$). **$^{13}C\{^{1}H\}$ NMR** (100.61 MHz, 300 K, MeCN-d_3): δ/ppm = 1.5 (d, $^3J_{CP}$ = 3.9 Hz, $-Si(C^{17}H_3)_3$ overlapping with MeCN-d_3 solvent signal), 4.7 (d, $^3J_{CP}$ = 3.1 Hz, $-Si(C^{16}H_3)_3$), 24.2 (s, $-CH(CH_3)_2$ of *i*Pr), 24.4 (s, $-CH(CH_3)_2$ of *i*Pr), 24.4 (s, $-CH(CH_3)_2$ of *i*Pr), 24.6 (s, $-CH(CH_3)_2$ of *i*Pr), 34.3 (s, $-CH(CH_3)_2$ of *i*Pr), 34.4 (s, $-CH(CH_3)_2$ of *i*Pr), 50.9 (s, $-C^{9/9'}H(Ph)_2$), 51.3 (s, $-C^{15/15'}H(Ph)_2$), 51.6 (s, $-C^{9/9'}H(Ph)_2$), 52.0 (s, $-C^{15/15'}H(Ph)_2$), 71.0 (s, 18c-6), 120.5 (s, $C^{3/3'}-H$ of BIAN), 120.7 (s, $C^{3/3'}-H$ of BIAN), 123.0 (s, $C^{5/5'}-H$ of BIAN), 123.3 (s, $C^{5/5'}-H$ of BIAN), 125.5 (s, $C_{Ar}-H$ of Ph), 126.0 (s, $C_{Ar}-H$ of Ph), 126.1 (s, $C_{Ar}-H$ of Ph), 126.1 (s, $C_{Ar}-H$ of Ph), 126.2 (s, $C_{Ar}-H$ of Ph), 126.2 (s, $C_{Ar}-H$ of Ph), 126.6 (s, $C_{Ar}-H$ of Ph), 127.5 (s, $C^{13/13'}-H$), 127.7 (s, $C^{13/13'}-H$), 127.9 (s, $C_{Ar}-H$ of Ph), 128.1 (s, $C^{11/11'}-H$), 128.1 (s, $C_{Ar}-H$ of Ph), 128.1 (s, $C^{11/11'}-H$), 128.2 (s, $C_{Ar}-H$ of Ph), 128.3 (s, $C_{Ar}-H$ of Ph), 128.4 (s, $C^{4/4'}-H$ of BIAN), 128.7 (s, $C_{Ar}-H$ of Ph), 128.7 (s, $C^{4/4'}-H$ of Ph), 128.8 (s,

C_{Ar}–H of Ph), 129.3 (s, C_{Ar}–H of Ph), 130.7 (s, C_{Ar}–H of Ph), 131.2 (s, C_{Ar}–H of Ph), 131.2 (s, C_{Ar}–H of Ph), 131.3 (s, C_{Ar}–H of Ph), 131.4 (s, C_{Ar}–H of Ph), 131.4 (s, C_{Ar}–H of Ph), 131.4 (s, C_{Ar}–H of Ph), 131.4 (s, C_{Ar}–H of Ph), 131.5 (s, C^{6} of BIAN), 131.6 (s, C_{Ar}–H of Ph), 131.7 (s, C_{Ar}–H of Ph), 132.9 (s, $C^{10/10'}$), 134.2 (s, $C^{10/10'}$), 134.6 (s, C^{7} of BIAN), 134.7 (s, $C^{2/2'}$ of BIAN), 134.9 (s, $C^{2/2'}$ of BIAN), 137.9 (s, $C^{14/14'}$), 139.3 (s, $C^{14/14'}$), 143.1 (s, C_{Ar} of Ph), 144.1 (s, C_{Ar} of Ph), 144.4 (s, $C^{12/12'}$), 144.5 (s, $C^{12/12'}$), 144.6 (s, C_{Ar} of Ph), 145.0 (s, C_{Ar} of Ph), 146.7 (s, C_{Ar} of Ph), 147.9 (s, C_{Ar} of Ph), 148.5 (s, C_{Ar} of Ph), 150.0 (s, C_{Ar} of Ph), 153.8 (s, $C^{8/8'}$–N), 155.4 (s, $C^{8/8'}$–N), 159.9 (s, $C^{1/1'}$=N of BIAN), 161.6 (s, $C^{1/1'}$=N of BIAN). **^{31}P{^{1}H} NMR** (161.98 MHz, 300 K, MeCN-d_3): (AMXY) spin system δ/ppm = −43.2 (dd, 1P, P_Y), −12.4 (ddd, 1P, P_X), 29.2 (ddd, 1P, P_M), 118.8 (dd, 1P, P_A), for parameters obtained by simulation, see Figure S8 and Table S2. **^{29}Si{^{1}H} NMR** (79.49 MHz, 300 K, MeCN-d_3): δ/ppm = −17.9 (d, $^{2}J_{SiP}$ = 16.6 Hz, –Si(C^{16}H$_3$)$_3$), 3.6 (d, $^{2}J_{SiP}$ = 6.1 Hz, –Si(C^{17}H$_3$)$_3$).

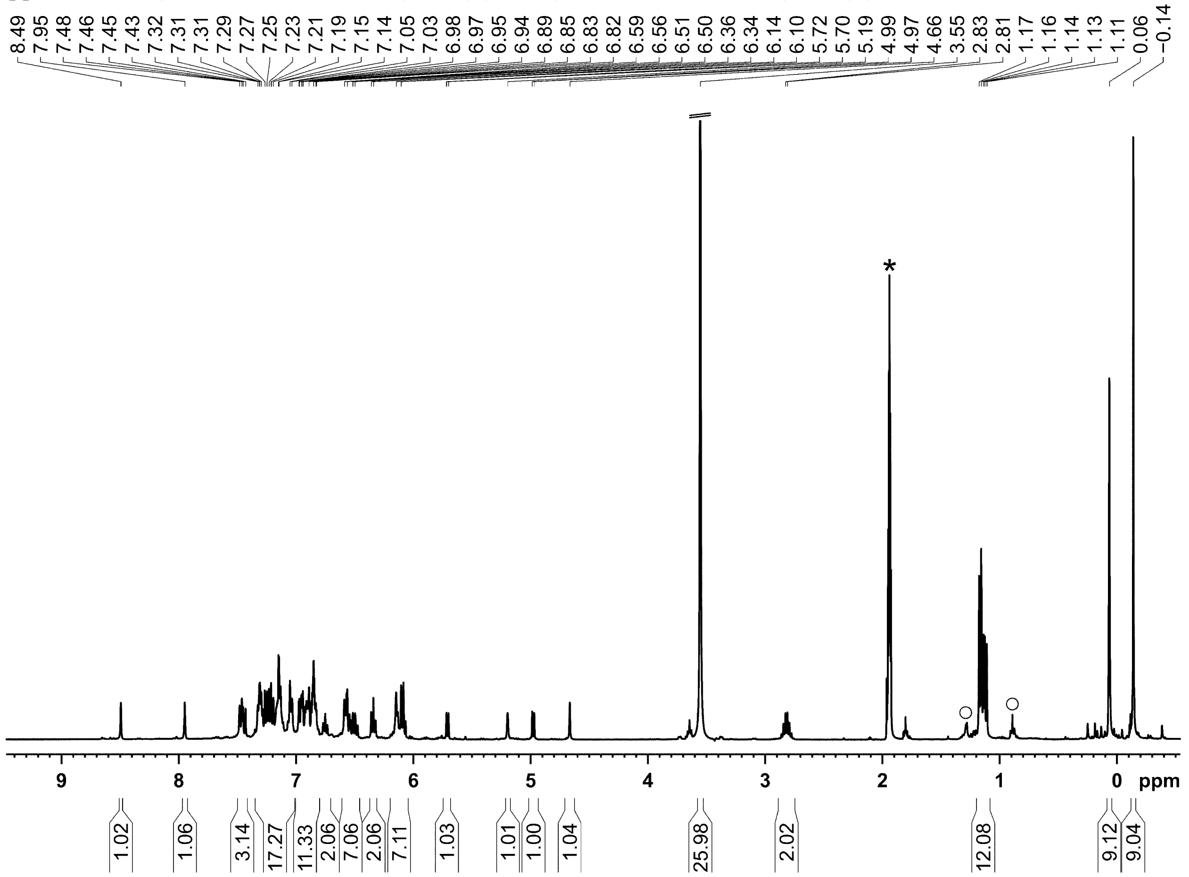

Figure S5. ^{1}H NMR spectrum (400.30 MHz, 300 K, MeCN-d_3) of [K(18c-6)][(Ar*BIAN)Co(η^3:η^1-P$_4$SN$_2$(SiMe$_3$)$_2$)] ([K(18c-6)]**4**); ○ *n*-hexane, * MeCN-d_3.

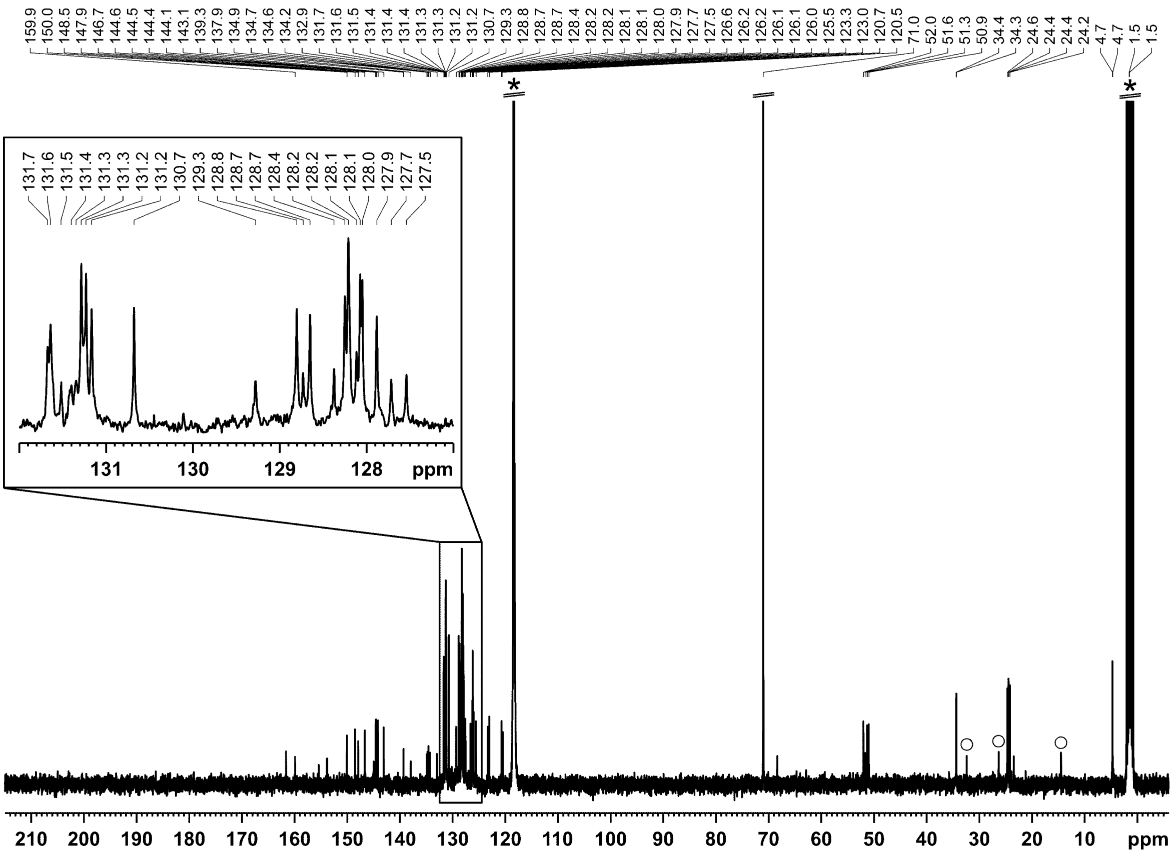

Figure S6. $^{13}C\{^{1}H\}$ NMR spectrum (100.66 MHz, 300 K, MeCN-d_3) of [K(18c-6)][(Ar*BIAN)Co(η^3:η^1-$P_4SN_2(SiMe_3)_2$)] ([K(18c-6)]**4**); ○ *n*-hexane, * MeCN-d_3.

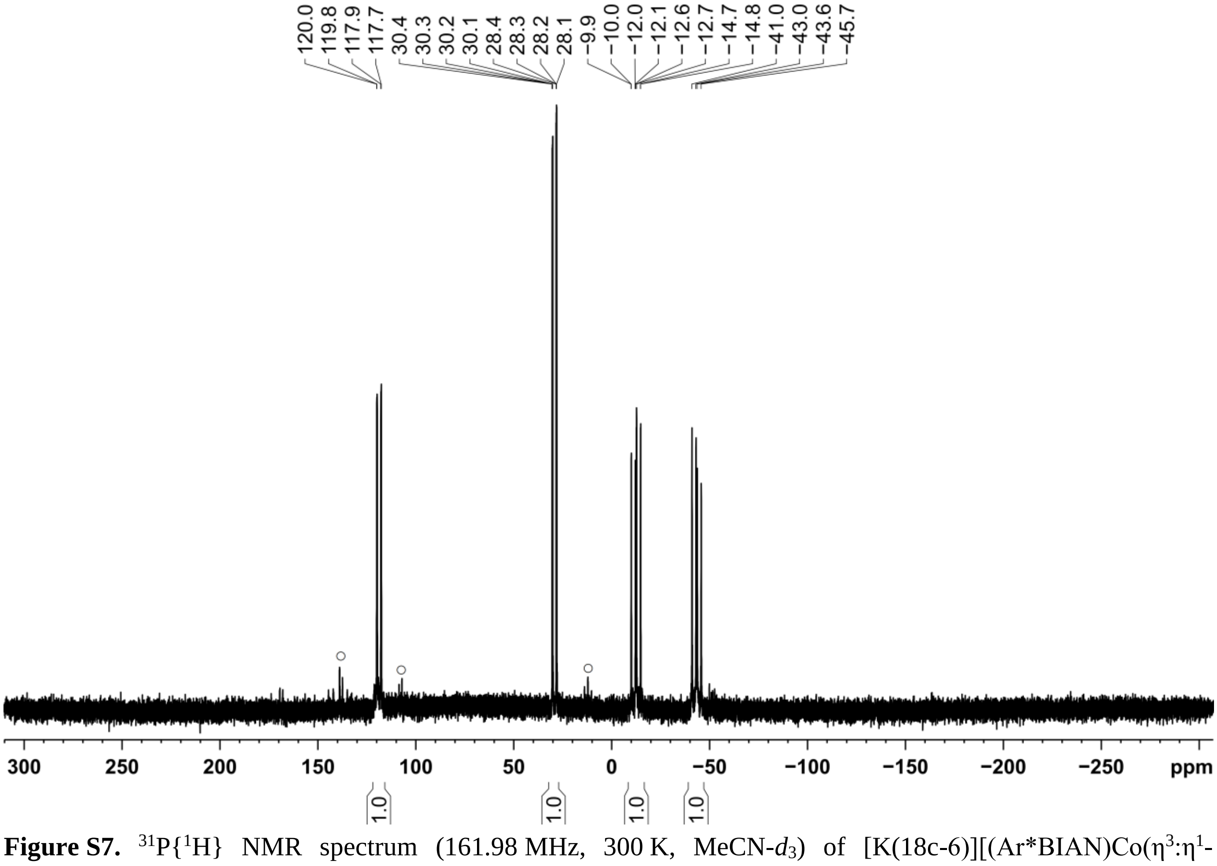

Figure S7. $^{31}P\{^{1}H\}$ NMR spectrum (161.98 MHz, 300 K, MeCN-d_3) of [K(18c-6)][(Ar*BIAN)Co(η^3:η^1-$P_4SN_2(SiMe_3)_2$)] ([K(18c-6)]**4**); ○ unknown impurity.

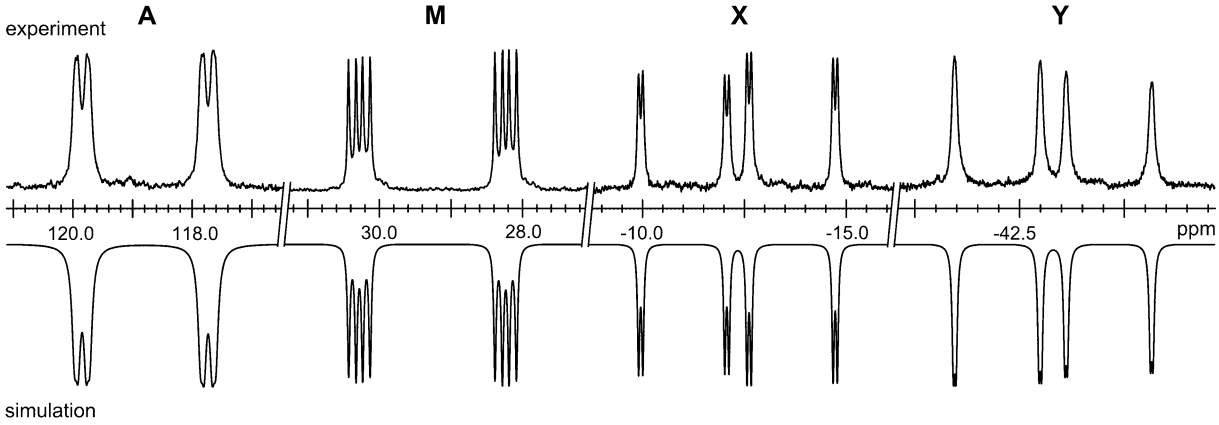

Figure S8. Section of the $^{31}P\{^1H\}$ NMR spectrum (161.98 MHz, 300 K, MeCN-d_3) of [K(18c-6)][(Ar*BIAN)Co(η^3:η^1-$P_4SN_2(SiMe_3)_2$)] ([K(18c-6)]**4**); experimental (upwards) and simulation (downwards).

Table S2. Chemical shifts and coupling constants from the iterative fit of the AMXY spin system and schematic representation of the $CoP_4SN_2(SiMe_3)_2$ core of [K(18c-6)][(Ar*BIAN)Co(η^4-$P_4SN_2(SiMe_3)_2$)] ([K(18c-6)]**4**).

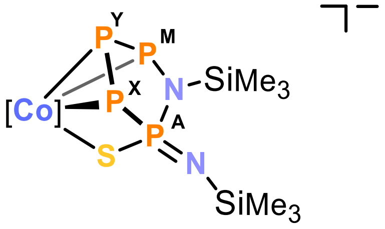	δ(A) = 118.8 ppm	$^1J_{XY}$ = −431.2 Hz
	δ(M) = 29.2 ppm	$^1J_{AX}$ = −342.6 Hz
	δ(X) = −12.4 ppm	$^1J_{MY}$ = −331.1 Hz
	δ(Y) = −43.2 ppm	J_{MX} = 16.8 Hz
		J_{AY} = 10.4 Hz
		J_{AM} = −31.7 Hz

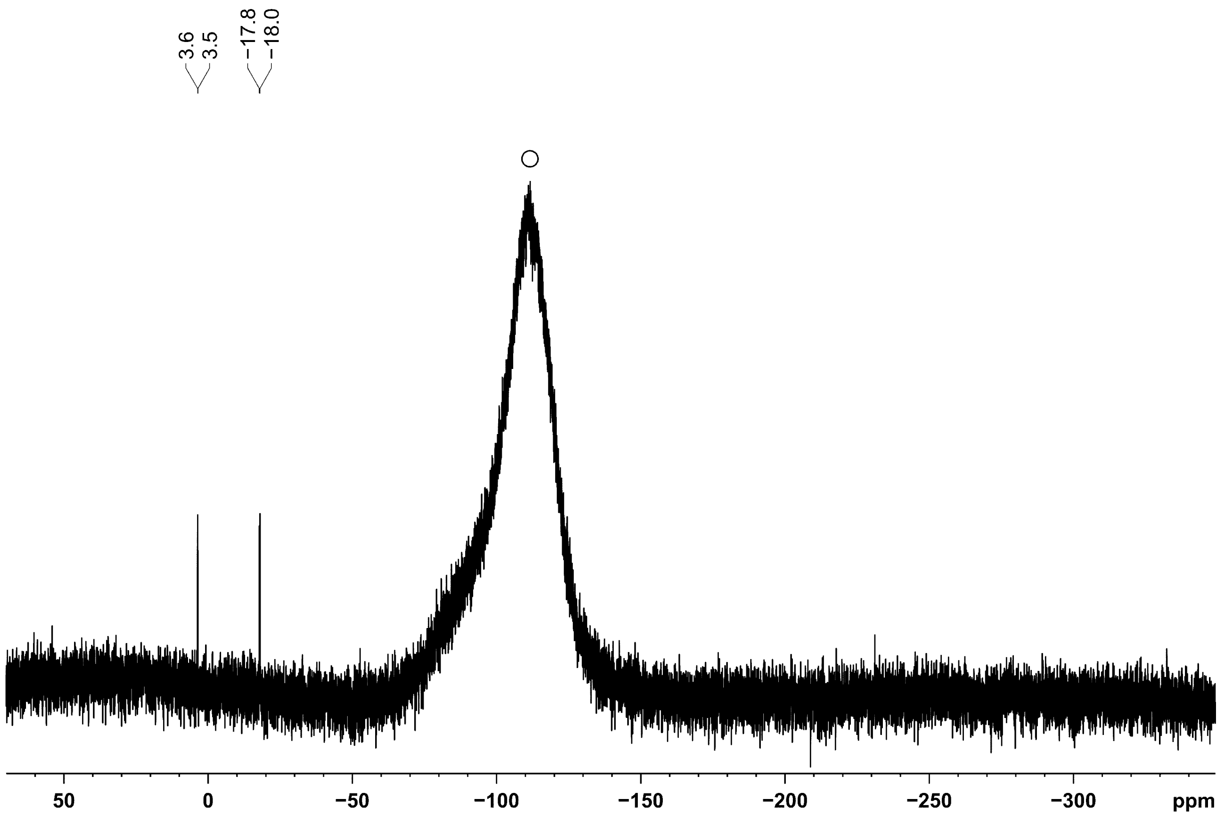

Figure S9. $^{29}Si\{^1H\}$ NMR spectrum (79.49 MHz, 300 K, MeCN-d_3) of [K(18c-6)][(Ar*BIAN)Co(η^3:η^1-$P_4SN_2(SiMe_3)_2$)] ([K(18c-6)]**4**); ○ background signal from glass tube.

[(Ar*BIAN)Co(η^3:η^1-$P_4SN_2(SiMe_3)_3$)] (5)

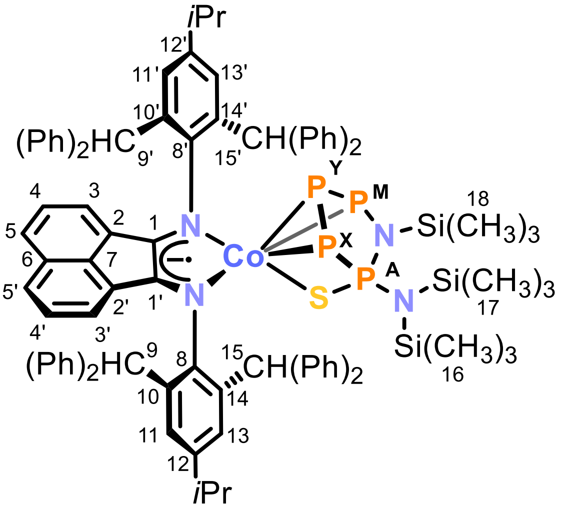

^{1}H NMR (400.13 MHz, 300 K, C_6D_6): δ/ppm = 0.05 (s, 9H, –Si(C^{16}*H*$_3$)$_3$), 0.20 (s, 9H, –Si(C^{18}*H*$_3$)$_3$), 0.38 (s, 9H, –Si(C^{17}*H*$_3$)$_3$), 1.09-1.14 (m, 12H, –CH(C*H*$_3$)$_2$ of *i*Pr), 2.58-2.71 (m, 2H, –C*H*(CH$_3$)$_2$ of *i*Pr), 5.40 (s, 1H, −C$^{9/9'}$*H*(Ph)$_2$), 5.43 (s, 1H, −C$^{9/9'}$*H*(Ph)$_2$), 5.55 (d, $^3J_{HH}$ = 7.1 Hz, 1H, C$^{3/3'}$−*H* of BIAN), 6.02 (d, $^3J_{HH}$ = 7.1 Hz, 1H, C$^{3/3'}$−*H* of BIAN), 6.17-6.21 (m, 1H, C$^{4/4'}$−*H* of BIAN), 6.29-6.33 (m, 1H, C$^{4/4'}$−*H* of BIAN), 6.49-6.56 (m, 4H, C−*H*$_{Ar}$ of Ph), 6.62-6.76 (m, 10H, C−*H*$_{Ar}$ of Ph), 6.92-6.94 (m, 2H, C−*H*$_{Ar}$ of Ph), 7.03-7.21 (m, 10H, d ($^3J_{HH}$ = 8.1 Hz) of C$^{4/4'}$−*H* of BIAN overlapping with C−*H*$_{Ar}$ of Ph overlapping with d ($^3J_{HH}$ = 8.2 Hz) of C$^{4/4'}$−*H* of BIAN overlapping with C_6D_6 solvent signal), 7.27-7.34 (m, 8H, C−*H*$_{Ar}$ of Ph), 7.37-7.37 (m, 1H, C$^{11/11'}$−*H*), 7.41-7.44 (m, 7H, C$^{11/11'}$−*H* overlapping with *C*$^{13/13'}$−H overlapping with C−*H*$_{Ar}$ of Ph), 7.46 (s, 1H, −C$^{15/15'}$*H*(Ph)$_2$), 7.51-7.53 (m, 2H, C−*H*$_{Ar}$ of Ph), 7.72-7.74 (m, 2H, C−*H*$_{Ar}$ of Ph), 7.97 (s, 1H, −C$^{15/15'}$*H*(Ph)$_2$). **^{13}C{^{1}H} NMR** (100.61 MHz, 300 K, C_6D_6): δ/ppm = 2.8 (d, $^3J_{PC}$ = 8.1 Hz, –Si(*C*^{17}H$_3$)$_3$), 5.2 (dd, $^3J_{PC}$ = 5.9 Hz, 3.5 Hz, –Si(*C*^{18}H$_3$)$_3$), 5.7 (d, $^3J_{PC}$ = 1.8 Hz, −Si(*C*^{16}H$_3$)$_3$), 24.0 (s, −CH(*C*H$_3$)$_2$ of *i*Pr), 24.0 (s, −CH(*C*H$_3$)$_2$ of *i*Pr), 24.1 (s, −CH(*C*H$_3$)$_2$ of *i*Pr), 24.1 (s, −CH(*C*H$_3$)$_2$ of *i*Pr), 33.8 (s, –*C*H(CH$_3$)$_2$ of *i*Pr), 33.9 (s, –*C*H(CH$_3$)$_2$ of *i*Pr), 51.1 (s, −*C*$^{9/9'}$H(Ph)$_2$), 51.7 (s, −*C*$^{9/9'}$H(Ph)$_2$), 52.0 (s, −*C*$^{15/15'}$H(Ph)$_2$), 52.4 (s, −*C*^{15}H(Ph)$_2$), 121.8 (s, *C*$^{3/3'}$–H of BIAN), 122.0 (s, *C*$^{3/3'}$–H of BIAN), 124.4 (s, *C*$^{5/5'}$–H of BIAN), 124.4 (s, *C*$^{5/5'}$–H of BIAN), 125.7 (s, *C*$_{Ar}$–H of Ph), 125.7 (s, *C*$_{Ar}$–H of Ph), 125.9 (s, *C*$_{Ar}$−H of Ph), 126.0 (s, *C*$_{Ar}$−H of Ph), 126.1 (s, *C*$_{Ar}$−H of Ph), 126.2 (s, *C*$_{Ar}$−H of Ph), 127.0 (s, *C*$_{Ar}$−H of Ph), 127.7 (s, *C*$^{4/4'}$−H of BIAN overlapping with C_6D_6 solvent signal), 127.7 (s, *C*$_{Ar}$−H of Ph overlapping with C_6D_6 solvent signal), 127.8 (s, *C*$_{Ar}$−H of Ph overlapping with C_6D_6 solvent signal), 127.9 (s, *C*$_{Ar}$−H of Ph overlapping with C_6D_6 solvent signal), 128.0 (s, *C*$^{4/4'}$−H of BIAN overlapping with C_6D_6 solvent signal), 128.0 (s, *C*$_{Ar}$−H of Ph overlapping with C_6D_6 solvent signal), 128.1 (s, *C*$_{Ar}$−H of Ph overlapping with C_6D_6 solvent signal), 128.1 (s, *C*$_{Ar}$−H of Ph overlapping with C_6D_6 solvent signal), 128.2 (s, *C*$_{Ar}$−H of Ph overlapping with C_6D_6 solvent signal), 128.3 (s, *C*$_{Ar}$−H of Ph overlapping with C_6D_6 solvent signal), 128.3 (s, *C*$^{13/13'}$−H overlapping with C_6D_6 solvent signal), 128.4 (s, *C*$^{13/13'}$−H overlapping with C_6D_6 solvent signal), 128.5 (s, *C*$^{11/11'}$−H), 128.6 (s, *C*$^{11/11'}$−H), 130.2 (s, *C*$_{Ar}$−H of Ph), 130.5 (s, *C*^{6} of BIAN), 130.7 (s, *C*$_{Ar}$−H of Ph), 130.8 (s, *C*$_{Ar}$−H of Ph), 130.8 (s, *C*$_{Ar}$−H of Ph), 131.0 (s, *C*$_{Ar}$−H of Ph), 131.0 (s, *C*$_{Ar}$−H of Ph), 131.0 (s, *C*$_{Ar}$−H of Ph), 131.3 (s, *C*$_{Ar}$−H of Ph), 131.4 (s, *C*$_{Ar}$−H of Ph), 132.3 (s, *C*$^{2/2'}$ of BIAN), 132.4 (s, *C*$^{2/2'}$ of BIAN), 132.9 (s, *C*$^{10/10'}$), 135.2 (s, *C*$^{10/10'}$), 136.5 (s, *C*^{7} of BIAN), 137.9 (s, *C*$^{14/14'}$), 138.8 (s, *C*$^{14/14'}$), 142.8 (s, *C*$_{Ar}$ of Ph), 143.1 (s, *C*$_{Ar}$ of Ph), 143.4 (s, *C*$_{Ar}$ of Ph), 143.8 (s, *C*$_{Ar}$ of Ph), 145.3 (s, *C*$^{12/12'}$), 145.5 (s, *C*$^{12/12'}$), 146.1 (s, *C*$_{Ar}$ of Ph), 146.5 (s, *C*$_{Ar}$ of Ph), 147.8 (s, *C*$_{Ar}$ of Ph), 148.1 (s, *C*$_{Ar}$ of Ph), 150.9 (s, *C*$^{8/8'}$−N), 152.2 (s, *C*$^{8/8'}$−N), 163.1 (s, *C*$^{1/1'}$=N of BIAN), 164.2 (s, *C*$^{1/1'}$=N of BIAN). **^{31}P{^{1}H} NMR** (162.04 MHz, 300 K, C_6D_6): (AMXY) spin system δ/ppm = −60.1 (dd, 1P, P_Y), −54.2 - −49.0 (m, 1P, P_X), 47.9-50.8 (m, 1P, P_M), 133.4-135.7 (m, 1P, P_A), for parameters obtained by simulation, see Figure S14 and Table S3. **^{29}Si{^{1}H} NMR** (79.49 MHz, 300 K, C_6D_6): δ/ppm = 7.4 (d, $^2J_{SiP}$ = 10.9 Hz, −*Si*(C^{17}H$_3$)$_3$), 9.3 (s, −*Si*(C^{18}H$_3$)$_3$), 11.6 (d, $^2J_{SiP}$ = 6.1 Hz, −*Si*(C^{16}H$_3$)$_3$).

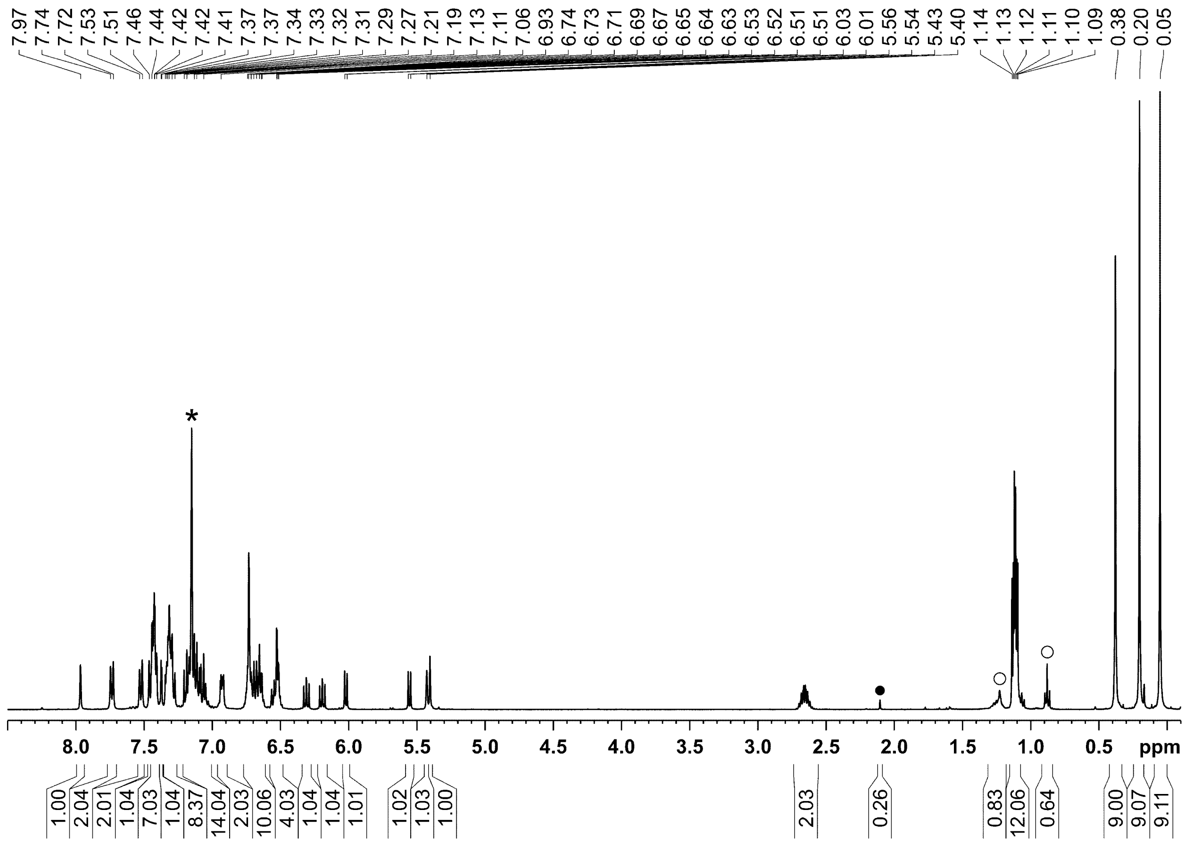

Figure S10. ^{1}H NMR spectrum (400.30 MHz, 300 K, C_6D_6) of [(Ar*BIAN)Co(η^3:η^1-$P_4SN_2(SiMe_3)_3$)] (**5**); ○ *n*-hexane, • toluene, * C_6D_6.

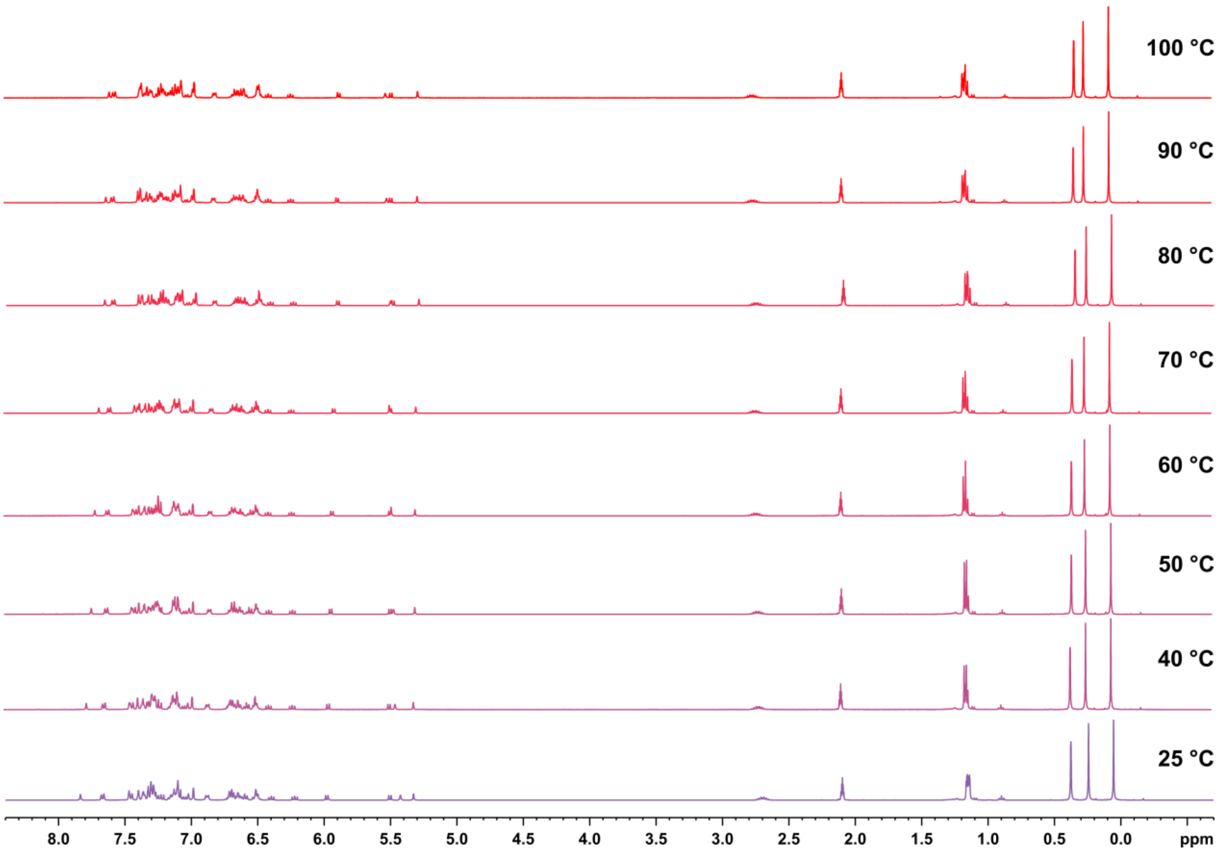

Figure S11. ^{1}H NMR monitoring (400.13 MHz, toluene-d_8) of [(Ar*BIAN)Co(η^3:η^1-$P_4SN_2(SiMe_3)_3$)] (**5**).

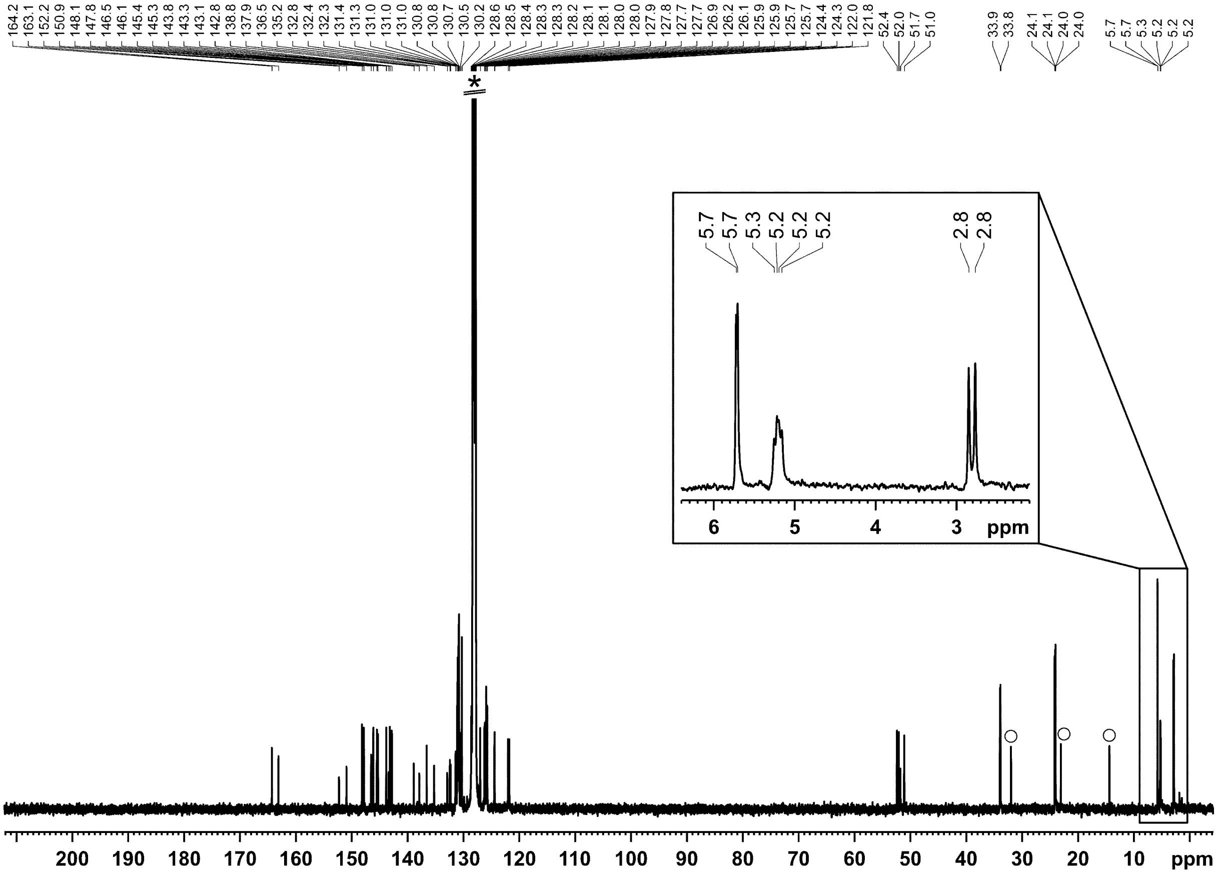

Figure S12. $^{13}C\{^{1}H\}$ NMR spectrum (100.60 MHz, 300 K, C_6D_6) of [(Ar*BIAN)Co(η^3:η^1-$P_4SN_2(SiMe_3)_3$)] (**5**); ○ *n*-hexane, * C_6D_6.

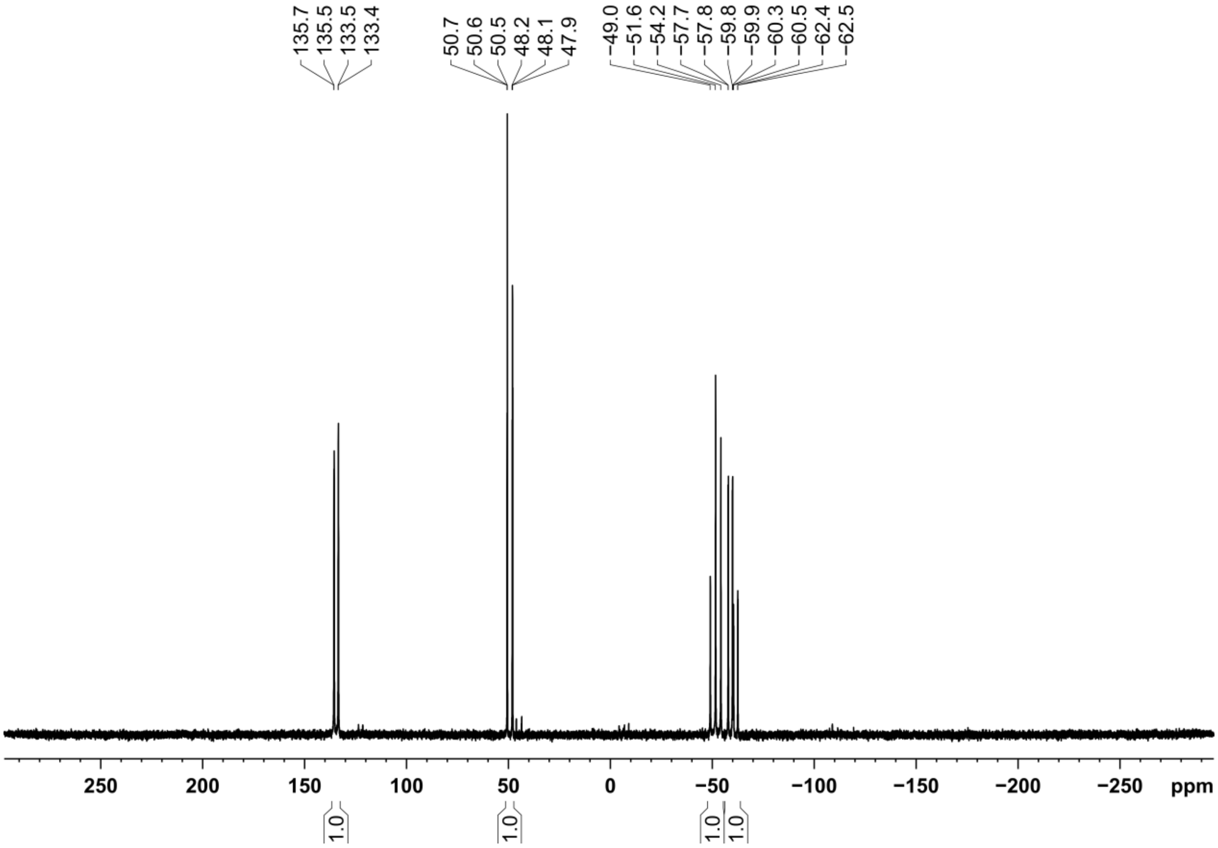

Figure S13. $^{31}P\{^{1}H\}$ NMR spectrum (162.04 MHz, 300 K, C_6D_6) of [(Ar*BIAN)Co(η^3:η^1-$P_4SN_2(SiMe_3)_3$)] (**5**).

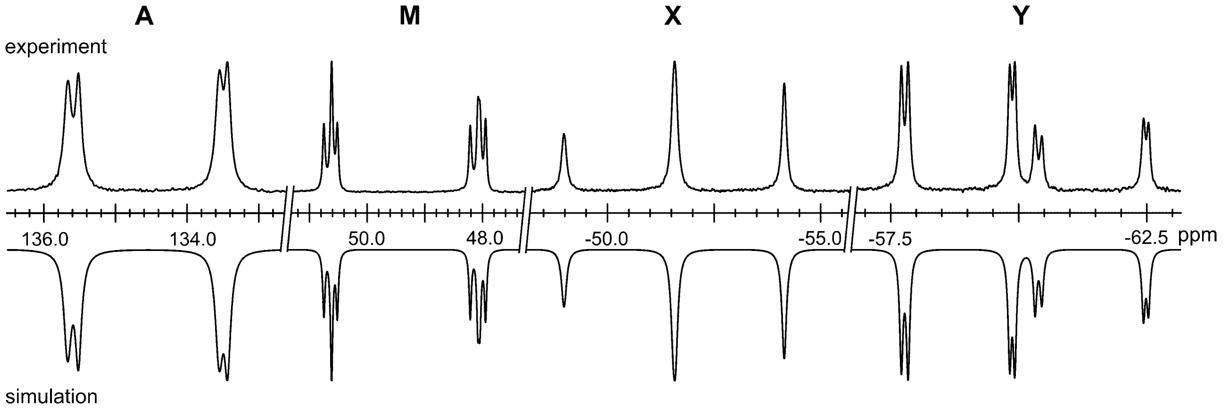

Figure S14. Section of the $^{31}P\{^{1}H\}$ NMR spectrum (162.04 MHz, 300 K, C_6D_6) of [(Ar*BIAN)Co(η^3:η^1-$P_4SN_2(SiMe_3)_3$)] (**5**); experimental (upwards) and simulation (downwards).

Table S3. Chemical shifts and coupling constants from the iterative fit of the AMXY spin system and schematic representation of the $CoP_4SN_2(SiMe_3)_3$ core of [(Ar*BIAN)Co(η^3:η^1-$P_4SN_2(SiMe_3)_3$)] (**5**).

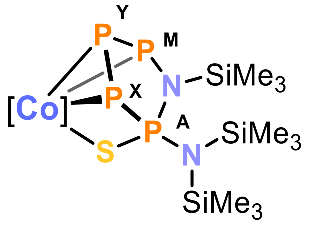	δ(A) = 134.5 ppm	$^{1}J_{XY}$ = −422.6 Hz
	δ(M) = 49.3 ppm	$^{1}J_{MY}$ = −425.4 Hz
	δ(X) = −51.8 ppm	$^{1}J_{AX}$ = −349.5 Hz
	δ(Y) = −59.9 ppm	J_{MX} = 30.6 Hz
		J_{AY} = 10.9 Hz
		J_{AM} = −21.3 Hz

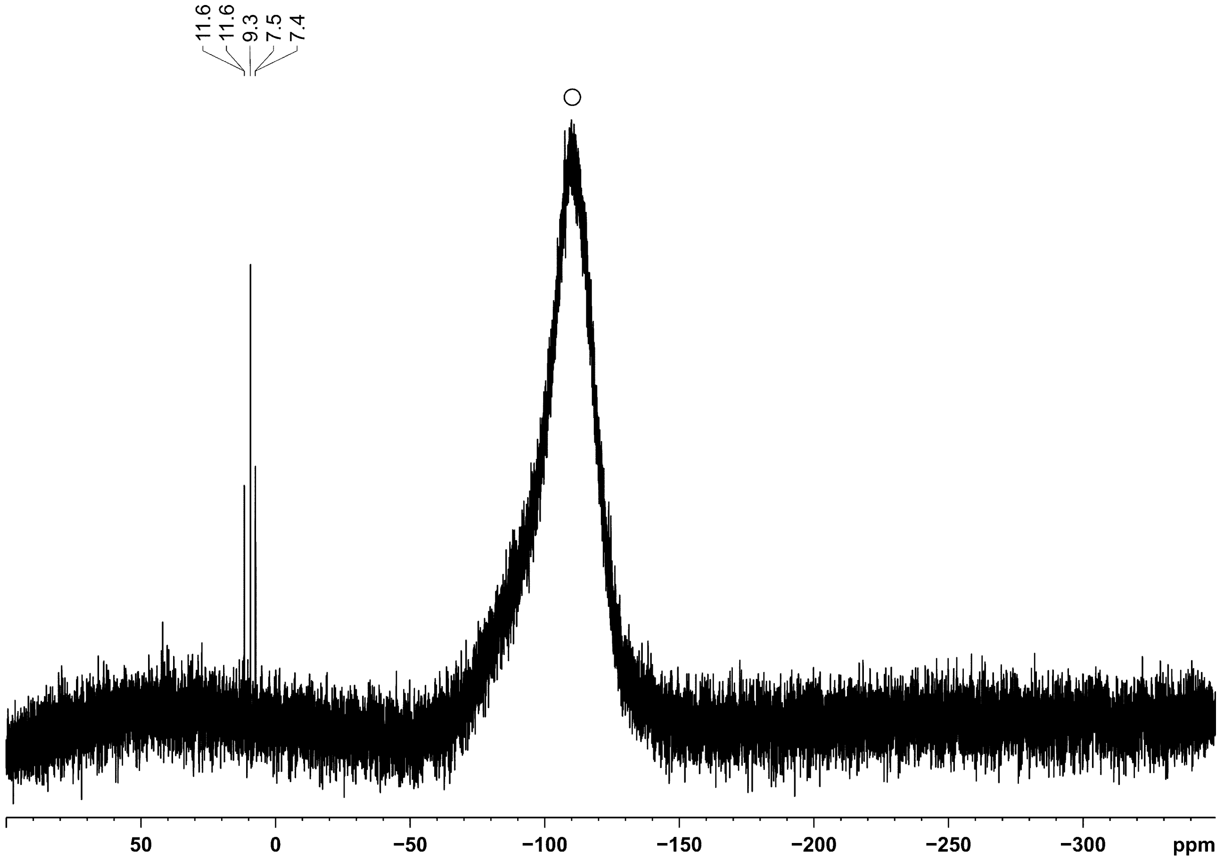

Figure S15. $^{29}Si\{^{1}H\}$ NMR spectrum (79.49 MHz, 300 K, C_6D_6) of [(Ar*BIAN)Co(η^3:η^1-$P_4SN_2(SiMe_3)_3$)] (**5**); ○ background signal from glass tube.

[(Ar*BIAN)Co(η^3:η^1-P_4C(S)N(*Cy*)C(O)*t*Bu)]] (6a)

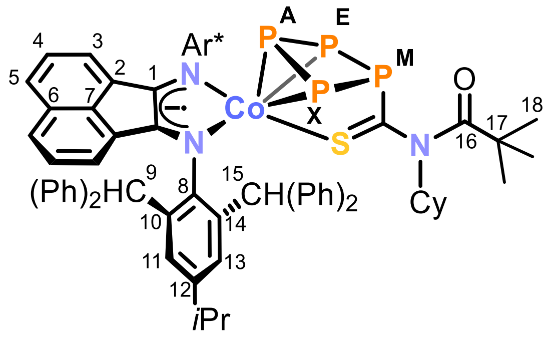

^{1}H NMR (400.13 MHz, 300 K, C_6D_6): δ/ppm = 0.60-0.65 (m, 5H, C*H*$_2$ of Cy), 0.92 (s, 9H,−C(C^{18}*H*$_3$)$_3$ of *t*Bu), 1.02-1.05 (m, 12H, −CH(C*H*$_3$)$_2$ of *i*Pr), 1.16-1.22 (m, 3H, C*H*$_2$ of Cy), 1.46-1.49 (m, 2H, C*H*$_2$ of Cy), 2.58 (sept, $^3J_{HH}$ = 6.9 Hz, 2H, −C*H*(CH$_3$)$_2$ of *i*Pr), 3.43-3.48 (m, 1H, C−*H* of Cy), 5.50 (s, 2H, −C^9*H*(Ph)$_2$), 5.89 (d, $^3J_{HH}$ = 7.1 Hz, 2H, C^3−*H* of BIAN), 6.22-6.26 (m, 2H, C^4−*H* of BIAN), 6.64-6.65 (m, 6H, C−*H*$_{Ar}$ of Ph), 6.71-6.75 (m, 2H, C−*H*$_{Ar}$ of Ph), 6.82-6.98 (m, 10H, C−*H*$_{Ar}$ of Ph), 7.06-7.15 (m, 14H, C−*H*$_{Ar}$ of Ph overlapping with C_6D_6 solvent signal), 7.19 (d, $^3J_{HH}$ = 8.2 Hz, 2H, C^5−*H* of BIAN), 7.29-7.29 (m, 2H, C^{11}−*H*), 7.36-7.37 (m, 2H, C^{13}−*H*), 7.64-7.65 (m, 8H, C−*H*$_{Ar}$ of Ph), 7.92 (s, 2H, −C^{15}*H*(Ph)$_2$). **^{13}C{^{1}H} NMR** (100.66 MHz, 300 K, C_6D_6): δ/ppm = 24.4 (s, −CH(*C*H$_3$)$_2$ of *i*Pr), 24.5 (s, −CH(*C*H$_3$)$_2$ of *i*Pr), 26.1 (s, *C*H$_2$ of Cy), 26.6 (s, *C*H$_2$ of Cy), 29.0 (s, −C(*C*^{18}H$_3$)$_3$ of *t*Bu), 31.2 (s, *C*H$_2$ of Cy), 34.4 (s, −*C*H(CH$_3$)$_2$ of *i*Pr), 44.1 (s, −*C*^{17}(CH$_3$)$_3$ of *t*Bu), 51.7 (s, −*C*^{9}H(Ph)$_2$), 53.3 (s, −*C*^{15}H(Ph)$_2$), 67.1 (s, *C*H of Cy), 122.6 (s, *C*^{3}−H of BIAN), 125.4 (s, *C*^{5}−H of BIAN), 126.5 (s, *C*$_{Ar}$−H of Ph), 126.6 (s, *C*$_{Ar}$−H of Ph), 126.9 (s, *C*$_{Ar}$−H of Ph), 127.3 (s, *C*$_{Ar}$−H of Ph), 128.4 (s, *C*$_{Ar}$−H of Ph overlapping with C_6D_6 solvent signal), 128.5 (s, *C*^{13}−H overlapping with C_6D_6 solvent signal), 128.6 (s, *C*^{4}−H of BIAN overlapping with C_6D_6 solvent signal), 128.6 (s, *C*$_{Ar}$−H of Ph overlapping with C_6D_6 solvent signal), 128.8 (s, *C*^{11}−H overlapping with C_6D_6 solvent signal), 128.9 (s, *C*$_{Ar}$−H of Ph overlapping with C_6D_6 solvent signal), 129.2 (s, *C*$_{Ar}$−H of Ph), 130.6 (s, *C*$_{Ar}$−H of Ph), 130.9 (s, *C*$_{Ar}$−H of Ph), 131.1 (s, *C*^{6} of BIAN overlapping with *C*$_{Ar}$−H of Ph), 131.1 (s, *C*^{6} of BIAN overlapping with *C*$_{Ar}$−H of Ph), 131.4 (s, *C*$_{Ar}$−H of Ph), 132.6 (s, *C*^{2} of BIAN), 135.2 (s, *C*^{10}), 136.8 (s, *C*^{7} of BIAN), 139.0 (s, *C*^{14}), 143.5 (s, *C*$_{Ar}$ of Ph), 145.3 (s, *C*$_{Ar}$ of Ph), 146.0 (s, *C*$_{Ar}$ of Ph), 146.3 (s, *C*^{12}), 148.5 (s, *C*$_{Ar}$ of Ph), 149.7 (s, *C*^{8}−N), 164.3 (s, *C*^{1}=N of BIAN), 183.1 (s, *C*^{16}(O)*t*Bu); *C*=S: not detected. **^{31}P{^{1}H} NMR** (162.04 MHz, 300 K, C_6D_6): δ/ppm = 86.3 (t, 1P), 93.0 (br s, $\Delta\nu_{½}$ = 2500 Hz, 2P), 117.3 (t, 1P); (161.98 MHz, toluene-*d*$_8$, 213 K): (AEMX) spin system δ/ppm = 77.6 (dd, 1P, $P_{E/X}$), 85.5 (t, 1P, P_M), 105.3 (dd, 1P, $P_{E/X}$), 118.0 (t, 1P, P_A), for parameters obtained by simulation, see Figure S20 and Table S4. Spin system was assigned based on DFT calculated values (vide infra).

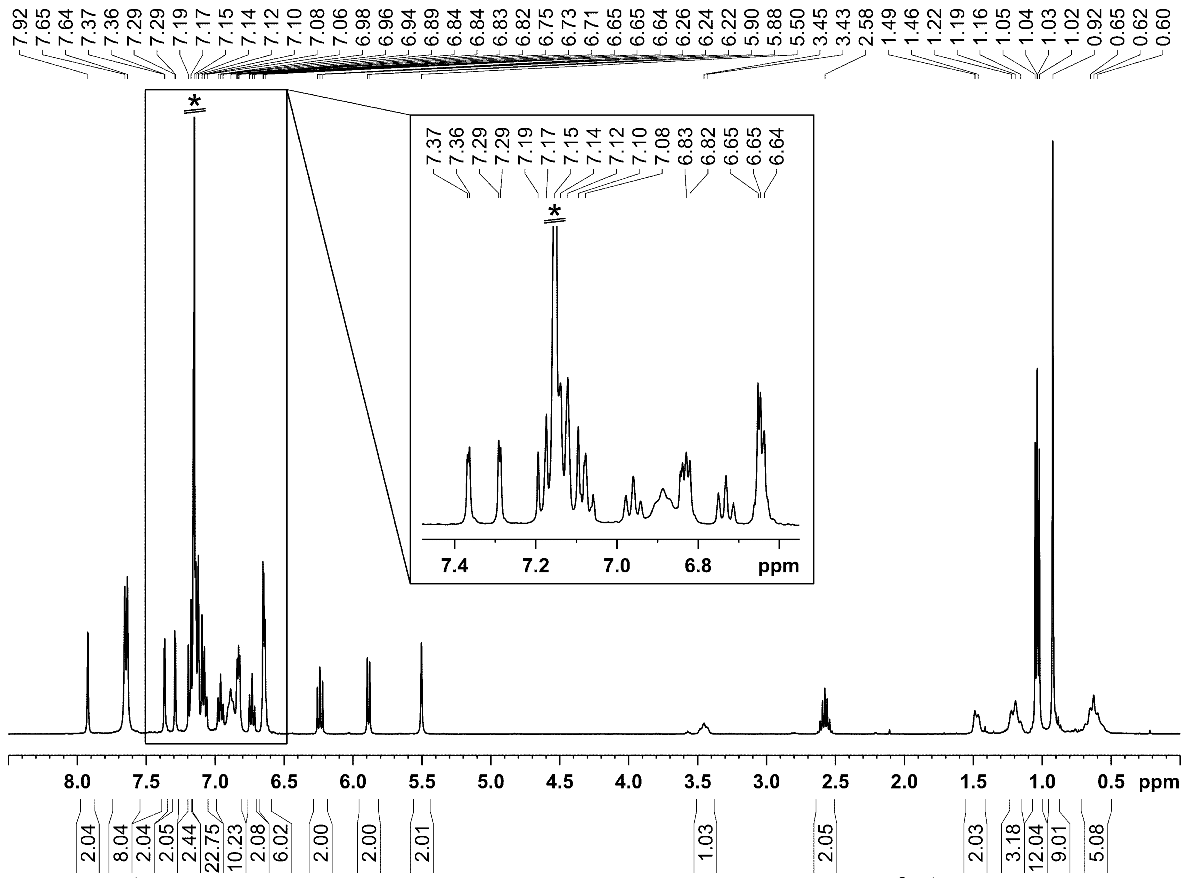

Figure S16. ^{1}H NMR spectrum (400.13 MHz, 300 K, C_6D_6,) of [(Ar*BIAN)Co(η^3:η^1-P_4C(S)N(Cy)C(O)*t*Bu)] (**6a**); * C_6D_6.

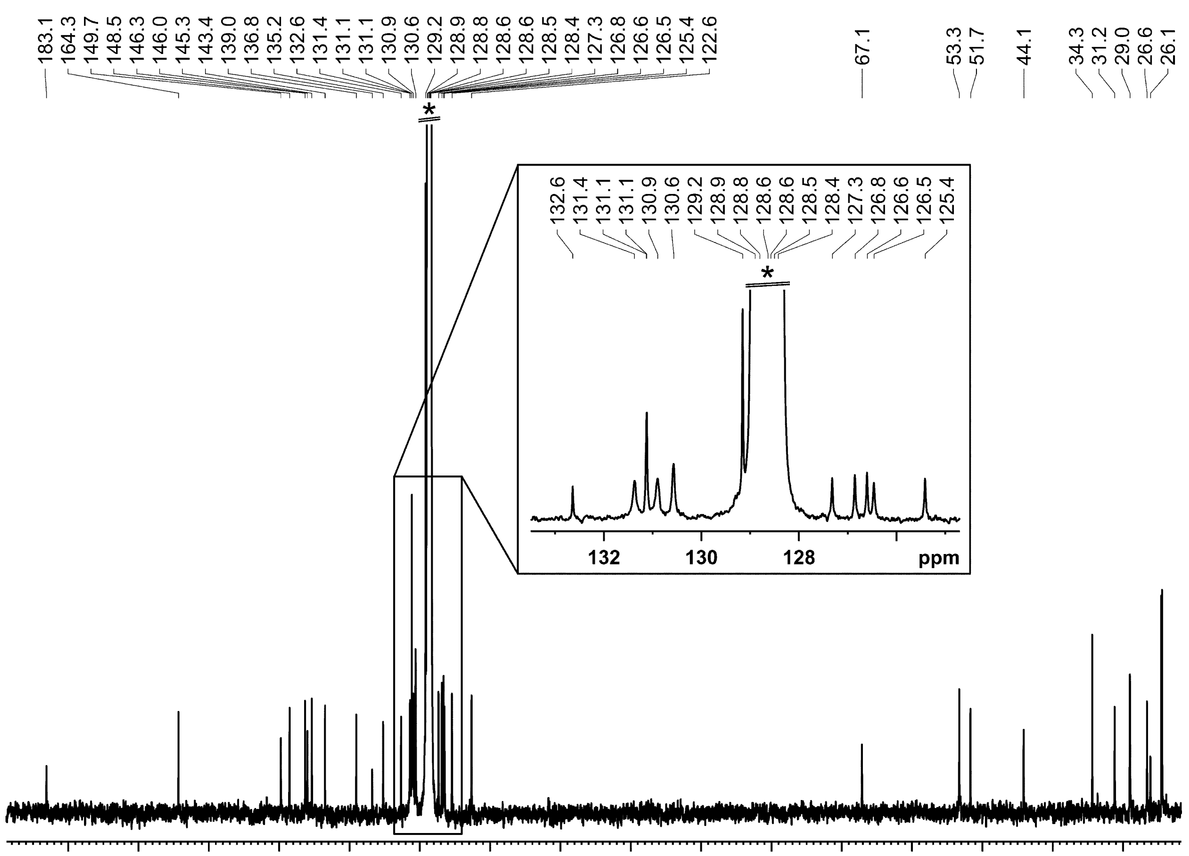

Figure S17. ^{13}C{^{1}H} NMR spectrum (100.66 MHz, 300 K, C_6D_6) of [(Ar*BIAN)Co(η^3:η^1-P_4C(S)N(Cy)C(O)*t*Bu)] (**6a**); * C_6D_6.

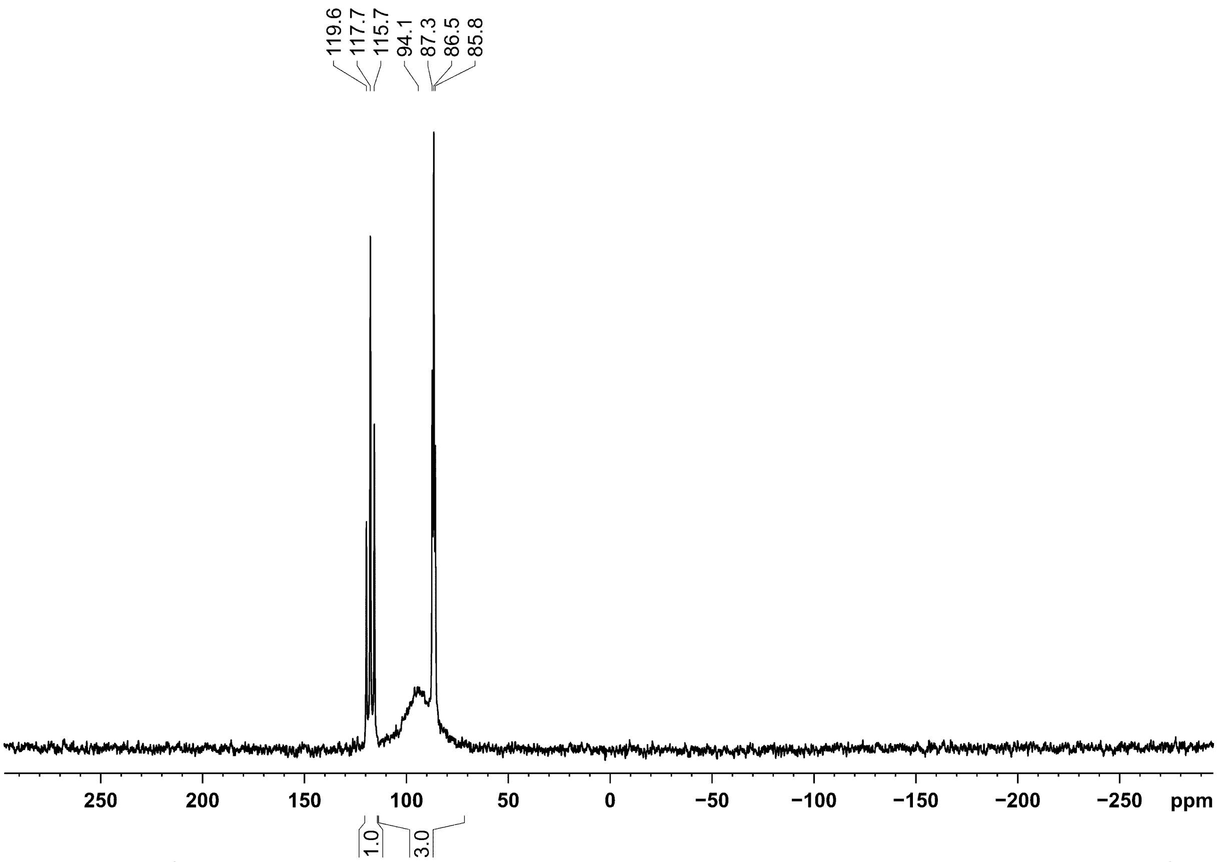

Figure S18. $^{31}P\{^{1}H\}$ NMR spectrum (162.04 MHz, 300 K, C_6D_6) of [(Ar*BIAN)Co(η^3:η^1-P_4C(S)N(Cy)C(O)*t*Bu)] (**6a**).

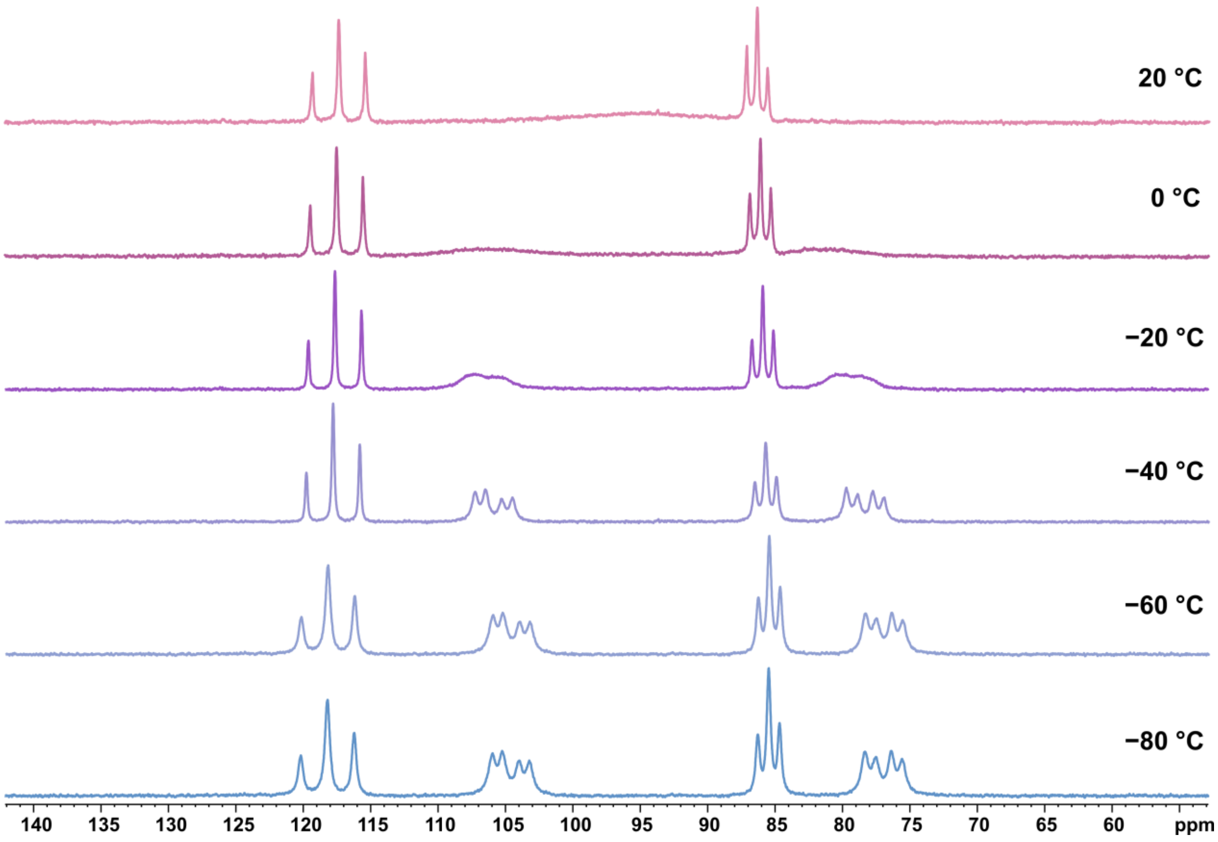

Figure S19. $^{31}P\{^{1}H\}$ NMR monitoring (161.98 MHz, toluene-d_8) of [(Ar*BIAN)Co(η^3:η^1-P_4C(S)N(Cy)C(O)*t*Bu)] (**6a**).

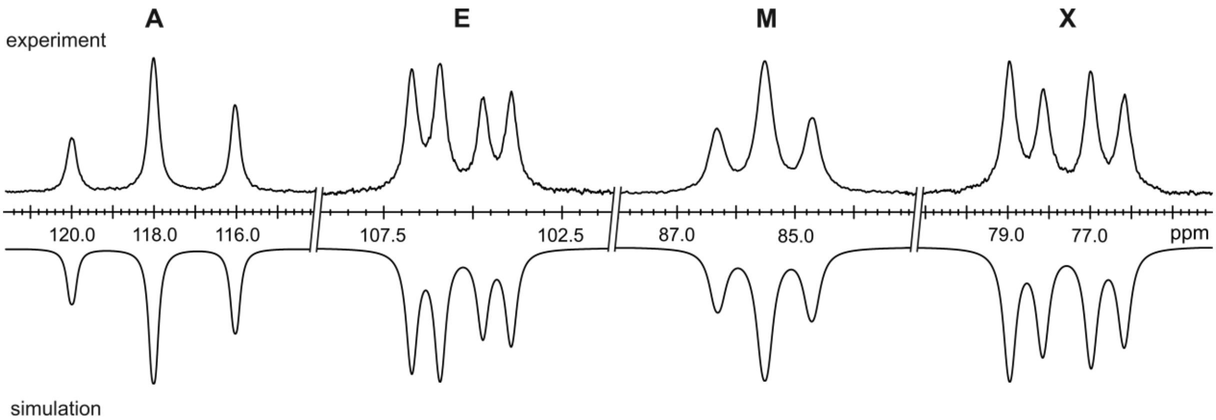

Figure S20. Section of the $^{31}P\{^{1}H\}$ NMR spectrum (161.98 MHz, 213 K, toluene-d_8) of [(Ar*BIAN)Co(η^3:η^1-P_4C(S)N(Cy)C(O)*t*Bu)] (**6a**); experimental (upwards) and simulation (downwards).

Table S4. Chemical shifts and coupling constants from the iterative fit of the AEMX spin system and schematic representation of the CoP_4C(S)N(Cy)C(O)*t*Bu core of [(Ar*BIAN)Co(η^3:η^1-P_4C(S)N(Cy)C(O)*t*Bu)] (**6a**).

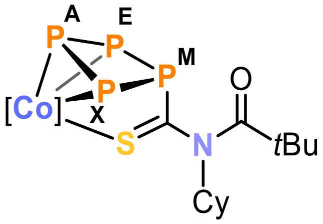	δ(A) = 117.9 ppm	$^1J_{AE}$ = −324.3 Hz
	δ(E) = 105.4 ppm	$^1J_{AX}$ = −321.3 Hz
	δ(M) = 85.5 ppm	$^1J_{EM}$ = −129.0 Hz
	δ(X) = 77.6 ppm	$^1J_{MX}$ = −132.9 Hz
		$^2J_{AM}$ = 18.0 Hz
		$^2J_{EX}$ = 19.7 Hz

[(Ar*BIAN)Co(η^3:η^1-P_4C(S)N(Ph)C(O)*t*Bu)] (6b)

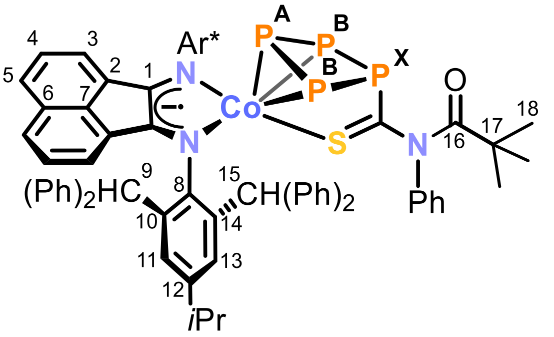

^{1}H NMR (400.13 MHz, 300 K, C_6D_6,): δ/ppm = 0.76 (s, 9H,−C($C^{18}H_3$)$_3$ of *t*Bu), 1.00-1.03 (m, 12H, −CH(CH_3)$_2$ of *i*Pr), 2.55 (sept, $^3J_{HH}$ = 6.9 Hz, 2H, −C*H*(CH_3)$_2$ of *i*Pr), 5.45 (s, 2H, −C^9H(Ph)$_2$), 5.80-5.82 (m, 2H, C−H_{ortho} of PhNCS), 5.85 (d, $^3J_{HH}$ = 7.1 Hz, 2H, C^3−*H* of BIAN), 6.21-6.25 (m, 2H, C^4−*H* of BIAN), 6.57-6.82 (m, 17H, C−$H_{meta/para}$ of PhNCS overlapping with C−H_{Ar} of Ph), 7.01-7.16 (m, 16H, C^5−*H* of BIAN overlapping with C−H_{Ar} of Ph overlapping with C_6D_6 solvent signal), 7.25-7.29 (m, 6H, C^{11}−*H* overlapping with C−H_{Ar} of Ph), 7.31-7.34 (m, 6H, C^{13}−*H* overlapping with C−H_{Ar} of Ph), 7.57 (s, 2H, −$C^{15}H$(Ph)$_2$), 7.74-7.76 (br m, 4H, C−H_{Ar} of Ph). **$^{13}C\{^1H\}$ NMR** (100.61 MHz, 273 K, toluene-d_8): δ/ppm = 24.3 (s, −CH(CH_3)$_2$ of *i*Pr), 24.5 (s, −CH(CH_3)$_2$ of *i*Pr), 28.5 (s, −C($C^{18}H_3$)$_3$ of *t*Bu), 34.3 (s, −CH(CH_3)$_2$ of *i*Pr), 43.3 (s, −C^{17}(CH_3)$_3$ of *t*Bu), 51.4 (s, −C^9H(Ph)$_2$), 52.9 (s, −C^{15}H(Ph)$_2$), 122.5 (s, C^3−H of BIAN), 125.5 (s, C^5−H of BIAN overlapping with toluene-d_8 solvent signal), 126.5 (s, C_{Ar}−H of Ph), 126.5 (s, C_{Ar}−H of PhNCS), 126.6 (s, C_{Ar}−H of Ph), 127.2 (s, C_{Ar}−H of Ph), 127.9 (s, C_{Ar}−H of Ph), 128.3 (s, C^4−H of BIAN overlapping with toluene-d_8 solvent signal), 128.3 (s, C_{ortho}−H of PhNCS overlapping with toluene-d_8 solvent signal), 128.4 (s, C_{Ar}−H of Ph overlapping with toluene-d_8 solvent signal), 128.5 (s, C^{11}−H overlapping with toluene-d_8 solvent signal), 128.6 (s, C_{Ar}−H of Ph overlapping with toluene-d_8 solvent signal), 128.8 (s, C^{13}−H overlapping with toluene-d_8 solvent signal), 128.7 (s, C_{Ar}−H of Ph overlapping with toluene-d_8 solvent signal), 128.9 (s, C_{Ar}−H of Ph overlapping with toluene-d_8 solvent signal), 130.1 (s, C_{Ar}−H of PhNCS), 130.2 (s, C_{Ar}−H of Ph), 130.7 (s, C^6 of BIAN), 130.9 (s, C_{Ar}−H of Ph), 131.1 (s, C_{Ar}−H of Ph), 132.0 (s, C^2 of BIAN), 135.2 (s, C^{10}), 136.6 (s, C^7 of BIAN), 138.7 (s, C^{14}), 141.6 (s, C_{ipso} of PhNCS), 142.7 (s, C_{Ar} of Ph), 145.0 (s, C_{Ar} of Ph), 145.8 (s, C_{Ar} of Ph), 146.2 (s, C^{12}), 148.5 (s, C_{Ar} of Ph), 149.0 (s, C^8−N), 164.4 (s, C^1=N of BIAN),

182.2 (s, C^{16}=O of −C(O)tBu); C=S: not detected. **^{31}P{^{1}H} NMR** (162.04 MHz, 300 K, C_6D_6): (AB_2X) spin system δ/ppm = 95.5-98.8 (m, 1P, P_X), 103.8-109.5 (m, 3P, P_A/P_B), for parameters obtained by simulation, see Figure S24 and Table S5.

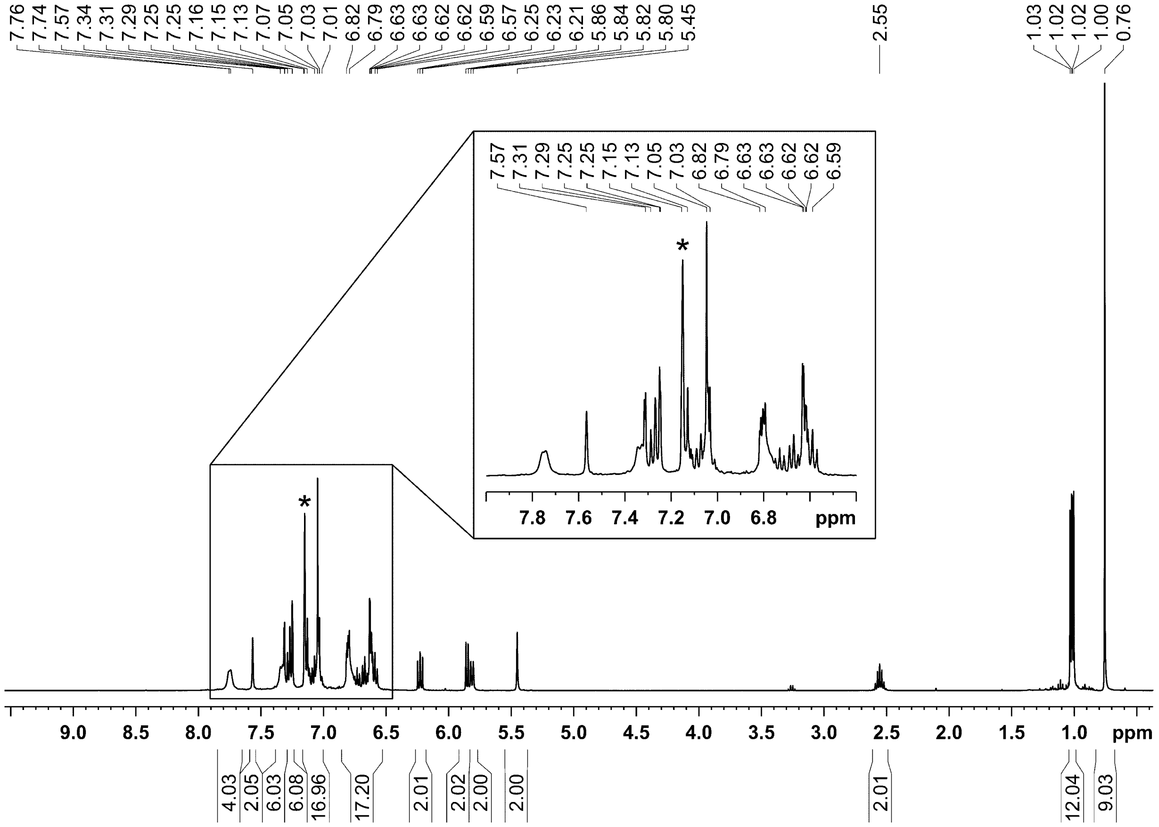

Figure S21. ^{1}H NMR spectrum (400.13 MHz, 300 K, C_6D_6) of [(Ar*BIAN)Co(η^3:η^1-P_4C(S)N(Ph)C(O)tBu)] (**6b**); * C_6D_6.

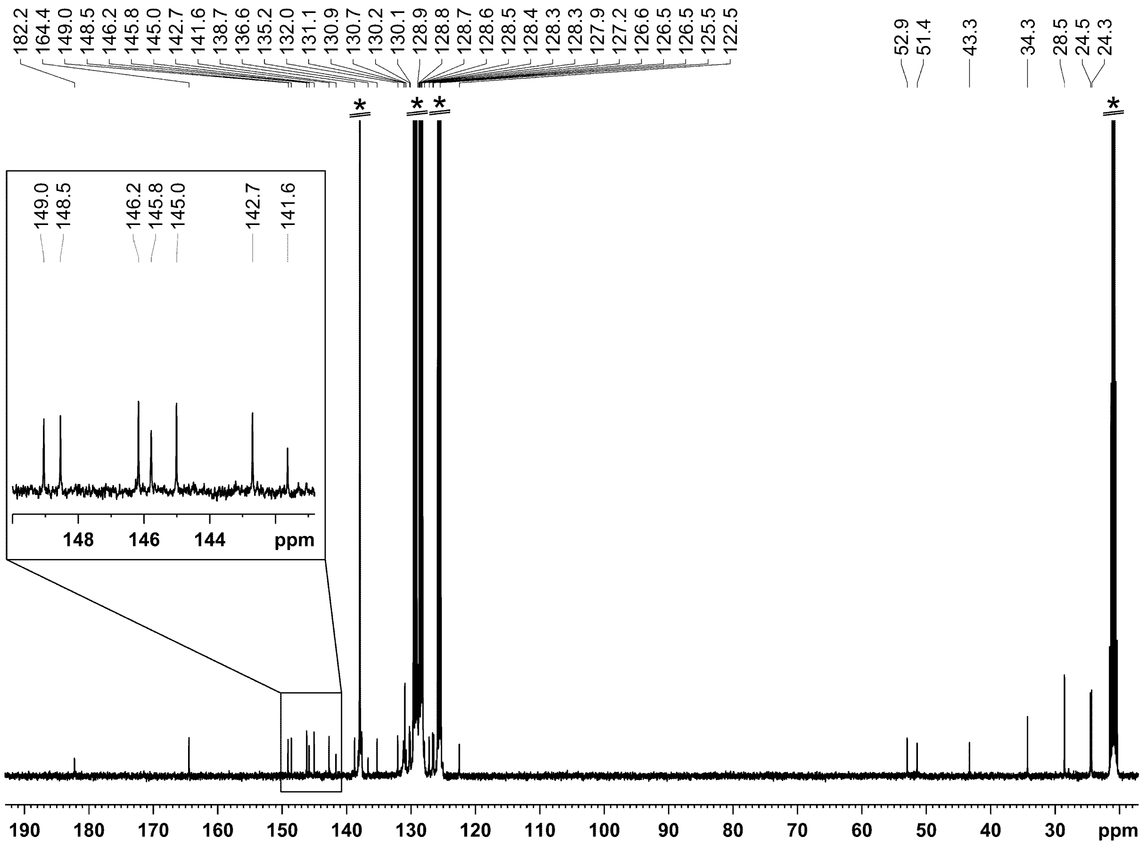

Figure S22. $^{13}C\{^{1}H\}$ NMR spectrum (100.61 MHz, 273 K, toluene-d_8) of [(Ar*BIAN)Co(η^3:η^1-P_4C(S)N(Ph)C(O)*t*Bu)] (**6b**); * toluene-d_8.

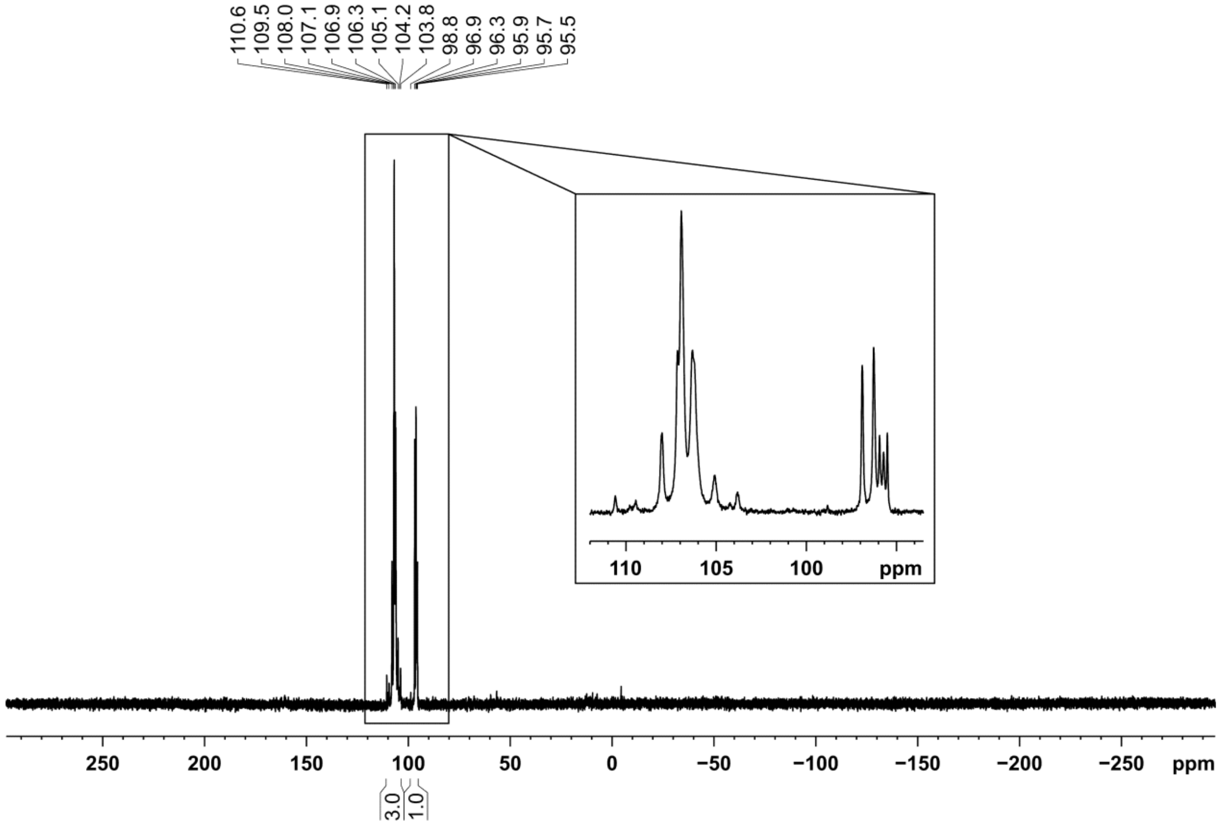

Figure S23. $^{31}P\{^{1}H\}$ NMR spectrum (162.04 MHz, 300 K, C_6D_6) of [(Ar*BIAN)Co(η^3:η^1-P_4C(S)N(Ph)C(O)*t*Bu)] (**6b**).

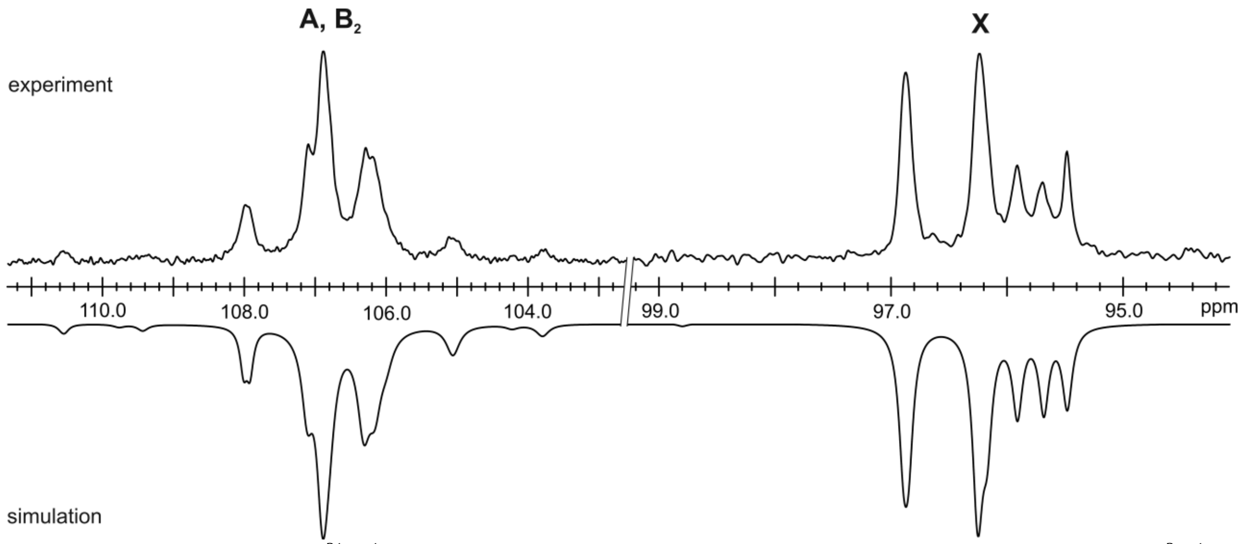

Figure S24. Section of the $^{31}P\{^{1}H\}$ NMR spectrum (162.04 MHz, 300 K, C_6D_6) of [(Ar*BIAN)Co(η^3:η^1-P_4C(S)N(Ph)C(O)*t*Bu)] (**6b**); experimental (upwards) and simulation (downwards).

Table S5. Chemical shifts and coupling constants from the iterative fit of the AB_2X spin system and schematic representation of the $CoP_4C(S)N(Ph)C(O)$*t*Bu core of [(Ar*BIAN)Co(η^3:η^1-P_4C(S)N(Ph)C(O)*t*Bu)] (**6b**).

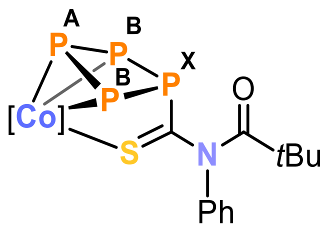	δ(A) = 108.0 ppm	$^1J_{AB}$ = −325.4 Hz
	δ(B) = 106.0 ppm	$^1J_{BX}$ = −121.2 Hz
	δ(X) = 96.2 ppm	$^2J_{AX}$ = 14.5 Hz

Additional experiments

Reaction of [(Ar*BIAN)Co(η^3:η^1-$P_4SN_2(SiMe_3)_3$)] (5) with [*n*Bu_4N]CN

To a purple solution of [(Ar*BIAN)Co(η^3:η^1-$P_4SN_2(SiMe_3)_3$)] (**5**) (13 mg, 0.008 mmol, 1.0 equiv.) in C_6D_6 (0.7 mL), [*n*Bu_4N]CN (2.3 mg, 0.008 mmol, 1.0 equiv.) was added. The color changed immediately to blue and after 3 h the reaction mixture was transferred to a J. valve NMR tube and analyzed by $^{31}P\{^1H\}$ NMR spectroscopy (see Figure S25).

Reaction of [(Ar*BIAN)Co(η^3:η^1-$P_4SN_2(SiMe_3)_3$)] (5) with KOPh/18c-6

To a purple solution of [(Ar*BIAN)Co(η^3:η^1-$P_4SN_2(SiMe_3)_3$)] (**5**) (20 mg, 0.013 mmol, 1.0 equiv.) in C_6D_6 (0.7 mL) KOPh (1.7 mg, 0.013 mmol, 1.0 equiv.) and [18]crown-6 (18c-6, 3.3 mg, 0.013 mmol, 1.0 equiv.) was added. The color changed to blue and after 3 h the reaction mixture was transferred to a J. valve NMR tube and analyzed by $^{31}P\{^1H\}$ NMR spectroscopy (see Figure S25).

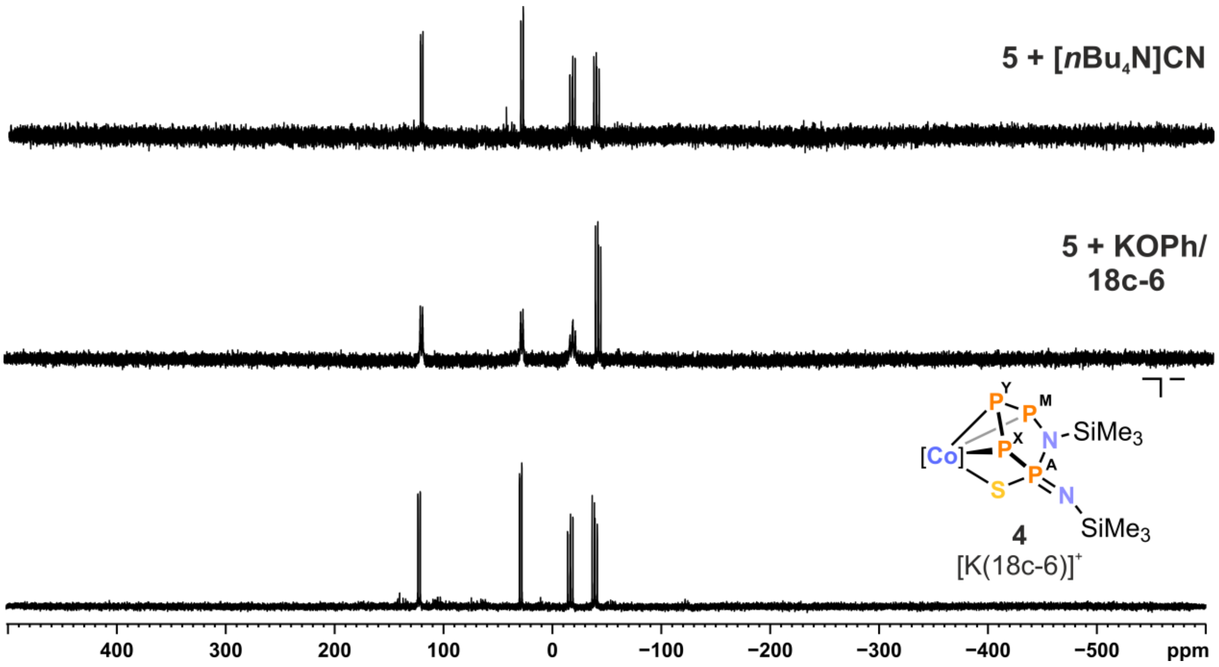

Figure S25. $^{31}P\{^1H\}$ NMR spectra (162.04 MHz, 300 K, C_6D_6) of the reactions between [(Ar*BIAN)Co(η^3:η^1-$P_4SN_2(SiMe_3)_3$)] (**5**) with [*n*Bu_4N]CN (*top*) and KOPh/18c-6 (*middle*); as well as [K(18c-6)][(Ar*BIAN)Co(η^3:η^1-$P_4SN_2(SiMe_3)_2$)] ([K(18c-6)]**4**) (*bottom*) for comparison.

UV-Vis spectra

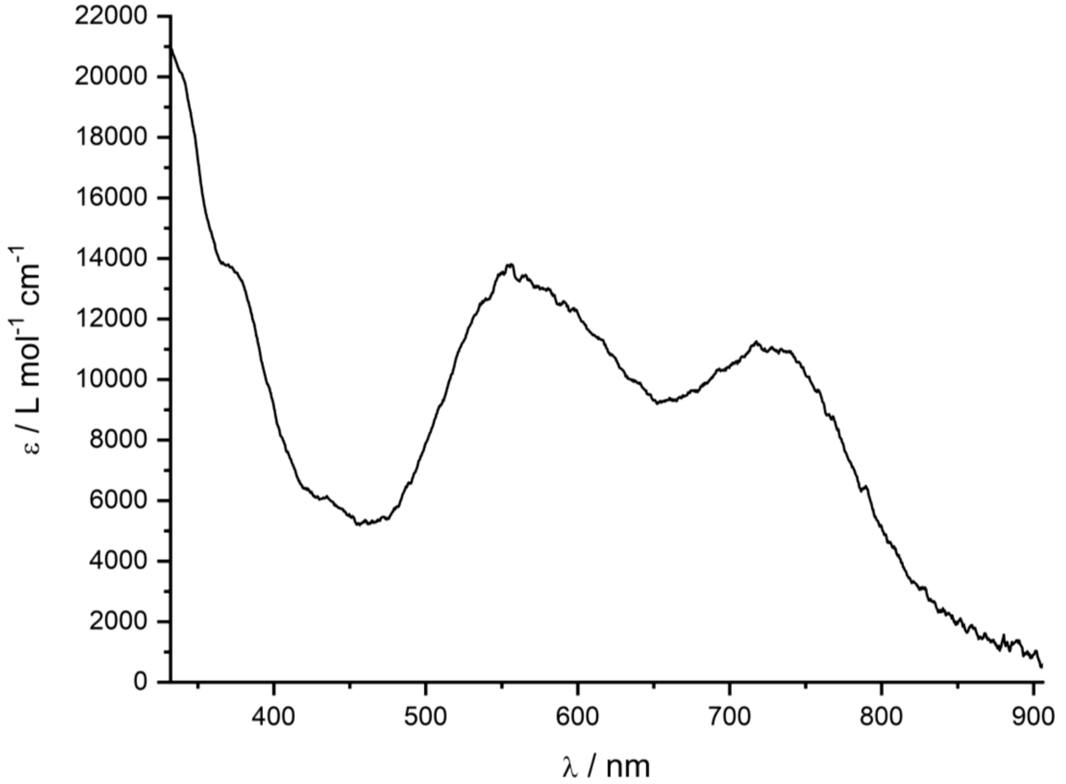

Figure S26. UV/Vis spectrum of [K(18c-6)][(Ar*BIAN)Co(η^3:η^1-P_4CS_2)] ([K(18c-6)]**3**) recorded in THF.

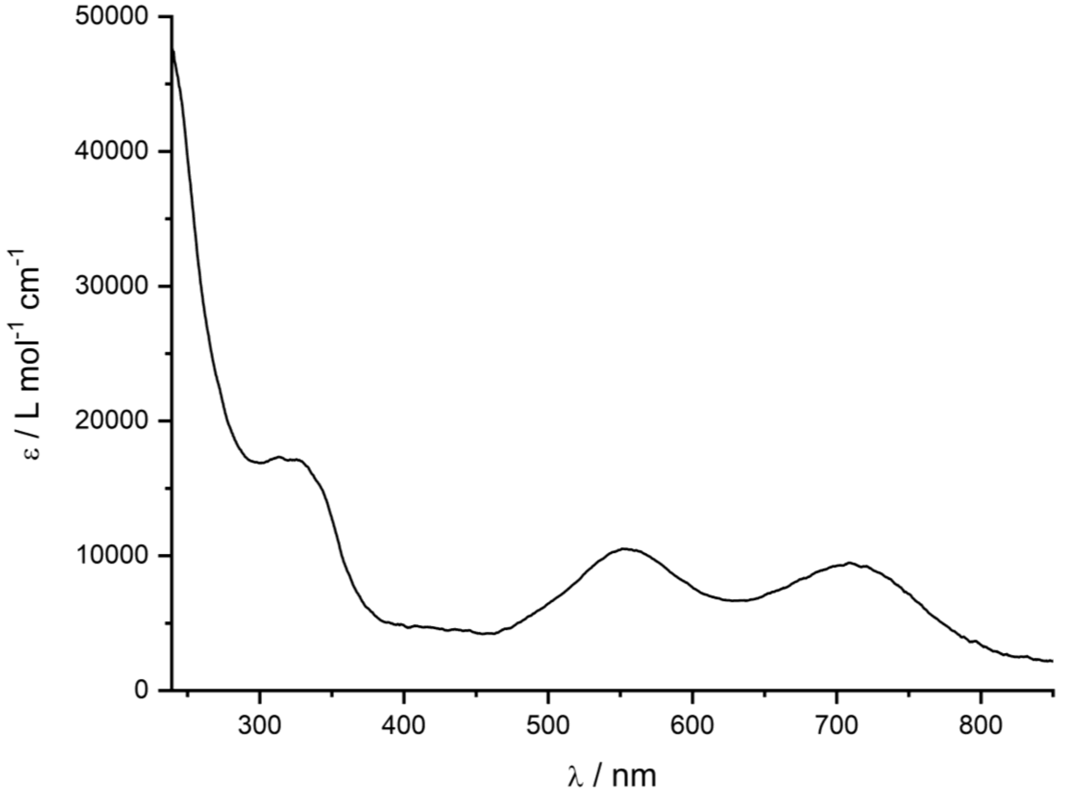

Figure S27. UV/Vis spectrum of [K(18c-6)][(Ar*BIAN)Co(η^3:η^1-$P_4SN_2(SiMe_3)_2$)] ([K(18c-6)]**4**) recorded in THF.

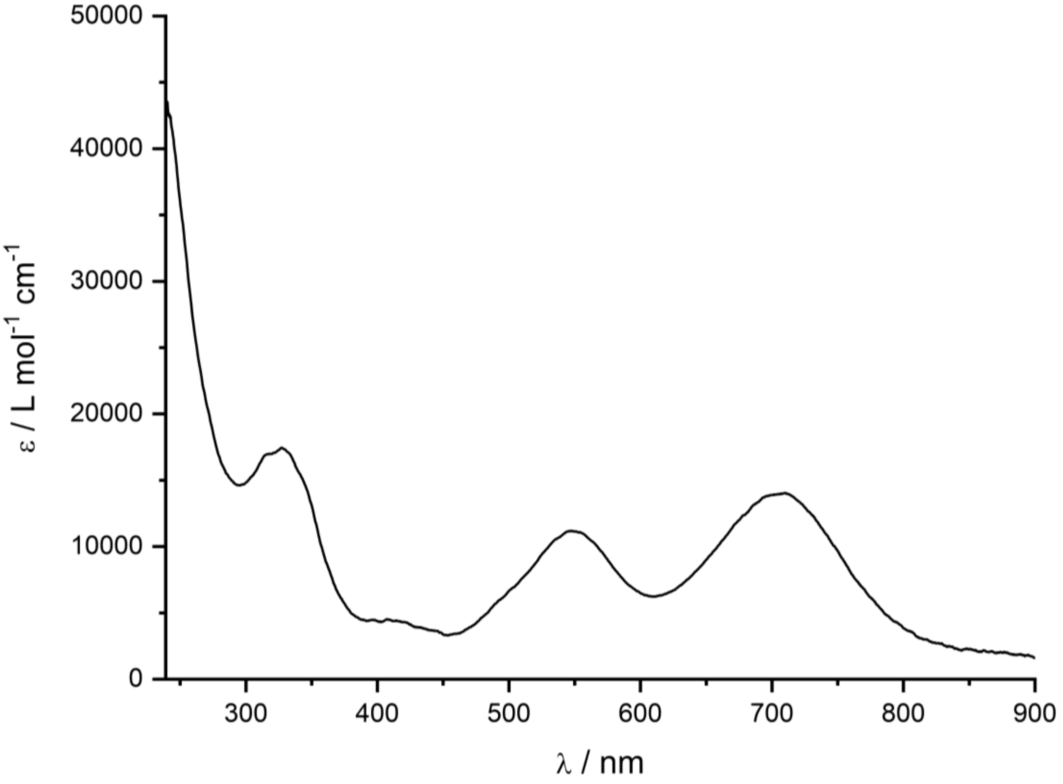

Figure S28. UV/Vis spectrum of [(Ar*BIAN)Co(η^3:η^1-$P_4SN_2(SiMe_3)_3$)] (**5**) recorded in THF.

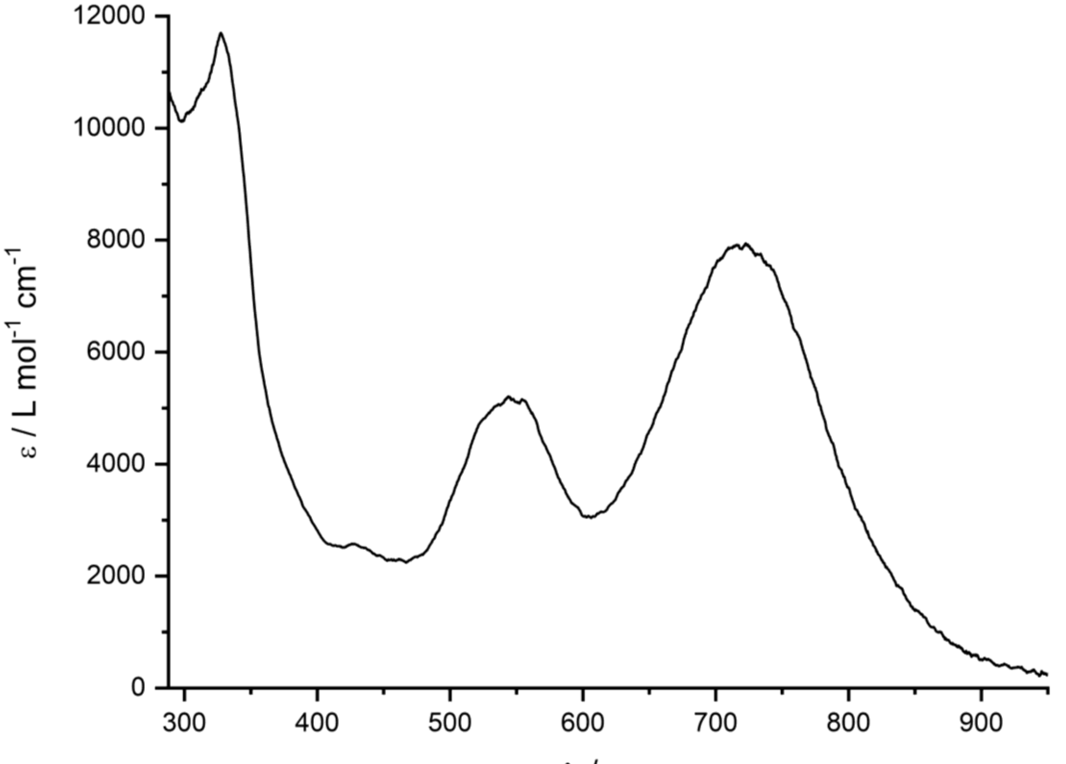

Figure S29. UV/Vis spectrum of [(Ar*BIAN)Co(η^3:η^1-$P_4C(S)N(Cy)C(O)$*t*Bu)] (**6a**) recorded in toluene.

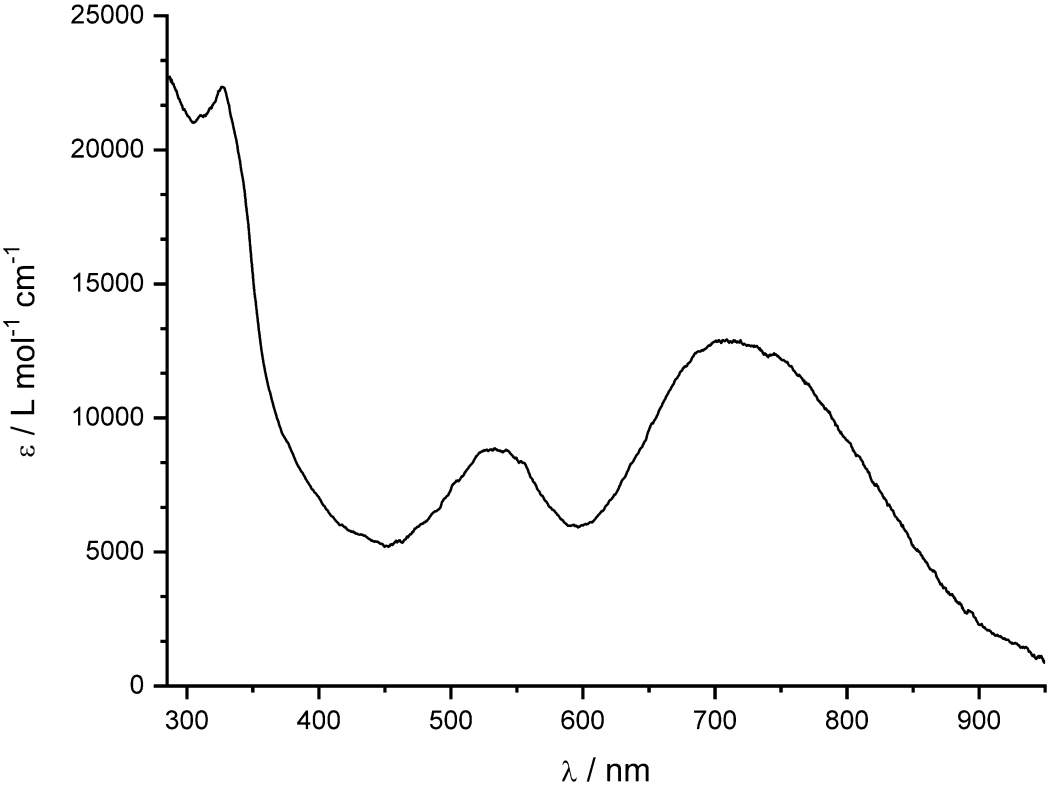

Figure S30. UV/Vis spectrum of [(Ar*BIAN)Co(η^3:η^1-P_4C(S)N(Ph)C(O)*t*Bu)] (**6b**) recorded in toluene.

IR spectra

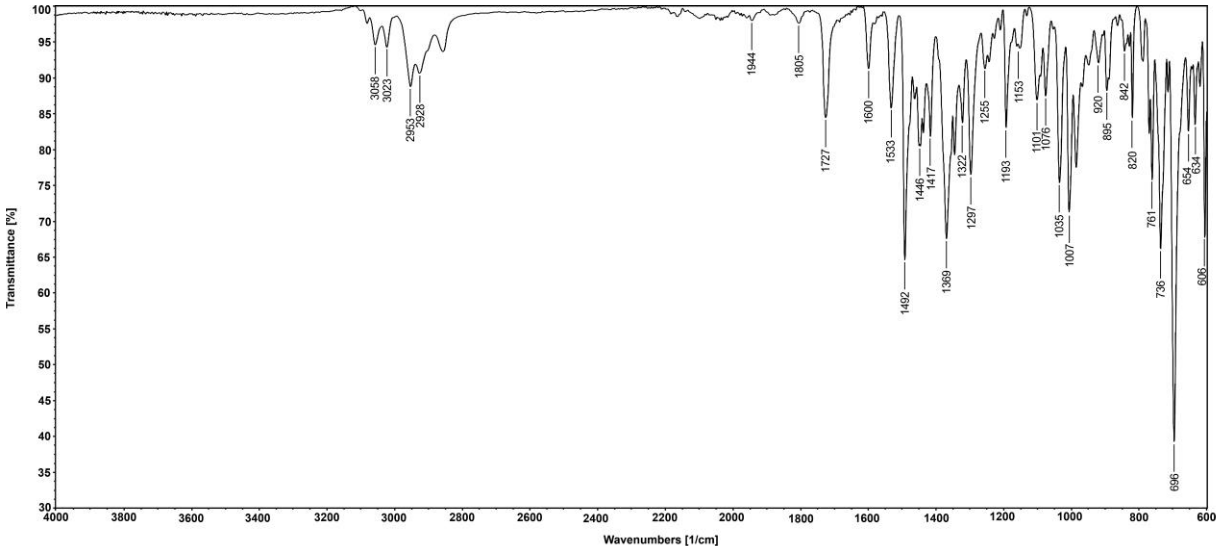

Figure S31. Solid state IR spectrum of [(Ar*BIAN)Co(η^3:η^1-P_4C(S)N(Cy)C(O)*t*Bu)] (**6a**).

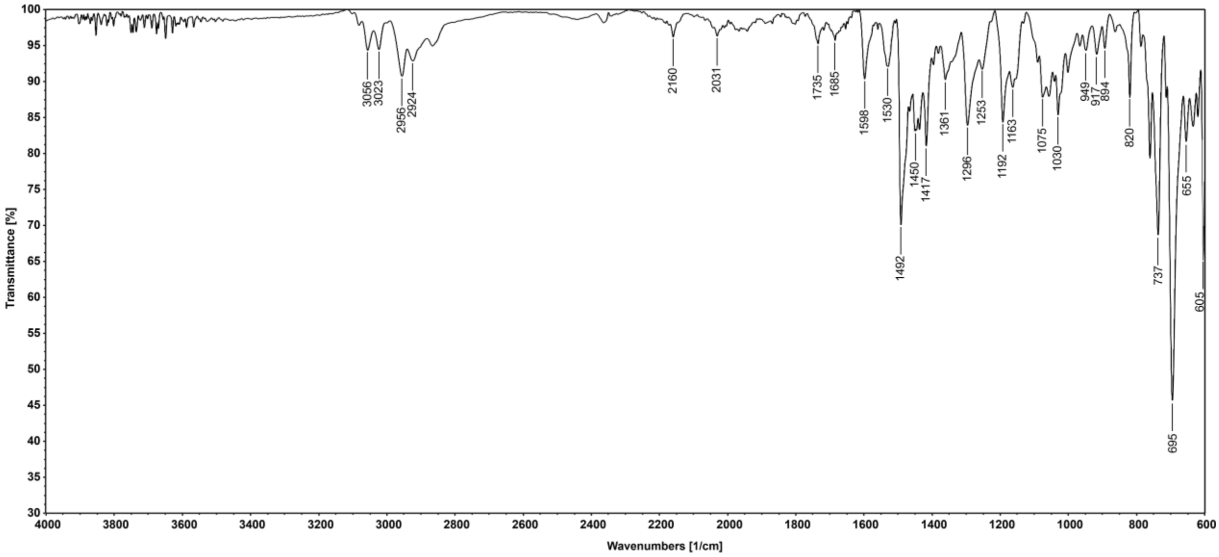

Figure S32. Solid state IR spectrum of [(Ar*BIAN)Co(η^3:η^1-P_4C(S)N(Ph)C(O)*t*Bu)] (**6b**).

X-Ray crystallography

The single-crystal X-ray diffraction data were recorded on Rigaku Oxford Diffraction SuperNova Atlas (TitanS2) or XtaLAB Synergy DW (HyPix-Arc 150) diffractometers with Cu-Kα radiation (λ = 1.54184 Å). The solid state structures for [K(18c-6)]**4** and **5** were measured at the European Synchrotron BM20 (λ = 0.56385).[1] Crystals were selected under mineral oil, mounted on micro mount loops and quench-cooled using an open flow N_2 cooling device. Either semi-empirical multi-scan absorption corrections[2] or analytical ones[3] were applied to the data. The structures were solved with SHELXT[4] solution program using dual methods and by using Olex2 as the graphical interface.[5] The models were refined with ShelXL[6] using full matrix least squares minimization on F2.[7] The hydrogen atoms were located in idealized positions and refined isotropically with a riding model.

[K(18c-6)]**4**: The crystal of [K(18c-6)]**4** contained two severely disordered toluene molecules per asymmetric unit. They were refined by using the solvent mask command: A solvent mask was calculated and 376 electrons were found in a volume of 1704 Å^3 in one void per unit cell. This is consistent with the presence of 2.0 toluene per Asymmetric Unit which account for 400 electrons per unit cell. As [K(18c-6)]**3** was crystallized by slow diffusion of *n*-hexane into a saturated toluene solution of [K(18c-6)]**4** this serves as evidence for the presence of toluene in this position. One molecule of toluene is clearly visible in vicinity of the crown ether but could not be properly modelled due to severe disorder.

5: The crystal of **5** contained 1.75 *n*-hexane molecules per in two voids per asymmetric unit. They were refined by using the solvent mask command: A solvent mask was calculated and 169 electrons were found in a volume of 924 Å^3 in two voids per unit cell. This is consistent with the presence of 0.75 *n*-hexane and 1.0 *n*-hexane per Formula Unit which account for 175 electrons per unit cell. As **5** was crystallized from *n*-hexane this serves as evidence for the presence of *n*-hexane in these positions, though they proved to be severely disordered.

6b: The crystal of **6b** contained 1.5 severely disordered toluene molecules and 1 severely disordered *n*-hexane molecules per asymmetric unit. They were refined by using the solvent mask command: A solvent mask was calculated, and 268 electrons were found in a volume of 1313 Å^3 in two voids per unit cell. This is consistent with the presence of 1.5 molecules toluene and 1.0 molecule of n-*h*exane per Formula Unit which account for 250 electrons per unit cell. As **6b** was crystallized from a mixture slow diffusion of *n*-hexane and concentrated toluene solution of **6b** this serves as evidence for the presence of toluene and *n*-hexane in these positions, though they

Table S6. Crystallographic data and structure refinement for compounds **1-5**.

Compound	[K(18c-6)]3	[K(18c-6)]4	5	6a	6b
CCDC	2279508	2325202	2279478	2279472	2279703
Empirical formula	$C_{102}H_{100}CoKN_2O_6P_4S_2$	$C_{114}H_{126}CoKN_4O_6P_4SSi_2$	$C_{91}H_{95}CoN_4P_4SSi_3$	$C_{216}H_{208}Co_2N_6O_2P_8S_2$	$C_{188}H_{164}Co_2N_6O_2P_8S_2$
Formula weight	1735.86	1958.33	1543.84	3349.61	2968.98
Temperature/K	123.0(1)	100.0(1)	100.0(1)	123.0(1)	294.0(3)
Crystal system	triclinic	monoclinic	triclinic	monoclinic	triclinic
Space group	*P-1*	$P2_1/c$	*P-1*	$P2_1/c$	*P-1*
a/Å	13.3668(6)	18.4952(7)	12.2911(3)	30.2915(4)	13.8753(3)
b/Å	14.1612(7)	21.7710(3)	13.8135(4)	23.9570(2)	23.4656(6)
c/Å	26.4289(10)	33.4049(12)	27.6744(6)	27.2607(4)	27.8232(3)
α/°	91.088(3)	90	88.140(2)	90	86.0020(10)
β/°	102.650(3)	128.156(6)	80.963(2)	116.0843(17)	86.5260(10)
γ/°	114.462(4)	90	83.427(2)	90	82.975(2)
Volume/$Å^3$	4409.1(4)	10576.8(9)	4609.3(2)	17768.0(4)	8956.7(3)
Z	2	4	2	4	2
ρ_{calc}g/cm^3	1.308	1.230	1.112	1.252	1.101
μ/mm^{-1}	3.523	0.191	0.193	2.814	2.734
F(000)	1824.0	4144.0	1628.0	7072.0	3112.0
Crystal size/mm^3	0.307 × 0.202 × 0.08	0.2 × 0.1 × 0.1	0.4 × 0.1 × 0.05	0.279 × 0.147 × 0.12	0.24 × 0.2 × 0.12
Diffractometer	SuperNova, Atlas	Synchrotron BM20, Pilatus 2M	Synchrotron BM20, Pilatus 2M	SuperNova, TitanS2	Synergy DW, HyPix-Arc 150
Radiation	Cu Kα (λ = 1.54184)	Synchrotron (λ = 0.56356)	Synchrotron (λ = 0.56385)	Cu Kα (λ = 1.54184)	Cu Kα (λ = 1.54184)
2Θ range for data collection/°	7.436 to 147.114	2.872 to 39.346	3.09 to 39.366	6.946 to 133.7	3.802 to 134.16
Index ranges	−16 ≤ h ≤ 16, −17 ≤ k ≤ 13, −32 ≤ l ≤ 32	−22 ≤ h ≤ 22, −26 ≤ k ≤ 26, −39 ≤ l ≤ 39	−14 ≤ h ≤ 14, −16 ≤ k ≤ 16, −33 ≤ l ≤ 33	35 ≤ h ≤ 36, −28 ≤ k ≤ 27, −32 ≤ l ≤ 29	−16 ≤ h ≤ 16, −28 ≤ k ≤ 28, −20 ≤ l ≤ 33
Reflections collected	30775	173474	80554	161149	111416
Independent reflections	17336 [R_{int} = 0.0751, R_{sigma} = 0.1100]	18869 [R_{int} = 0.0731, R_{sigma} = 0.0355]	16392 [R_{int} = 0.01258, R_{sigma} = 0.0945]	31309 [R_{int} = 0.0411, R_{sigma} = 0.0277]	31704 [R_{int} = 0.0765, R_{sigma} = 0.0785]
Data/restraints/para-meters	16938/3/1091	18869/2086/1357	16392/93/1005	31309/66/2154	31704/209/1925
Goodness-of-fit on F^2	1.023	1.064	1.079	1.021	1.013
Final R indexes [I>=2σ (I)]	R_1 = 0.0812, wR_2 = 0.2011	R_1 = 0.0741, wR_2 = 0.2131	R_1 = 0.0818, wR_2 = 0.1648	R1 = 0.0436, wR2 = 0.1105	R_1 = 0.0856, wR_2 = 0.2506
Final R indexes [all data]	R_1 = 0.1305, wR_2 = 0.2373	R_1 = 0.1047, wR_2 = 0.2366	R_1 = 0.1119, wR_2 = 0.1779	R_1 = 0.0538, wR_2 = 0.1180	R_1 = 0.1331, wR_2 = 0.2816
Largest diff. peak/hole / e Å^{-3}	0.8/−0.54	0.67/−0.55	1.27/−0.30	0.67/−0.59	1.11/−0.49
Flack parameter	/	/	/	/	/

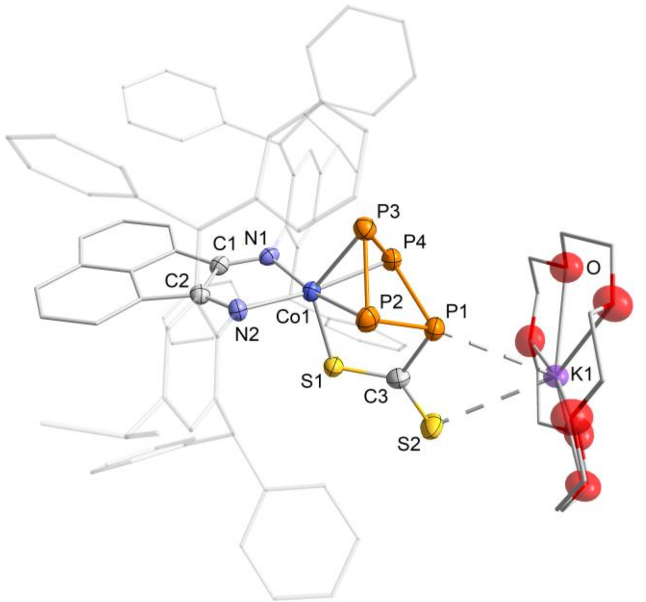

Figure S33. Solid-state molecular structure of [K(18c-6)][(Ar*BIAN)Co(η^3:η^1-P_4CS_2)] ([K(18c-6)]**3**) shown at the 50% probability level. Hydrogen atoms, non coordinating solvent molecules and disorder in the Ar*BIAN ligand omitted for clarity. Selected bond lengths [Å] and angles [°]: P1−P2 2.286(2), P2−P3 2.169(2), P3−P4 2.1697(2), P1−P4 2.227(2), Co1−P2 2.2935(1), Co1−P3 2.3031(2), Co1−P4 2.2814(2), Co1−S1 2.2725(1), Co1−N1 1.988(4), Co1−N2 1.976(4), C1−N1 1.316(6), C2−N2 1.330(6), C1−C2 1.444(7), P1−C3 1.850(6), C3−S1 1.698(5), C3−S2 1.664(5), P1-P2-P3 88.80(8), P2-P3-P4 85.07(7), P3-P4-P1 88.94(7), P4-P1-P2 82.43(6), S1-C3-S2 126.2(4), Co1-S1-C3 106.36(2).

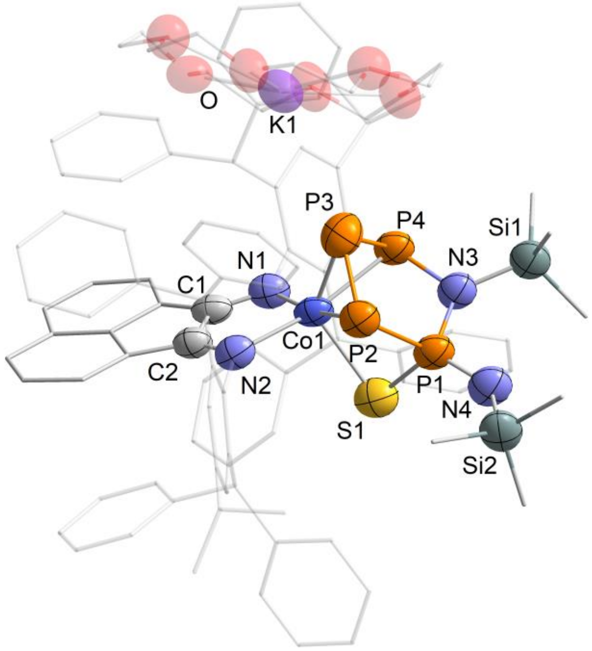

Figure S34. Solid-state molecular structure of [K(18c-6)][(Ar*BIAN)Co(η^3:η^1-$P_4SN_2(SiMe_3)_2$)] ([K(18c-6)]**4**) shown at the 50% probability level. The central CoP_4N_2S framework is disordered with its enantiomer. Hydrogen atoms, non coordinating solvent molecules and disorder are omitted for clarity. Selected bond lengths [Å] and angles [°]: P1−P2 2.205(2), P2−P3 2.047(2), P3−P4 2.200(8), P1−N3 1.681(4), P4−N3 1.749(5), P1−S1 2.049(2), P1−N4 1.567(5), N3−Si1 1.757(6), N4−Si2 1.681(5), Co1−P2 2.336(2), Co1−P3 2.235(2), Co1−P4 2.327(2), Co1−S1 2.391(2), Co1−N1 1.883(4), Co1−N2 2.033(4), C1−C2 1.455(5), N1−C1 1.334(5), N2−C2 1.322(5), P1-P2-P3 103.00(7), P2-P3-P4 95.57(8), P3-P4-N3 104.75(2), P4-N3-P1 109.5(3), Co1-S1-P1 81.90(8), Si1-N3-P1 124.6(3), Si2-N4-P1 134.0(3), N1-Co1-N2 83.12(2).

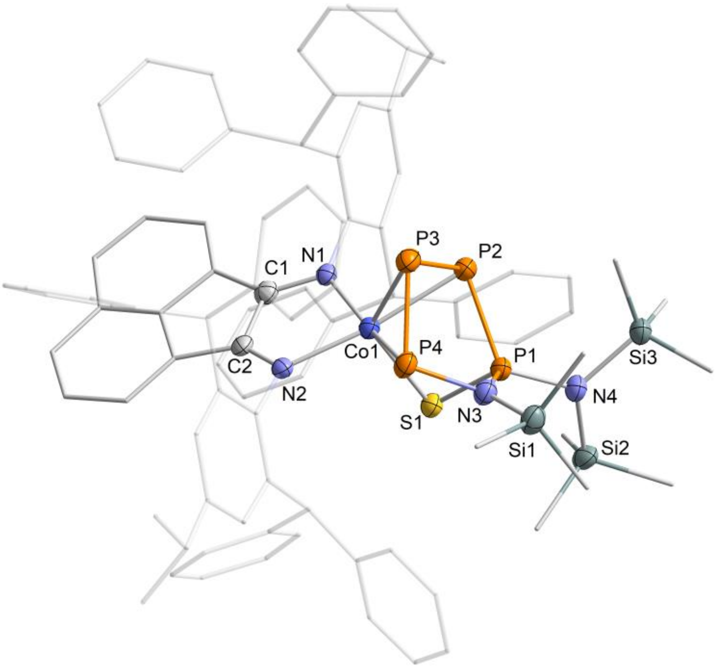

Figure S35. Solid-state molecular structure of [(Ar*BIAN)Co(η^4:η^4-$P_4SN_2(SiMe_3)_3$)] (**5**) shown at the 50% probability level. Hydrogen atoms, non coordinating solvent molecules and disorder in the Ar*BIAN ligand omitted for clarity. Selected bond lengths [Å] and angles [°]: P1−P2 2.1966(2), P2−P3 2.1451(2), P3−P4 2.1789(2), Co1–P2 2.3298(1), Co1–P3 2.3210(1), Co1–P4 2.2935(1), Co1–S1 2.3416(1), P1–N3 1.670(4), N3–P4 1.782(4), N3–Si1 1.768(4), P1–N4 1.666(4), N4–Si2 1.813(4), N4–Si3 1.791(4), Co1–N1 1.970(4), Co1–N2 1.986(3), C1–C2 1.457(6), P1-P2-P3 100.50(6), P2-P3-P4 94.04(6), P3-P4-N3 104.90(1), P4-N3-P1 106.3(2), Co1-S1-P1 81.03(5), Si1-N3-P1 134.7(2), Si2-N4-Si3 120.8(2), N1-Co1-N2 83.11(1).

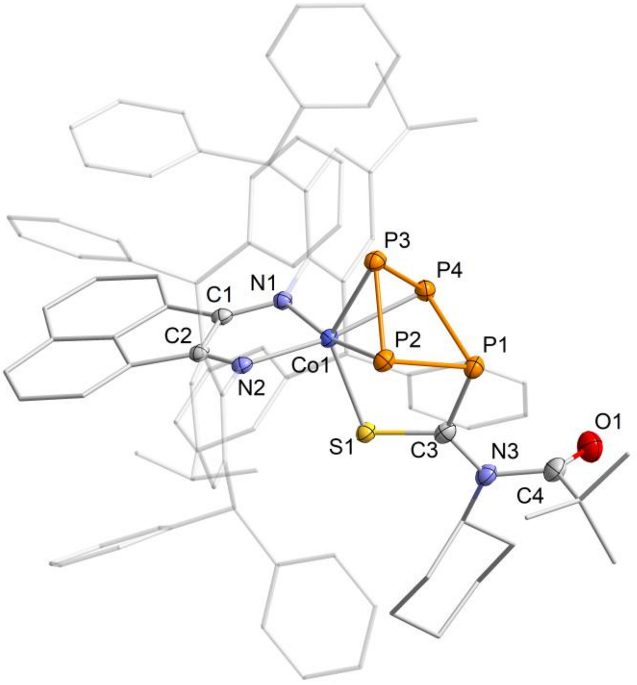

Figure S36. Solid-state molecular structure of [(Ar*BIAN)Co(η^3:η^1-$P_4C(S)N(Cy)C(O)tBu$)] (**6a**) shown at the 50% probability level. Hydrogen atoms and non coordinating solvent molecules omitted for clarity. The asymmetric unit contained a second crystallographically independent molecule which is not shown. Selected bond lengths [Å] and angles [°]: P1−P2 2.2437(8), P2−P3 2.1697(7), P3−P4 2.1669(8), P1−P4 2.2360(7), Co1−P2 2.2881(5), Co1−P3 2.2915(6), Co1−P4 2.2838(8), Co1−S1 2.2583(6), Co1−N1 1.9701(2), Co1−N2 1.9693(2), C1−N1 1.311(3), C2−N2 1.316(3), C1−C2 1.459(3), P1−C3 1.856(2), C4−O1 1.196(3), C3−N3 1.343(3), C4−N3 1.471(3), P1−P2−P3 88.80(3), P2-P3-P4 85.38(3), P3-P4-P1 89.07(3), P4-P1-P2 82.05(3), Co1-S1-C3 104.69(8), C3-N3-C4 118.70(2).

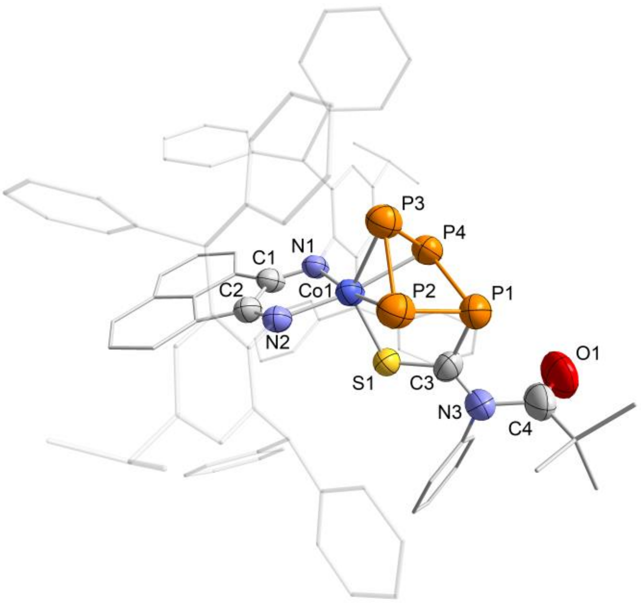

Figure S37. Solid-state molecular structure of [(Ar*BIAN)Co(η^3:η^1-P_4C(S)N(Ph)C(O)*t*Bu)] (**6b**) shown at the 50% probability level. Hydrogen atoms, and disorder in the *t*Bu-group omitted for clarity. The asymmetric unit contained a second crystallographically independent molecule which is not shown. Selected bond lengths [Å] and angles [°]: P1−P2 2.240(2), P2−P3 2.163(2), P3−P4 2.169(2), P1−P4 2.2339(2), Co1−P2 2.2818(2), Co1−P3 2.2994(2), Co1−P4 2.2851(2), Co1−S1 2.2597(1), Co1−N1 1.976(3), Co1−N2 1.984(4), C1−N1 1.295(6), C2−N2 1.325(5), C1−C2 1.446(6), P1−C3 1.861(6), C4−O1 1.211(8), C3−N3 1.343(7), C4−N3 1.462(7), P1−P2−P3 89.20(8), P2-P3-P4 84.96(8), P3-P4-P1 89.20(8), P4-P1-P2 81.68(7), Co1-S1-C3 104.8(2), C3-N3-C4 119.2(5).

Proposed reaction mechanism for insertion of isothiocyanates

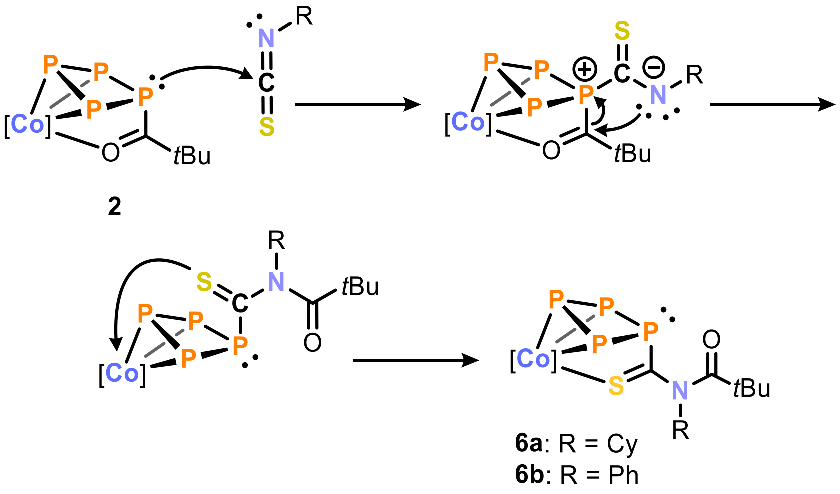

Scheme S1. Proposed reaction mechanism for insertion reaction of isothiocyanates RNCS (R = Cy, Ph) in P−C bonds of [(Ar*BIAN)Co(η^3:η^1-P_4C(O)*t*Bu)] (**2**) yielding **6a** and **6b**.

Quantum chemical calculations

All calculations were performed with the ORCA 5.0 program.[8] The geometry was optimized starting from the X-ray coordinates at the TPSS[9]/def2-TZVP[10] level. Dispersion correction was included via the Grimme's D4 model,[11] and the solvent effects by using the CPCM model.[12] The Intrinsic Bonding Orbitals[13] were calculated as implemented in ORCA and visualized using ChemCraft.[14]

Calculation of the ^{31}P NMR chemical shifts

The geometry of the molecules has been optimized using the PBE[15] functional together with the def2-SVP basis set for C and H and def2-TZVP for all other atoms, using tight convergence criteria. The dispersion correction has been incorporated via the D3BJ[16] scheme and the solvent effects by using the CPCM model with the dielectric constant of benzene. The ^{31}P chemical shifts has been calculated using the GIAO[17] formalism as single point calculations with the PBE0[18] functional using the aug-pcSseg-2 basis set[19] (taken from the Basis Set Exchange library[20]) for phosphorus and the def2-TZVP basis set for all other atoms. In case of the hybrid functional PBE0 the RIJCOSX approximation has been used.[21] The calculated absolute shifts has been referenced to the absolute shift of 85% H_3PO_4 using PH_3 as a secondary standard (δ_{PH3} in C_6D_6 = −240 ppm) by using the equation: $\delta_{calc,X} = \sigma_{calc,PH3} - \sigma_{calc,X} - 240$ ppm.

Table S7. Calculated (at the D3BJ-PBE0/def2-TZVP/aug-pcSseg-2 @P/CPCM level of theory) and experimental ^{31}P NMR chemical shifts of [(Ar*BIAN)Co(η^3:η^1-P_4C(S)N(Cy)C(O)*t*Bu)] (**6a**).

	calculated	experimental (at 213K)
P3	91	85.5
P4	123	105.4
P5	125	117.9
P6	110	77.6

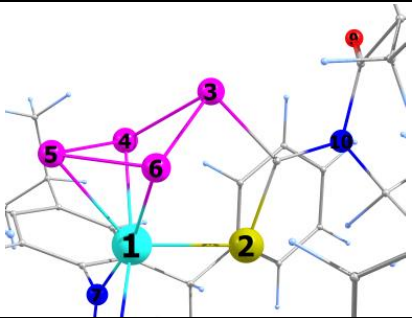

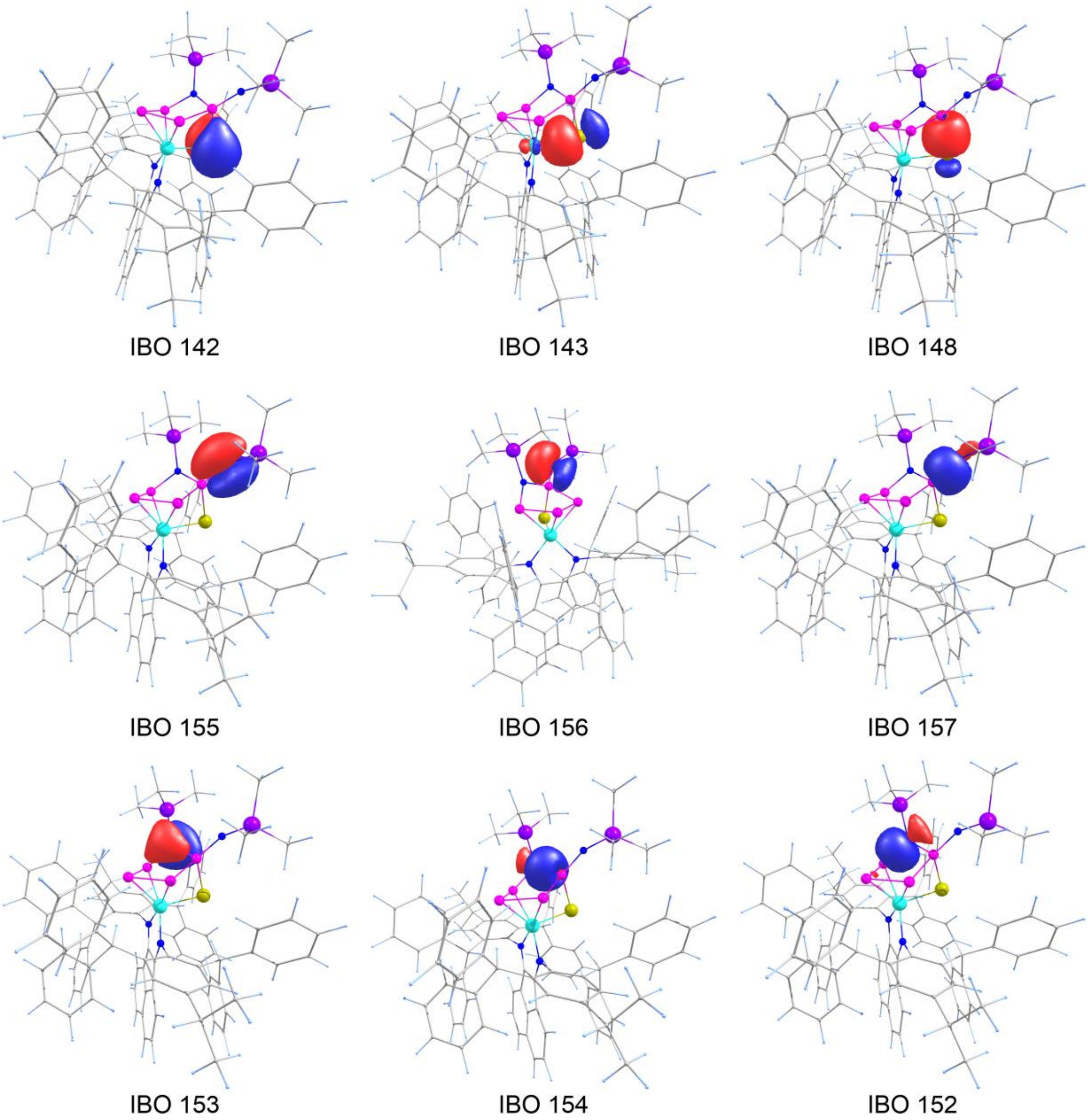

Figure S38. Selected Intrinsic Bonding Orbitals of $[(\mathrm{Ar^{*}BIAN})\mathrm{Co}(\eta^{3}{:}\eta^{1}\text{-}\mathrm{P_4SN_2(SiMe_3)_2})]^{-}$ (**4**$^{-}$) at the TPSS-D4/def2-TZVP CPCM(THF) level of theory.

Selected IBOs

```
MO 158:  10Si -   0.336766  and   8N  -   0.668073
MO 157:   9N  -   0.579533  and   5P  -   0.422175
MO 156:   9N  -   0.840853  and   5P  -   0.079905
MO 155:   9N  -   0.754930  and   5P  -   0.142491
MO 154:   8N  -   0.576713  and   5P  -   0.404438
MO 153:   8N  -   0.848485  and   5P  -   0.050514
MO 152:   8N  -   0.597646  and   2P  -   0.363415
MO 151:   7N  -   0.861723  and   0Co -   0.192947
MO 150:   6N  -   0.883734  and   0Co -   0.186604
MO 149:   5P  -   0.575444  and   4P  -   0.471926
MO 148:   5P  -   0.445004  and   1S  -   0.576771
MO 147:   4P  -   0.518845  and   3P  -   0.490022
MO 146:   4P  -   0.519738  and   0Co -   0.423037
MO 145:   3P  -   0.526900  and   2P  -   0.492273
MO 144:   2P  -   0.555703  and   0Co -   0.424569
MO 143:   1S  -   0.717700  and   0Co -   0.162893
MO 142:   1S  -   0.920644  and   0Co -   0.012502
```

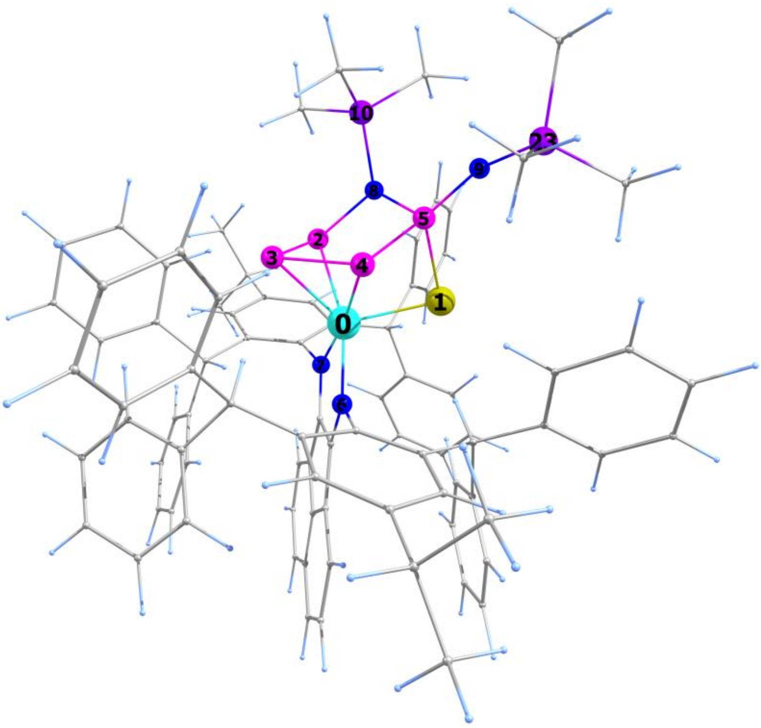

Figure S39. Optimized geometry of [(Ar*BIAN)Co(η^3:η^1-$P_4SN_2(SiMe_3)_2$)]$^-$ (**4**$^-$) at the TPSS-D4/def2-TZVP CPC(THF) level of theory and atom labeling.

Cartesian coordinates of the optimized geometry of [(Ar*BIAN)Co(η^3:η^1-$P_4SN_2(SiMe_3)_2$)]$^-$ (**4**$^-$) at the TPSS-D4/def2-TZVP CPC(THF) level of theory.

Co	0.12269356182392	0.03991539174372	-0.13889055865780
S	0.84479587300004	0.70002707086195	-2.22766303871057
P	-0.88606923754045	-1.78612115898880	-1.04896118972262
P	-2.01200657282663	-0.58398514621266	0.38497795984413
P	-1.75020275403189	1.22751560740456	-0.75261021887770
P	-1.15064602338650	0.45278294418441	-2.74203118573080
N	0.76167137813099	1.39614444767224	1.12139684371542
N	1.57856146334266	-1.00047295723704	0.60683032464767
N	-1.40224592359920	-1.22027605899079	-2.62204451323577
N	-1.68623245955753	0.97312815055237	-4.10823310259208
Si	-2.79769524983070	-1.97701436244346	-3.41777109865314
C	-4.37156968466898	-1.06472598032021	-2.94473019272394
H	-4.35455950920002	-0.03767999951167	-3.32398760488712
H	-4.48067425935328	-1.02444706530140	-1.85442876917244
H	-5.25402453115864	-1.56992503454872	-3.35783614294479
C	-2.58058706890929	-2.00498280959929	-5.28008717615475
H	-3.45890459787620	-2.46597012374279	-5.75066044890539
H	-1.69988756543358	-2.59041011171975	-5.56668721449442
H	-2.46384211210174	-0.99034383708004	-5.66919052558307
C	-2.88278220332523	-3.74943983058860	-2.79169498492441
H	-3.11431728032464	-3.80318300287393	-1.72239747966050
H	-1.93804645845318	-4.27973472427433	-2.96005840328807
H	-3.67221746904939	-4.28462779548853	-3.33646683683761
Si	-2.32086730726717	2.39413167600414	-4.79209156571552
C	-3.15877459076805	3.50492738230990	-3.51298610457000
H	-4.01497945348585	2.98852899852379	-3.06055719598702
H	-3.52624998988070	4.43492324411170	-3.96647300876870
H	-2.47157645134069	3.77017491623192	-2.70216194854255
C	-3.62447063118935	1.90969535421571	-6.07174676481380
H	-3.18986376138083	1.27680437611245	-6.85580759704197
H	-4.05803776108396	2.79418652133517	-6.55698216943308
H	-4.44291418257556	1.34663268709386	-5.60562079117012
C	-0.96987835387333	3.35973051405474	-5.69119957635060
H	-0.15443618085484	3.62675507417076	-5.01003010269327
H	-1.35516501190587	4.28677604527343	-6.13578152074413

H	-0.54582207453102	2.75022299857890	-6.49971084424867
C	1.86683098903642	1.02023755055618	1.74545919747685
C	0.28920153814980	2.71707281759608	1.40962902575579
C	2.32510278528900	-0.31516239797778	1.45616868313277
C	1.76676240236386	-2.41186846668885	0.48599541428637
C	2.76375767862345	1.61127201241576	2.73283542961024
C	-0.70859878313960	2.92808679496505	2.38011719483269
C	0.84936990952190	3.81023985193477	0.72208808867120
C	3.52640528177855	-0.57365674886553	2.23728542114721
C	2.38130900595660	-2.97628984125966	-0.64452111076726
C	1.20647290685159	-3.24188113611826	1.47844481156767
C	3.74784229877739	0.61549620982097	2.98352507185204
C	2.85323210328663	2.81393412938887	3.40596823837747
C	-1.46578099256443	1.77489950636365	3.02644423922870
C	-1.04142165184638	4.24424314432655	2.71442903322689
C	1.85395309989637	3.58984155961918	-0.40112372259290
C	0.47547637923227	5.10299255956962	1.08344777544474
C	4.39760717925930	-1.63263621158022	2.39823496947200
C	2.98116030887036	-2.08993158777164	-1.72299981876452
C	2.42984658278611	-4.36627313415159	-0.76004222220025
C	0.56576819959185	-2.66509750606615	2.73343999825173
C	1.25100531463408	-4.62513077961637	1.30048132232229
C	4.81036822924662	0.78052770538584	3.88261954811193
H	2.14323803398474	3.61557189175779	3.23665116120234
C	3.91331454070753	2.99595251205991	4.32960847279746
C	-0.88067857524053	1.24804317780954	4.32535359178882
H	-1.45016582437758	0.94414312747988	2.30678093047279
C	-2.93429311663525	2.15961515138685	3.23296754502115
H	-1.80867399503144	4.41332526015408	3.46585900655162
C	-0.44744377857210	5.34501196542718	2.10097131285011
C	1.63082958628788	4.57067534703751	-1.54606623804201
H	1.65302843692006	2.59081717238605	-0.80765504152135
C	3.27385115563451	3.57558791856956	0.13623112701944
H	0.90386548372579	5.93803038510995	0.53538025005485
C	5.48948317078924	-1.47908106367296	3.28721813165616
H	4.25389640146436	-2.56932216033692	1.87405705566447
H	2.37707462126012	-1.17447937467016	-1.74682862919598
C	4.41593554772173	-1.67973342430052	-1.43531904765400
C	2.85181700093516	-2.72394641208731	-3.10405179410171
H	2.89708968507164	-4.80141479791995	-1.64016804860977
C	1.86338707067757	-5.21112594917571	0.19382111786780
C	-0.74038558592536	-3.37200663353017	3.09366664490501
C	1.56324036510895	-2.64749132637428	3.88770860707343
H	0.29844682597628	-1.62273724903003	2.51769110687015
H	0.76588193268252	-5.25544132497862	2.04123632376745
C	4.86868052206779	2.02335626435291	4.57087111332140
C	5.70348961905808	-0.31795140413970	4.01257327649106
H	3.97981617688098	3.94381293252100	4.85705112462673
C	0.03152805694308	1.96945325591725	5.09552714499931
C	-1.37056738538857	0.03139435574132	4.82380526427359
C	-3.43804217022165	2.52631223387182	4.48611982187506
C	-3.80031739418711	2.18902659459452	2.13359294866922
C	-0.83887696757911	6.75882576397619	2.48482738825935
C	0.32644792015392	4.76839483783675	-2.02303443655970
C	2.67715709033526	5.25348082072312	-2.17169862214522
C	4.06557412834454	2.43880071733138	-0.03202853953927
C	3.80033307499746	4.66382216482837	0.84170376213445
H	6.17540704244034	-2.31274909834305	3.41065252484182
C	5.29788148198434	-2.48399767551616	-0.70925664729638
C	4.89603579644057	-0.48436882290841	-1.98523421196831
C	3.91461068823776	-3.38070104999715	-3.73281114200399
C	1.61406720304794	-2.66793743010540	-3.75691330063201
C	1.88226261339648	-6.71665465221698	0.01323379440595
C	-1.07371034584772	-3.68571988845618	4.41401998599152
C	-1.66829156317852	-3.66376812868338	2.08451907796643
C	2.27271641740629	-3.79702476315118	4.25565559196267
C	1.77971305021178	-1.47234420855757	4.60846841217971
H	5.66815342335762	2.20916602743064	5.28396566887296
H	6.54851241971582	-0.24873239557353	4.69316469991384

H	0.41599629632846	2.91338193489794	4.72444796852054
C	0.43668188407370	1.49872682832281	6.34757468227072
H	-2.07247170096419	-0.54442995332827	4.22558076750736
C	-0.98564466440678	-0.42971707583991	6.07860689675644
H	-2.78527798023388	2.50983557183605	5.35392287505689
C	-4.77170640580610	2.91273815964566	4.63675442373340
H	-3.42021619103197	1.91647741021113	1.15227833253070
C	-5.13377039279292	2.56701036444202	2.27965076617571
H	-1.54068265286402	6.68467637771642	3.32568329625067
C	0.38092728735601	7.57443013537040	2.94768997711809
C	-1.55982185194987	7.46601988368548	1.32247480823462
H	-0.49467652700542	4.23419625902011	-1.55270070528835
C	0.07220015328601	5.64408706717603	-3.07422434118352
H	3.69653131184715	5.10705930905692	-1.82746407225554
C	2.42702360435213	6.12892908819647	-3.23294934359819
H	3.66453760273329	1.58800008099345	-0.57607335421880
C	5.34330074761814	2.36871065054528	0.52447839324573
H	3.19527619883296	5.55490949589180	0.98524419448422
C	5.08417253364919	4.60866734845617	1.37874816598618
H	4.94161855028864	-3.41556374277222	-0.27862531131755
C	6.62947442846268	-2.10368560840868	-0.53115015756233
H	4.21804326483380	0.14477310157881	-2.55739488265452
C	6.22498637087979	-0.10239442019265	-1.81455373528953
H	4.88463234181096	-3.42259387067228	-3.24529732916891
C	3.74318671872602	-3.98240088935974	-4.98195288954532
H	0.78501502412840	-2.14805897187081	-3.28524936631279
C	1.44209414076247	-3.26312319825487	-5.00535007087598
H	2.42519920880457	-6.92468892171921	-0.91796164132218
C	2.62481333477282	-7.41484615731609	1.16612757828301
C	0.45345715283028	-7.27158216826606	-0.13241867882048
H	-0.36895652328684	-3.46870687802614	5.21025095670592
C	-2.30472770405136	-4.27070060827415	4.72217041869925
H	-1.41923655833107	-3.44042732932825	1.05000661688239
C	-2.89724759788866	-4.24529923720254	2.38662736980336
H	2.12605609313859	-4.72157559447949	3.70575744309949
C	3.17685817136680	-3.77008305054322	5.31566862348882
H	1.23937735295461	-0.57251428978942	4.33421623957755
C	2.68753539921455	-1.43777661379700	5.66664909434078
H	1.15268143227244	2.07155862674372	6.93146206442066
C	-0.08240217572150	0.30694365661504	6.84936571099277
H	-1.37845437752725	-1.37116184656779	6.45142875222190
C	-5.62668926711210	2.93333743001854	3.53443247111013
H	-5.14111874624063	3.19689245463918	5.61887747149086
H	-5.78887440946479	2.57464275880539	1.41207056818518
H	1.11235156881446	7.67049749582900	2.13682822002069
H	0.87714173221350	7.09237989317703	3.79701852260202
H	0.07669029364822	8.58289647755122	3.25066875958770
H	-1.88348413997936	8.46902278221502	1.62385670232907
H	-2.44014910609059	6.89749455238975	1.00460753477887
H	-0.89209116593386	7.56924566878940	0.45917125627653
C	1.12403378902685	6.33421822542652	-3.68335177639520
H	-0.94656258924720	5.78439991169517	-3.42370166420840
H	3.25557242157335	6.65287544854745	-3.70293508700843
H	5.92963293798837	1.46241440264212	0.40932867849542
C	5.85590657734131	3.45359294912839	1.23340450785922
H	5.47590325377920	5.45941512225486	1.93062052316315
H	7.29922478073047	-2.73945568639967	0.04222835117118
C	7.09861367369052	-0.91209665269843	-1.08353030617770
H	6.57767631221902	0.83148033814281	-2.24395197482249
H	4.57985234595423	-4.49155403823214	-5.45382423125569
C	2.50545871369069	-3.92685819887950	-5.62296652674816
H	0.47767045129051	-3.20504348019304	-5.50217655960381
H	3.65190107681446	-7.04417014019111	1.25328606479957
H	2.65995618222237	-8.49782349986586	1.00070089074107
H	2.11591260564059	-7.23420263402155	2.12013201485053
H	-0.12604884701413	-7.09029721782012	0.78033119949034
H	0.47718246140758	-8.35280933698761	-0.31107790230214
H	-0.06925519156918	-6.79228863346311	-0.96698373813244
C	-3.22246419898538	-4.55257891436466	3.71062030005028

H	-2.54344233731686	-4.50601068604078	5.75635473898498
H	-3.60201046543197	-4.45868658691952	1.58689410010987
H	3.72111463699498	-4.67249953362284	5.58290419294290
C	3.38959165268723	-2.58640902776572	6.02548968885352
H	2.84840427847618	-0.50783468536724	6.20320328474843
H	0.22598231469533	-0.05686573210703	7.82564047096066
H	-6.66621915450047	3.22792708022596	3.65138546515506
H	0.92767006962581	7.01904682590681	-4.50395219663733
H	6.84671519975256	3.39864876119587	1.67633300780840
H	8.13405001422095	-0.61314032264633	-0.94413182447121
H	2.37114150295188	-4.38972624427076	-6.59710497270153
H	-4.17973900112879	-5.00857577051287	3.94893584779469
H	4.10304825818226	-2.55997255854244	6.84502326123710

Selected Mayer bond orders

```
B(  0-Co,  1-S ) :   0.5380 B(  0-Co,  2-P ) :   0.7770 B(  0-Co,  3-P ) :   0.5890
B(  0-Co,  4-P ) :   0.6590 B(  0-Co,  6-N ) :   0.6346 B(  0-Co,  7-N ) :   0.6431
B(  0-Co, 36-C ) :   0.1026 B(  0-Co, 38-C ) :   0.1067 B(  1-S ,  5-P ) :   1.0783
B(  2-P ,  3-P ) :   1.0473 B(  2-P ,  8-N ) :   0.9456 B(  2-P , 39-C ) :   0.1375
B(  3-P ,  4-P ) :   1.1267 B(  4-P ,  5-P ) :   0.8914 B(  4-P , 37-C ) :   0.1155
B(  5-P ,  8-N ) :   1.0454 B(  5-P ,  9-N ) :   1.5552 B(  6-N , 36-C ) :   1.3603
B(  6-N , 37-C ) :   0.5935 B(  7-N , 38-C ) :   1.4323 B(  7-N , 39-C ) :   0.5138
B(  8-N , 10-Si) :   0.9672 B(  9-N , 23-Si) :   1.2503 B( 10-Si, 11-C ) :   0.9915
B( 10-Si, 15-C ) :   0.9869 B( 10-Si, 19-C ) :   1.0217 B( 11-C , 12-H ) :   0.9573
B( 11-C , 13-H ) :   0.9434 B( 11-C , 14-H ) :   0.9355 B( 15-C , 16-H ) :   0.9345
```

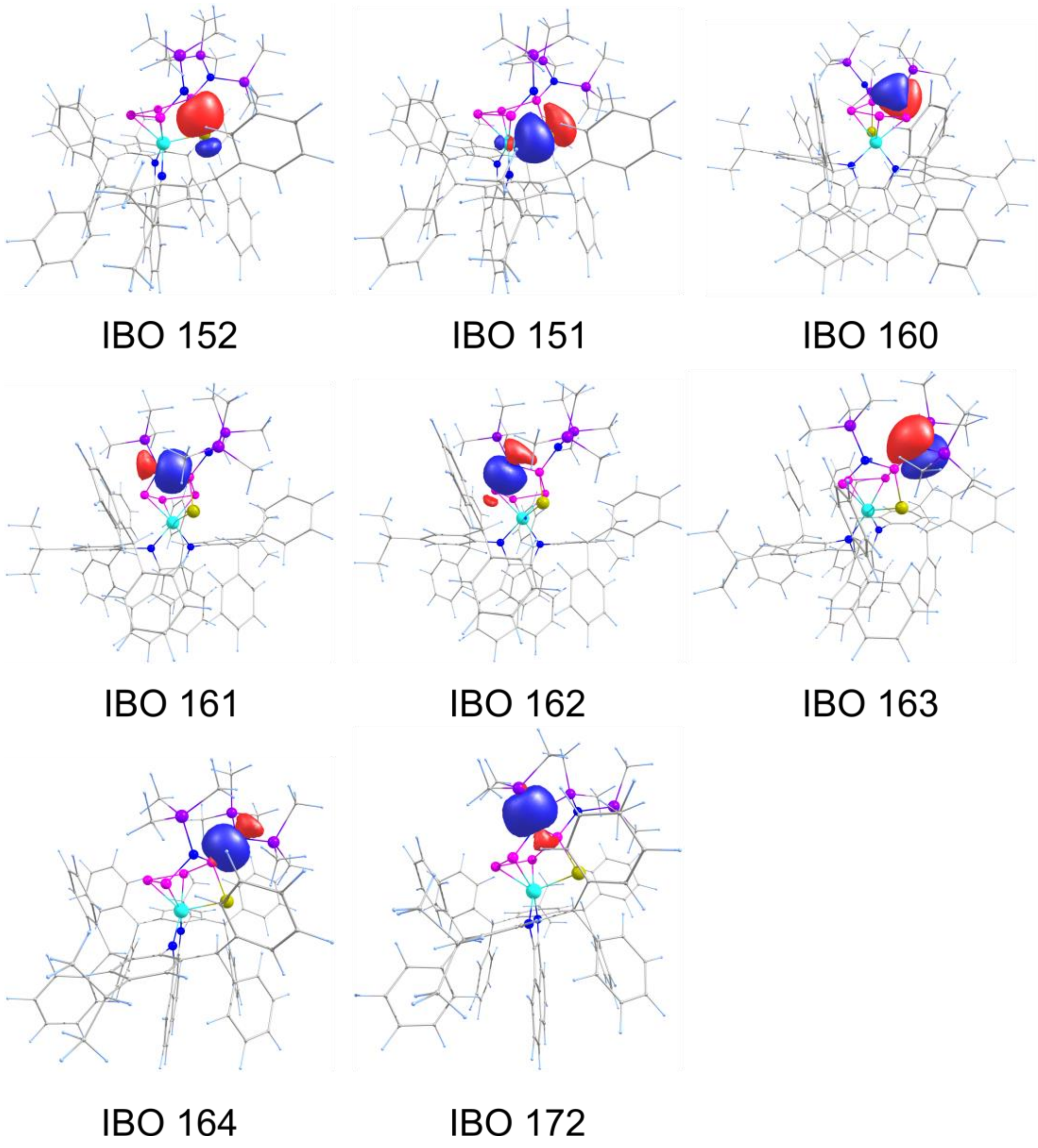

Figure S40. Selected Intrinsic Bonding Orbitals of [(Ar*BIAN)Co(η^3:η^1-$P_4SN_2(SiMe_3)_3$)] (**5**) at the TPSS-D4/def2-TZVP CPCM(THF) level.

Selected IBOs

```
MO 174:  16Si -   0.338160  and   9N  -   0.663503
MO 173:  15Si -   0.343615  and   9N  -   0.664794
MO 172:  14Si -   0.335445  and   8N  -   0.672070
MO 164:    9N  -   0.591659  and   2P  -   0.393448
MO 163:    9N  -   0.805886  and   2P  -   0.080942
MO 162:    8N  -   0.603899  and   5P  -   0.342525
MO 161:    8N  -   0.551419  and   2P  -   0.426669
MO 160:    8N  -   0.827895  and   2P  -   0.059011
MO 159:    7N  -   0.861832  and   0Co -   0.201273
MO 158:    6N  -   0.874984  and   0Co -   0.188600
MO 157:    5P  -   0.483267  and   4P  -   0.524460
MO 156:    5P  -   0.571919  and   0Co -   0.396117
MO 155:    4P  -   0.464757  and   3P  -   0.540127
MO 154:    3P  -   0.411902  and   2P  -   0.631653
MO 153:    3P  -   0.550641  and   0Co -   0.373465
MO 152:    2P  -   0.461122  and   1S  -   0.553043
MO 151:    1S  -   0.725958  and   0Co -   0.176304
MO 150:    1S  -   0.922476  and   0Co -   0.011767
```

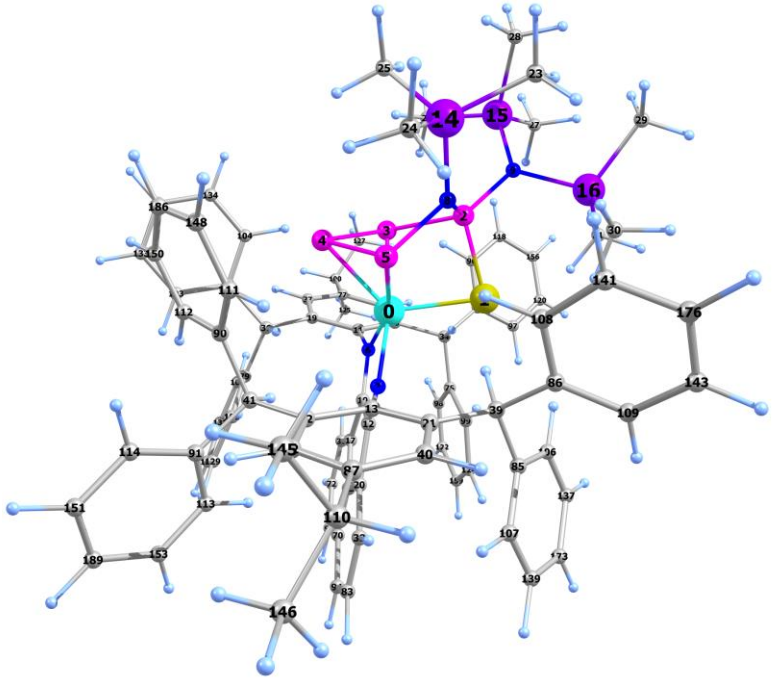

Figure S41. Optimized geometry of [(Ar*BIAN)Co(η^3:η^1-$P_4SN_2(SiMe_3)_3$)] (**5**) at the TPSS-D4/def2-TZVP CPC(THF) level and atom labeling.

Cartesian coordinates of the optimized geometry of [(Ar*BIAN)Co(η^3:η^1-$P_4SN_2(SiMe_3)_3$)] (**5**) at the TPSS-D4/def2-TZVP CPC(THF) level.

Co	-0.09491285559101	0.09500971359674	-0.06058070639845
S	-0.43758113871060	0.68105869098351	-2.26809467208034
P	1.06351249244783	-0.67327259434489	-2.52249480281810
P	2.10983498656390	0.01092835683576	-0.70917108808589
P	1.40998884037338	-1.43883335706982	0.71582167167789
P	-0.24156958314395	-2.09610730513094	-0.55371779030161
N	0.12090780909033	1.76813418781937	0.93077059467113
N	-1.84976104826616	0.09986326302084	0.77225026667929
N	0.42593744943622	-2.18805583308749	-2.19556710861261
N	1.76046545833374	-0.63470465725289	-4.03954690856217
C	-0.98770819879779	2.14410153247410	1.53285667461906
C	1.30687993094590	2.54298395393614	1.11972998887270
C	-2.09621934326781	1.22384646241014	1.42218089525790
C	-2.81767633773818	-0.95159183735568	0.89899111965175
Si	0.99699924430022	-3.80773792396743	-2.66929279885406
Si	3.52700064746981	-0.71903740663204	-4.34630417887549
Si	0.68162593518606	-0.21258686641089	-5.43237191666832
C	-1.39720322906195	3.26289456414438	2.37096328428113
C	1.66721821282111	3.51131308088877	0.16541723775578
C	2.12640449752048	2.29885581249682	2.23303416766509
C	-3.22502897601255	1.76868969691861	2.16264453328530
C	-3.71298790466434	-1.23970424881904	-0.14240726772966
C	-2.85349914727277	-1.70489561991058	2.09103687523521
C	0.90143367924085	-4.02753284215595	-4.52549343023048
C	-0.14532340563707	-5.06288772392562	-1.86641917157360
C	2.72887265121664	-4.07731673111963	-1.99906501707251
C	4.53741191234800	-0.58862876844361	-2.77282138457006
C	4.04238552557411	0.72526926331413	-5.42846652149554
C	4.00564813758730	-2.32616050262610	-5.19254765554989
C	1.34593867997946	-0.99167612294648	-7.01163838037692
C	-1.04264860391426	-0.91209712316507	-5.21713443021472
C	0.61017086404505	1.65113984705592	-5.62842126211666
C	-2.74980026323338	2.99252781547286	2.71257716386654
C	-0.79937084093615	4.40537590732842	2.86116353119793
C	0.75363726127796	3.76835567275620	-1.02189452060950

C	2.83282452700577	4.24791914000832	0.35947088884899
C	1.72032067113197	1.31935360860966	3.32435202495447
C	3.31714123924692	3.02334445926034	2.35417185828721
C	-4.52867061516229	1.38339977607343	2.41816079201224
C	-3.65633258727912	-0.47081848489300	-1.44793171939488
C	-4.66213211272211	-2.24755683095941	0.03998203903984
C	-1.84640837432442	-1.49607022648696	3.21373638646201
C	-3.81730581513418	-2.70292739169702	2.22490572478236
H	-0.14246920485397	-4.06664972003051	-4.85531501094617
H	1.37639755433068	-4.97810155645022	-4.79940422218061
H	1.40563711474536	-3.23063522089039	-5.07431107641804
H	-0.02279664781286	-5.10392850513013	-0.78004307749247
H	0.10043360633811	-6.05363797780107	-2.27295284488136
H	-1.19913338159458	-4.86258074281651	-2.08390730242953
H	3.45146128610677	-3.34689564273211	-2.37311888365932
H	3.09249022189348	-5.07760857487544	-2.26528700791428
H	2.71448545942078	-3.99891696596578	-0.90547331159672
H	4.45249369412681	0.39691850737967	-2.30546227709349
H	5.58562919923385	-0.73439815060345	-3.06870692661177
H	4.29657444433604	-1.34400900404364	-2.01937737621311
H	3.63240149138503	0.70446117480955	-6.44130315080324
H	5.13681997967403	0.70521199922134	-5.51448478035264
H	3.76046001700821	1.67540081840316	-4.96716138072246
H	3.84872083920769	-3.19825527773514	-4.55104322999613
H	5.07942865290987	-2.27141944307711	-5.41739128418877
H	3.47910358881924	-2.49822282138872	-6.13510925679230
H	2.36655968952239	-0.71387216236433	-7.28378921832273
H	0.68615517540953	-0.64643862812618	-7.81952246057873
H	1.28094522881415	-2.08424919465954	-6.98319483183409
H	-1.02828319427558	-1.95954084786758	-4.90781073080057
H	-1.53924487550017	-0.85874521035950	-6.19528093980904
H	-1.64552476523080	-0.35803359037548	-4.49553277900526
H	0.26937389952094	2.13155741782014	-4.70598398224016
H	-0.10063935511693	1.90254769668927	-6.42629806986016
H	1.58229427451973	2.07561462100576	-5.89572222131263
C	-3.52783949041979	3.83245555123357	3.52281733585113
H	0.23150593252918	4.64999219754059	2.63571947245276
C	-1.56769026322766	5.26464130502963	3.68461888154033
C	1.50668326083688	4.24240635984979	-2.25703652493812
H	0.30881662364054	2.80014922608394	-1.28381120874880
C	-0.38680069053735	4.67915238556125	-0.59885192380632
H	3.09831987074220	5.00308857869331	-0.37458083277982
C	3.68263809667392	4.00907816800999	1.44214021041736
H	0.98672573211041	0.62548285430477	2.89019518145972
C	1.02293212697755	2.06224241100110	4.46083344662404
C	2.89188774728991	0.46574992611694	3.80668882217948
H	3.98262164544400	2.80340596211421	3.18528268696379
H	-4.93538618047734	0.45489966098405	2.03560237367746
C	-5.33534093161819	2.22750573231996	3.22123950146552
H	-2.61609439145096	-0.14439337896649	-1.57122817121410
C	-4.50612889891065	0.78812711115849	-1.43678940271726
C	-3.95410789796794	-1.37829093980142	-2.63964835205681
C	-4.73848023031591	-2.98813650996685	1.21690499488977
H	-5.35188388189473	-2.46819990816986	-0.77111813232404
H	-1.41211304952992	-0.49990808338132	3.08639452503608
C	-0.68195270820391	-2.48229833652786	3.17561440949760
C	-2.53714780197576	-1.52227854270601	4.57669446559206
H	-3.84067738064536	-3.27374111416610	3.14899580346618
C	-2.88768414929912	5.00432676148603	4.01220702702540
C	-4.86447750929923	3.41234973704386	3.76626342562295
H	-1.09589513411945	6.16275040813511	4.07290045387857
C	2.69961920542503	3.60172984666935	-2.62407367084122
C	1.02922435750134	5.27261942043668	-3.07266702279556
C	-0.13753514260430	5.95168119760517	-0.07215126929078
C	-1.70721082169453	4.23531774183260	-0.68771620448893
C	4.96982284160767	4.79214907175091	1.61531676329236
C	-0.24240137355522	1.66028080890535	4.89033353094728
C	1.62447165715174	3.15335826005189	5.09876997748183
C	3.05363839327704	0.13040376051081	5.15414662484226

C	3.80163547401538	-0.05631047372578	2.87704803758043
H	-6.35920445468128	1.92453474213789	3.42214806443361
C	-4.09381629262127	1.88970278993861	-2.19646672876520
C	-5.71265607917484	0.87138198673536	-0.73693233122011
C	-3.22925861249765	-2.57149756438903	-2.76908397174936
C	-4.89959437512280	-1.06464012153411	-3.61886806968999
C	-5.76751039646457	-4.08856375717916	1.38413910849466
C	-0.68182553977004	-3.64029262415715	2.39618038582902
C	0.42485026405511	-2.22476509125071	3.99710577033891
C	-3.15987410622429	-0.36880567227280	5.06585215824446
C	-2.59816016684219	-2.69084377898211	5.34497844800891
H	-3.43749437508139	5.69363950710834	4.64788303953204
H	-5.51752530593575	4.02317920034943	4.38448270914462
H	3.07773103628207	2.78753105173712	-2.01184630362472
C	3.41746865979447	4.01489300101024	-3.74319766036023
H	0.10175436420180	5.77447951374621	-2.81376797424826
C	1.73403909199347	5.67177135488365	-4.21177649083814
H	0.88722022928434	6.30400456956044	0.01575393200506
C	-1.18745204742462	6.76265638068976	0.35279568878065
H	-1.90810078069768	3.24294334488222	-1.08212663984949
C	-2.76186568065352	5.03667667688237	-0.24713283851943
C	4.68180741227331	6.28458748151051	1.85864458673197
H	5.47345132026605	4.39229999251179	2.50481635955640
C	5.90646070077918	4.60486428318928	0.40857612746466
H	-0.72641206817703	0.82209235060224	4.40104387093999
C	-0.90353713059178	2.32959842219590	5.91994213399317
H	2.60724886863713	3.49021025617694	4.78426953028704
C	0.97208635941598	3.82566683952822	6.13036858519920
H	2.36073716714655	0.52260208370337	5.89241773044533
C	4.09456153173581	-0.70620319406259	5.56459244410350
C	4.83817370232240	-0.89482820499151	3.28070260677095
H	3.69764056895508	0.20176013312009	1.82731506892601
H	-3.16018281117445	1.82954662968064	-2.75071005009572
C	-4.86809865308369	3.04738569595326	-2.25652949555957
H	-6.05035913683841	0.02612863815246	-0.14442643128599
C	-6.48777683722827	2.03106765582147	-0.78713554884602
H	-2.49405654034521	-2.82616403016954	-2.00990463620288
C	-3.44290364099149	-3.42755751299057	-3.84445786704982
H	-5.47395874912760	-0.14656267326871	-3.53945847519257
C	-5.11625283638628	-1.92138207215480	-4.70282335756578
H	-6.38144438668375	-4.10035705984661	0.47432219140617
C	-5.08680751001872	-5.46311554131764	1.51849155781949
C	-6.69301703496333	-3.81701418975814	2.58333020378723
H	-1.52862430512711	-3.85990023916304	1.75534015271856
C	0.40622223533288	-4.51798924918649	2.42852755606463
H	0.43561019309409	-1.32942008648065	4.61237053694184
C	1.50803772900533	-3.09541873274547	4.03315722976128
C	-3.27922244167399	-2.71004775217477	6.56290383961449
H	-3.11708749219627	0.54716382965285	4.48687807379757
C	-3.83695445363218	-0.38153906575229	6.28459532943396
H	-2.10585438127053	-3.59169722662663	4.98947406459483
H	4.35846938076214	3.53096183130917	-3.98852198258847
C	2.93812449003395	5.05390155839923	-4.54501660783600
H	1.34318023772967	6.47570668559465	-4.83015616889594
H	-0.97797659802340	7.74725546733005	0.76288639052484
C	-2.50483522249708	6.30429580399400	0.27207183322348
H	-3.77963773284812	4.66282160936290	-0.30340281512859
H	4.17734616870393	6.72670278800037	0.99158802012509
H	5.61507303746653	6.83441979566018	2.02563556257813
H	4.03708690886505	6.42143421509587	2.73340702174870
H	6.13514631687242	3.54550177091885	0.25035579295193
H	6.84763140958032	5.14350076267794	0.56702180846072
H	5.44325507802381	4.99253438747438	-0.50622483658409
H	-1.89275940916610	2.00282432210726	6.22578416701989
C	-0.29756704994169	3.41779338659714	6.54434073539958
H	1.45458643183090	4.67393403802751	6.60914523347273
C	4.99067575266855	-1.22393505820707	4.62986841716475
H	4.20117407000027	-0.95335929020585	6.61768455169773
H	5.52890743175485	-1.29042660385886	2.54047154572984

C	-6.06912147419302	3.12334289183020	-1.54769645722279
H	-4.52962684918355	3.89288827229812	-2.84908655871625
H	-7.41938674504271	2.08070198115423	-0.22947835723830
C	-4.39121886042506	-3.10590435184678	-4.81998528898523
H	-2.86722919054414	-4.34577011035909	-3.92695438437161
H	-5.85772017676073	-1.65930222850668	-5.45322569595081
H	-4.44406579322890	-5.66812546182610	0.65572687529081
H	-5.83801462828138	-6.25801940296478	1.58833024185958
H	-4.46675574274802	-5.50093911070014	2.42179923650108
H	-6.12106720241090	-3.79418215717760	3.51815688060109
H	-7.45028838447253	-4.60458895741550	2.66839938838053
H	-7.20505921503299	-2.85500887852493	2.47379384991250
H	0.39330473461980	-5.40958644207438	1.80715050003459
C	1.50287681584319	-4.24951006098061	3.24379630860657
H	2.36202134020811	-2.86697432376454	4.66398171526828
H	-4.30972837858460	0.52770041876543	6.64687940313556
C	-3.90252660197352	-1.55507178675549	7.03815036659192
H	-3.31805775686919	-3.62843266068431	7.14283419181615
H	3.49790629798271	5.37557733867489	-5.41899387714135
H	-3.32255661121833	6.92748310521434	0.62364318420738
H	-0.81079198554926	3.94882841595559	7.34137224717146
H	5.80085893191382	-1.87466286138259	4.94767269677720
H	-6.67079624338290	4.02732577733406	-1.58619250625917
H	-4.56046457266618	-3.77237654425158	-5.66141319075986
H	2.35065964844377	-4.92911748050359	3.26313432165118
H	-4.42803417281821	-1.56786038485034	7.98909943662863

Selected Mayer bond orders

B(0-Co, 1-S) :	0.5148	B(0-Co, 2-P) :	0.1152	B(0-Co, 3-P) :	0.5962
B(0-Co, 4-P) :	0.6023	B(0-Co, 5-P) :	0.7655	B(0-Co, 6-N) :	0.6067
B(0-Co, 7-N) :	0.6431	B(0-Co, 10-C) :	0.1037	B(1-S , 2-P) :	1.1306
B(2-P , 3-P) :	0.9243	B(2-P , 5-P) :	0.1200	B(2-P , 8-N) :	1.1307
B(2-P , 9-N) :	1.0679	B(3-P , 4-P) :	1.1568	B(3-P , 11-C) :	0.1393
B(4-P , 5-P) :	0.9993	B(5-P , 8-N) :	0.8822	B(5-P , 13-C) :	0.1126
B(6-N , 10-C) :	1.4624	B(6-N , 11-C) :	0.5041	B(7-N , 12-C) :	1.4878
B(7-N , 13-C) :	0.5416	B(8-N , 14-Si) :	0.9525	B(9-N , 15-Si) :	0.9312
B(9-N , 16-Si) :	0.8669	B(10-C , 12-C) :	1.1445	B(10-C , 17-C) :	0.8632
B(11-C , 18-C) :	1.3677	B(11-C , 19-C) :	1.4178	B(12-C , 20-C) :	1.0060
B(13-C , 21-C) :	1.3306	B(13-C , 22-C) :	1.2937	B(14-Si, 23-C) :	1.0166
B(14-Si, 24-C) :	1.0322	B(14-Si, 25-C) :	1.0167	B(15-Si, 26-C) :	1.0334
B(15-Si, 27-C) :	1.0216	B(15-Si, 28-C) :	1.0080	B(16-Si, 29-C) :	1.0018

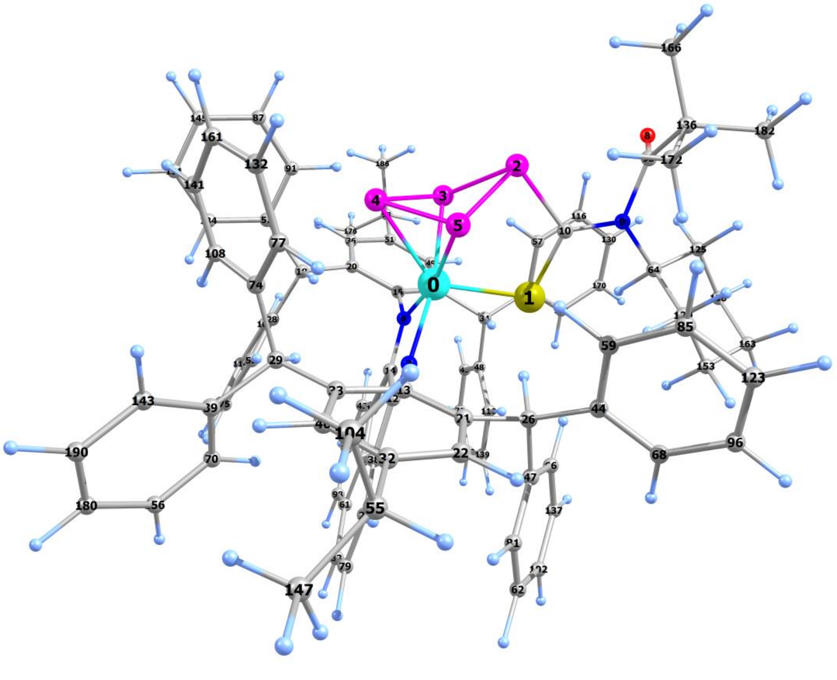

Figure S42. Optimized geometry of [(Ar*BIAN)Co(η^3:η^1-P_4C(S)N(Cy)C(O)*t*Bu)] (**6a**) at the D3BJ-PBE0/def2-SVP@C,H/def2-TZVP@Co,P,N,S CPC(Benzene) level and atom labeling.

Cartesian coordinates of the optimized geometry of [(Ar*BIAN)Co(η^3:η^1-P_4C(S)N(Cy)C(O)*t*Bu)] (**6a**) at the D3BJ-PBE0/def2-SVP@C,H/def2-TZVP@Co,P,N,S CPC(Benzene) level and atom labeling.

Co	0.06464451910644	0.05278912545616	0.03711093113424
S	2.24720515022026	-0.11091573874997	-0.40370262897884
P	1.91910580868605	-0.19273919980089	2.59981971230055
P	0.56823734319903	1.41612637869779	1.78502751683577
P	-1.12666571282865	0.06028989457554	1.99595867626874
P	0.34532747331950	-1.51382153237536	1.65859714135848
N	-0.49127094337635	1.48781009463051	-1.19125324096054
N	-0.72155464031940	-1.09912109041212	-1.37625290597672
O	5.36175273656963	0.89330833167595	2.99270877704679
N	4.35754282794314	-0.24133829223673	1.24027071759658
C	3.02111642492179	-0.21221745257880	1.11056690254058
C	-1.78632476300401	0.54027027562380	-4.42897670534360
C	-1.05327973262111	-0.40903040360409	-2.45802430112203
C	-1.01226482289554	-2.50131306923242	-1.35353747102305
C	-0.94032196201516	1.04168901692047	-2.34823577096379
C	-0.45149688254322	2.88350421250118	-0.89838314421950
C	-1.42170276804657	1.64466883236138	-3.59521835366330
C	0.72590161871987	3.63426968247363	-1.13200635844513
C	-2.87845234117937	2.69953093062278	-0.11700152663689
H	-2.59968295222906	1.62377120741675	-0.06518413221082
C	-1.57772750817770	3.48084919348291	-0.27369893364434
C	-0.00518508218900	-3.45695526669215	-1.63978642928847
C	-0.33566559242573	-4.82394853694098	-1.61322603847583
H	0.45510541992209	-5.56042550900314	-1.82641974358894
C	-1.92539962630095	-1.88697001068095	-4.48290920281175
H	-1.81564201960946	-2.87960183372674	-4.02762736967805
C	1.39699314821149	-3.01304136133722	-2.02950286383867
H	1.56709350814763	-2.04251091662281	-1.51748815315849
C	-3.74720650245982	2.86809941369730	-1.36381637907457
C	-3.44110389834174	-1.93528461570988	-0.69810038364818
H	-3.11246642796267	-0.94643943793275	-1.07167996257233
C	3.25887196015961	3.40369735762527	-1.29399603417648
C	-1.63271424545153	-5.27346812447967	-1.32083202663854
C	-2.33351740417611	-2.92841430501000	-1.04437455238772
C	1.90268766933263	3.01042938564346	-1.87380969098569
H	1.82807371973003	1.91580900708097	-1.70605205261889
C	-1.48869503785890	4.82375147331919	0.12860004056782
H	-2.35520780035583	5.27107039440637	0.63947801753627
C	-1.58854009189787	-0.72717926379055	-3.78790377897839
C	-4.74044011809553	-2.27217126408244	-1.43273975232206
C	-2.61250129833198	-4.30486851845593	-1.04227158212253
H	-3.64023369625065	-4.62659907263311	-0.81622671955384
C	-1.59077366393197	2.93729492301827	-4.07952110114249
H	-1.33196034296207	3.81368079290408	-3.47108531993650
C	2.48489452656857	-3.94379428238780	-1.49263408498939
C	1.60585211669312	4.48980918722018	-3.94974522036828
H	1.58577219555480	5.37627960352692	-3.29807654662941
C	1.53784019588471	-2.72623823715132	-3.51964173537533
C	1.76914155407367	3.21078921188892	-3.37992402500542
C	0.76369961323729	4.97574519145787	-0.71162559348180
H	1.69014203939903	5.55214941417659	-0.86398090023971
C	-0.32741951721320	5.59038171952140	-0.07567798838068
C	-3.62303403842291	2.98787798877821	1.18743337434178
C	-4.27439747650590	1.74066222003752	-2.01959628814883
H	-4.04976366028383	0.73966815807262	-1.62509313973727
C	-1.96256676109206	-6.75757265023512	-1.27789120122752
H	-1.05717384220563	-7.29885914741893	-1.62911091431232
C	3.46144257576344	3.28708690541398	0.09974336248259
H	2.62086581135908	2.97369147488902	0.73966524438758
C	2.49624198801862	-4.25775441924021	-0.11543840781370
H	1.70814046414802	-3.84556649202448	0.53562291458070

C	-2.30786355742483	0.68586232148330	-5.73511016569570
C	1.12068309714015	-3.26855757130901	-5.86662946668707
H	0.63705355397506	-3.91064988985017	-6.61890232208411
C	5.23213013199754	-0.15058400303664	0.04010923204884
H	4.68896908990535	0.54715715565524	-0.63808013099858
C	2.33181553260899	-1.63986564466810	-3.93957382267810
H	2.81563140578449	-1.00590001413470	-3.17925565683696
C	3.50549420761272	-4.46924296537826	-2.30791043326720
H	3.51647199727672	-4.23560949726898	-3.38234335708916
C	-4.93165212160315	-1.83231839632286	-2.75808751132802
H	-4.15500947994170	-1.22289011300627	-3.24133584236497
C	1.45248026896112	4.64266187263487	-5.33609519029639
H	1.32259621112861	5.64939782734800	-5.76275534396845
C	-3.67220987070028	-1.75887796369753	0.80349466873437
C	-5.06200176038462	1.87066769335149	-3.17384015369476
H	-5.45178679879209	0.96890927630480	-3.66965711889744
C	-3.12552649964982	-2.61741466977724	1.77459531032244
H	-2.48137758194322	-3.45240797789209	1.46344277235907
C	-2.44074399942132	-1.76170442754715	-5.80598989225975
H	-2.70177566032043	-2.67984002265815	-6.35390515208804
C	0.94119536685517	-3.54071493199040	-4.50114564206708
H	0.31891018858714	-4.39369163349724	-4.19099266227652
C	-2.63138274882234	-0.52507730484952	-6.42479545554414
H	-3.03675811606692	-0.47635234113349	-7.44745422521353
C	3.49327051378175	-5.08040902525112	0.42600120688433
H	3.47799853095663	-5.31825889071745	1.50046489243398
C	-3.57209343296258	3.22885099957300	3.62389737083388
H	-2.98978208511862	3.27557579024450	4.55699917561403
C	-2.10891593395294	3.10490335506634	-5.39543201716478
H	-2.23691864339066	4.12818804970492	-5.77916752832397
C	-2.90653981457738	3.05945177643705	2.40185960717567
H	-1.80778173693103	2.98589713235285	2.38825804320205
C	-0.24588014852003	7.03194535558370	0.40293722868467
H	0.73640258003687	7.42735688899970	0.06412384716440
C	4.93095252067490	-0.17320711768230	2.60635890881872
C	4.51333976155152	-5.28467001946021	-1.76372181888541
H	5.30287402048490	-5.68305594234185	-2.41971408894074
C	-2.45796662315145	2.02665656408048	-6.20984216363496
H	-2.85558058779224	2.20344689774225	-7.22122631607680
C	-4.03983099980179	4.14257846953815	-1.89060193179993
H	-3.63254446499109	5.04034944681183	-1.40230580073728
C	1.90779874828091	-2.17941826190446	-6.27377882255147
H	2.04540024051033	-1.96330418749728	-7.34426789780629
C	-2.24837375708915	-7.21178700902007	0.16472265542092
H	-3.14497079838536	-6.69866390219542	0.57141542075148
H	-1.39547929053188	-6.97992286404784	0.83383537986456
H	-2.43560926237665	-8.30459947993707	0.20724892533367
C	-4.48494910419969	-0.68575019742384	1.23254756034354
H	-4.91894897632430	-0.00118518791680	0.48711186005746
C	-5.34086605274007	3.14432110140435	-3.69117233187234
H	-5.95018608817157	3.25232566454398	-4.60138794563888
C	4.34433977239045	3.80094469843536	-2.09683113083476
H	4.21114214985406	3.88705980178714	-3.18533520774585
C	-4.83022750154259	4.28055082425246	-3.04118714990309
H	-5.04183219134192	5.28514294916944	-3.43910854277259
C	4.70839999598573	3.56449167261020	0.67441663599862
H	4.84579691107314	3.44097655335494	1.75906293713845
C	1.77651133396138	2.09219583177249	-4.23464344226815
H	1.88213734966448	1.08674236930554	-3.80052328904132
C	5.33530047484215	-1.51767221509882	-0.65860844449803
H	4.32699923691826	-1.94854392018084	-0.81362394499222
H	5.88756265404384	-2.21945540068160	0.00314614843270
C	4.51110042334980	-5.59569821778352	-0.39595189106690
H	5.29616729940766	-6.23959712646432	0.02928312169102
C	6.62213206920962	0.43478189635366	0.30124803723021
H	7.19686064144031	-0.23905696117501	0.97183341273069
H	6.53912137624671	1.41238957710492	0.81043511900956
C	1.45333305957914	3.51682410948314	-6.17718671021039
H	1.31780275893166	3.63620428487501	-7.26298003574236

C	5.78313998124896	3.97067176006328	-0.13666394432252
H	6.76442542273315	4.19014962322577	0.31140603108691
C	-3.38209067181891	-2.40650812096765	3.14185722829150
H	-2.93559944204320	-3.08275116551978	3.88692957559429
C	-5.02632067297605	3.09667515838129	1.23404238342535
H	-5.60443448973649	3.04061681318382	0.30011028219608
C	5.09619758052738	-1.48605629239665	3.39348450876679
C	2.51954857840529	-1.36822338163314	-5.30366680853086
H	3.14487889543886	-0.51504206646721	-5.60820406637193
C	1.61705269169676	2.23904950799355	-5.62121700454740
H	1.60171781092797	1.34609681424915	-6.26451285693964
C	-4.73795280707280	-0.47131759353035	2.59211742656424
H	-5.35048589703725	0.38933195815037	2.90094831106368
C	-5.75181131424793	-3.04818849700575	-0.83127711573494
H	-5.63004128787281	-3.37991225443704	0.21120242574199
C	-4.97319528534588	3.33462272545732	3.65817421937505
H	-5.49709342825302	3.46896419986766	4.61692518425212
C	-3.12294061809663	-7.11716050676048	-2.22003306552518
H	-3.30857660587944	-8.21076978328201	-2.21518239281399
H	-2.90655257167823	-6.80794993774290	-3.26252504241530
H	-4.06452962020989	-6.61795577196939	-1.90960349781251
C	-5.69658892550610	3.26903934681601	2.45760067336814
H	-6.79446969599799	3.35173825519251	2.46822850259666
C	6.07214343145211	-1.37328727958565	-1.99468582667651
H	5.46244018373456	-0.73682393847121	-2.67478322016191
H	6.15205495287013	-2.36611104391913	-2.48420866365225
C	-6.09403862366163	-2.16667369610068	-3.46917550991785
H	-6.22013267273440	-1.80955022995649	-4.50325409091953
C	7.36612116587783	0.58439125968495	-1.03502570730760
H	6.83237815637251	1.33807543540136	-1.65528936816910
H	8.37926367733545	0.99659065686805	-0.84788024601922
C	-4.18603703961649	-1.33497119678048	3.55505629698757
H	-4.37570021665516	-1.16369746662956	4.62560794387982
C	7.45659803355946	-0.74226897775262	-1.80079303540240
H	8.09988787116103	-1.44938592759958	-1.23008784344675
H	7.95383409273698	-0.58696688928931	-2.78105679865452
C	4.79075047375817	-1.19659409196078	4.87499742194516
H	3.72238833073653	-0.93184718582077	5.00332079830207
H	5.40659913827538	-0.35551371926017	5.24618841298801
H	5.01044554245477	-2.09569508549837	5.48415440027166
C	5.59630903751778	4.08597059156418	-1.52224152090859
H	6.43142779886180	4.39955658023314	-2.16770191502045
C	4.24271440344703	-2.64877195155828	2.87359292892042
H	4.48569865240818	-3.56451400749197	3.44870742499930
H	4.43215934183435	-2.86023320221887	1.80535429215844
H	3.15777212844397	-2.45699455762207	2.99937325570506
C	-1.34496617051310	7.90720279953986	-0.22169356112455
H	-1.31226751814251	7.86660010743709	-1.32928507693340
H	-1.22927968834433	8.96587344737740	0.08906676589976
H	-2.35450830826990	7.57536387107062	0.09940272691467
C	-7.09029768658438	-2.95052042098469	-2.86412587695213
H	-8.00400740582672	-3.21323484711833	-3.41890109170836
C	6.59695571433983	-1.85909755668123	3.24477315089174
H	7.24845530237685	-1.02552631832636	3.57165756310826
H	6.85251895037434	-2.12326870342901	2.19950389511558
H	6.80902195211055	-2.74374280672049	3.87791350999595
C	-0.27537967716099	7.10493641719621	1.93980708610451
H	-0.15676161400739	8.15158739537595	2.28853570199467
H	0.53694486356243	6.49637626206913	2.38527688511805
H	-1.23882538701565	6.72338405000703	2.33834991185842
C	-6.91428706581741	-3.38904900176191	-1.54168901944343
H	-7.69172076983169	-3.99723342929321	-1.05391711156402

Selected Mayer bond orders

```
B(  0-Co,   1-S ) :   0.6756 B(  0-Co,   3-P ) :   0.8753 B(  0-Co,   4-P ) :   0.6587
B(  0-Co,   5-P ) :   0.8884 B(  0-Co,   6-N ) :   0.6422 B(  0-Co,   7-N ) :   0.6420
B(  0-Co, 12-C ) :   0.1128 B(  0-Co, 14-C ) :   0.1231 B(  1-S ,   9-N ) :   0.1308
B(  1-S , 10-C ) :   1.2942 B(  2-P ,   3-P ) :   0.9688 B(  2-P ,   5-P ) :   0.9674
B(  2-P , 10-C ) :   0.9788 B(  3-P ,   4-P ) :   1.0906 B(  4-P ,   5-P ) :   1.0799
```

```
B(  6-N , 14-C ) :   1.4179 B(  6-N , 15-C ) :   0.8827 B(  7-N , 12-C ) :   1.4100
B(  7-N , 13-C ) :   0.8900 B(  8-O , 95-C ) :   2.1220 B(  9-N , 10-C ) :   1.2811
B(  9-N , 64-C ) :   0.9179 B(  9-N , 95-C ) :   0.8634 B( 11-C , 16-C ) :   1.1890
B( 11-C , 38-C ) :   1.1992 B( 11-C , 61-C ) :   1.2639 B( 12-C , 14-C ) :   1.0896
```

References

[1] Scheinost, A. C.; Claussner, J.; Exner, J.; Feig, M.; Findeisen, S.; Hennig, C.; Kvashnina, K. O.; Naudet, D.; Prieur, D.; Rossberg, A.; Schmidt, M.; Qiu, C.; Colomp, P.; Cohen, C.; Dettona, E.; Dyadkin, V.; Stumpf, T. ROBL-II at ESRF: A Synchrotron Toolbox for Actinide Research. *J Synchrotron Rad* **2021**, *28*, 333–349.

[2] a) Sheldrick, G. M.; SADABS, Bruker AXS, Madison, USA **2007**; b) CrysAlisPro, Scale3 Abspack, Rigaku Oxford Diffraction **2019**.

[3] Clark, R. C.; Reid, J. S. The Analytical Calculation of Absorption in Multifaceted Crystals. *Acta. Cryst. A* **1995**, *51*, 887–897.

[4] Sheldrick, G. M. SHELXT – Integrated Space-Group and Crystal-Structure Determination. *Acta Cryst A* **2015**, *71*, 3–8.

[5] Dolomanov, O. V.; Bourhis, L. J.; Gildea, R. J.; Howard, J. a. K.; Puschmann, H. OLEX2: A Complete Structure Solution, Refinement and Analysis Program. *J. Appl. Cryst.* **2009**, *42*, 339–341.

[6] Sheldrick, G. M. Crystal Structure Refinement with SHELXL. *Acta Cryst. C* **2015**, *71*, 3–8.

[7] Sheldrick, G. M. A Short History of SHELX. *Acta. Cryst. A* **2008**, *64*, 112–122.

[8] a) Neese, F. The ORCA Program System. *Wiley Interdiscip. Rev.-Comput. Mol. Sci.* **2012**, *2*, 73–78; b) Neese, F. Software update: the ORCA program system, version 4.0 *WIREs Comput. Mol. Sci.*, **2017**, *8*, e1327.

[9] a) Tao, J.; Perdew, J. P.; Staroverov, V. N.; Scuseria, G. E. Climbing the Density Functional Ladder: Nonempirical Meta--Generalized Gradient Approximation Designed for Molecules and Solids. *Phys. Rev. Lett.* **2003**, *91*, 146401; b) Staroverov, V. N.; Scuseria, G. E.; Tao, J.; Perdew, J. P. Erratum: "Comparative Assessment of a New Nonempirical Density Functional: Molecules and Hydrogen-Bonded Complexes" [J. Chem. Phys. 119, 12129 (2003)]. *The Journal of Chemical Physics* **2004**, *121*, 11507.

[10] a) Weigend, F. Accurate Coulomb-Fitting Basis Sets for H to Rn. *Phys. Chem. Chem. Phys.* **2006**, *8*, 1057–1065; b) Weigend, F.; Ahlrichs, R. Balanced Basis Sets of Split Valence, Triple Zeta Valence and Quadruple Zeta Valence Quality for H to Rn: Design and Assessment of Accuracy. *Phys. Chem. Chem. Phys.* **2005**, *7*, 3297–3305.; c) Weigend, F.; Häser, M.; Patzelt, H.; Ahlrichs, R. RI-MP2: Optimized Auxiliary Basis Sets and Demonstration of Efficiency. *Chemical Physics Letters* **1998**, *294*, 143–152.

[11] a) Caldeweyher, E.; Ehlert, S.; Hansen, A.; Neugebauer, H.; Spicher, S.; Bannwarth, C.; Grimme, S. A generally applicable atomic-charge dependent London dispersion correction. *J. Chem. Phys.* **2019**, *150*, 154122; b) Caldeweyher, E.; Bannwarth, C.; Grimme, S. Extension of the D3 dispersion coefficient model. *J. Chem. Phys.* **2017**, *147*, 034112.

[12] a) Tomasi, J.; Mennucci, B.; Cammi, R. Quantum Mechanical Continuum Solvation Models. *Chem. Rev.* **2005**, *105*, 2999–3094; b) Barone, V.; Cossi, M. Quantum Calculation of Molecular Energies and Energy Gradients in Solution by a Conductor Solvent Model. *J. Phys. Chem. A* **1998**, *102*, 1995–2001.

[13] Knizia, G. Intrinsic Atomic Orbitals: An Unbiased Bridge between Quantum Theory and Chemical Concepts. *J. Chem. Theory Comput.* **2013**, *9*, 4834–4843.

[14] Chemcraft - graphical software for visualization of quantum chemistry computations. https://www.chemcraftprog.com

[15] Perdew, J. P.; Burke, K.; Ernzerhof, M. Generalized Gradient Approximation Made Simple. *Phys. Rev. Lett.* **1996**, *77*, 3865–3868.

[16] a) Grimme, S.; Ehrlich, S.; Goerigk, L. Effect of the damping function in dispersion corrected density functional theory. *J. Comput. Chem.* **2011**, *32*, 1456–1465; b) Grimme, S.; Antony, J.; Ehrlich, S.; Krieg, H. A Consistent and accurate *ab initio* parametrization of density functional dispersion correction (DFT-D) for the 94 Elements H-Pu. *J. Chem. Phys.* **2010**, *132*, 154104.

[17] a) London, F. Théorie quantique des courants interatomiques dans les combinaisons aromatiques. *J. Phys. Radium* **1937**, *8*, 397–409; b) McWeeny, R. Perturbation Theory for the Fock-Dirac Density Matrix. *Phys. Rev.* **1962**, *126*, 1028–1034; c) Ditchfield, R. Self-consistent perturbation theory of diamagnetism: I. A Gauge-Invariant LCAO Method for N.M.R. Chemical Shifts. *Molec. Phys.* **1974**, *27*, 789–807; d) Wolinski, K.; Hinton, J. F.; Pulay, P. Efficient implementation of the gauge-independent atomic orbital method for NMR chemical shift calculations. *J. Am. Chem. Soc.* **1990**, *112*, 8251–8260; e) Cheeseman, J. R.; Trucks, G. W.; Keith, T. A.; Frisch, M. J. A comparison of models for calculating nuclear magnetic resonance shielding tensors. *J. Chem. Phys.* **1996**, *104*, 5497–5509; f) Stoychev, G. L.; Auer, A. A.; Izsák, R.; Neese, F. Self-Consistent Field Calculation of Nuclear Magnetic Resonance Chemical Shielding

Constants Using Gauge-Including Atomic Orbitals and Approximate Two-Electron Integrals. *J. Chem. Theory Comput.* **2018**, *14*, 619–637.
[18] Perdew, J. P.; Ernzerhof, M.; Burke, K.; Rationale for mixing exact exchange with density functional approximations. *J. Chem. Phys.* **1996**, *105*, 9982-9985.
[19] Jensen, F. Segmented Contracted Basis Sets Optimized for Nuclear Magnetic Shielding. *J. Chem. Theory Comput.* **2015**, 11, 132–138.
[20] Pritchard, B. P.; Altarawy, D.; Didier, B.; Gibson, T. D.; Windus, T. L. New Basis Set Exchange: An Open, Up-to-Date Resource for the Molecular Sciences Community. *J. Chem. Inf. Model.* **2019**, *59*, 4814–4820.
[21] Neese, F.; Wennmohs, F.; Hansen, A.; Becker, U. Efficient, approximate and parallel Hartree–Fock and hybrid DFT Calculations. A ‘chain-of-spheres’ algorithm for the Hartree–Fock exchange. *Chem. Phys.* **2009**, *356*, 98–109.
[22] a) http://www.iboview.org/index.html; b) Knizia, G.; Klein, J. E. M. N. Electron Flow in Reaction Mechanisms—Revealed from First Principles. *Angew. Chem. Int. Ed.* **2015**, *54*, 5518–5522.
